# The REFORM study protocol: a cohort randomised controlled trial of a multifaceted podiatry intervention for the prevention of falls in older people

**DOI:** 10.1136/bmjopen-2014-006977

**Published:** 2014-12-17

**Authors:** Sarah Cockayne, Joy Adamson, Belen Corbacho Martin, Caroline Fairhurst, Catherine Hewitt, Kate Hicks, Robin Hull, Anne Maree Keenan, Sarah E Lamb, Lorraine Loughrey, Caroline McIntosh, Hylton B Menz, Anthony C Redmond, Sara Rodgers, Wesley Vernon, Judith Watson, David Torgerson

**Affiliations:** 1 York Trials Unit, Department of Health Sciences, University of York, York, UK; 2 Podiatry Services, Harrogate and District NHS Foundation Trust, Harrogate District Hospital, Harrogate, UK; 3 NIHR Leeds Musculoskeletal Biomedical Research Unit, Chapel Allerton Hospital, Leeds, UK; 4 Leeds Institute of Rheumatology and Musculoskeletal Medicine, University of Leeds, Leeds, UK; 5 Nuffield Department of Orthopaedics, Rheumatology and Musculoskeletal Sciences, Kadoorie Critical Care Research Centre, John Radcliffe Hospital, University of Oxford, Oxford, UK; 6 National University of Ireland, Galway, Republic of Ireland; 7 Faculty of Health Sciences, Lower Extremity and Gait Studies Program, La Trobe University, Bundoora, Victoria, Australia; 8 Podiatry Services, Sheffield Teaching Hospitals NHS Foundation Trust, Jordanthorpe Health Centre, Sheffield, UK

**Keywords:** HEALTH ECONOMICS, STATISTICS & RESEARCH METHODS, QUALITATIVE RESEARCH

## Abstract

**Introduction:**

Falls and fall-related injuries are a serious cause of morbidity and cost to society. Foot problems and inappropriate footwear may increase the risk of falls; therefore podiatric interventions may play a role in reducing falls. Two Cochrane systematic reviews identified only one study of a podiatry intervention aimed to reduce falls, which was undertaken in Australia. The REFORM trial aims to evaluate the clinical and cost-effectiveness of a multifaceted podiatry intervention in reducing falls in people aged 65 years and over in a UK and Irish setting.

**Methods and analysis:**

This multicentre, cohort randomised controlled trial will recruit 2600 participants from routine podiatry clinics in the UK and Ireland to the REFORM cohort. In order to detect a 10% point reduction in falls from 50% to 40%, with 80% power 890 participants will be randomised to receive routine podiatry care and a falls prevention leaflet or routine podiatry care, a falls prevention leaflet and a multifaceted podiatry intervention. The primary outcome is rate of falls (falls/person/time) over 12 months assessed by patient self-report falls diary. Secondary self-report outcome measures include: the proportion of single and multiple fallers and time to first fall over a 12-month period; Short Falls Efficacy Scale—International; fear of falling in the past 4 weeks; Frenchay Activities Index; fracture rate; Geriatric Depression Scale; EuroQoL-five dimensional scale 3-L; health service utilisation at 6 and 12 months. A qualitative study will examine the acceptability of the package of care to participants and podiatrists.

**Ethics and dissemination:**

The trial has received a favourable opinion from the East of England—Cambridge East Research Ethics Committee and Galway Research Ethics Committee. The trial results will be published in peer-reviewed journals and at conference presentations.

**Trial registration number:**

Current Controlled Trials ISRCTN68240461assigned 01/07/2011.

**Strengths and limitations of this study**

- This study is the first UK study evaluating the clinical and cost-effectiveness of podiatric care combined with footwear advice and provision (if required), orthotic inserts and foot and ankle exercises for falls prevention in people over 65 years of age.
- This study uses the novel cohort randomised controlled trial design.
- The study uses an unblinded, patient self-report primary outcome measure.
- It will not be possible to determine the clinical and cost-effectiveness of this intervention in patients who fall but do not receive routine podiatry care.

## Background

Falls and fall-related injuries are a serious cause of morbidity and cost to society,^1^ a burden which will increase with an ageing population. The National Service Framework (NSF) for Older People^2^ recognises the importance of fall-related injuries, and calls for health improvement plans to be devised that will reduce this burden.

It is well recognised that falls result from interactions between environmental hazards, a variety of medical conditions and physiological risk factors.^3^ Foot problems may also increase the risk of falls. Foot problems affect one in three community dwelling people over the age of 65 years^4^ and are associated with reduced walking speed and difficulty in performing activities of daily living.^5–7^

There have been two relevant Cochrane reviews on falls prevention, one relating to falls in community dwelling older people^8^ and one focusing on falls in hospitals and aged care facilities.^9^ At the time of designing the study neither identified any randomised controlled trials (RCTs) focusing on podiatry-related interventions. A subsequent update has identified one trial of a podiatry-based intervention for the prevention of falls^10^ which related to the Australian healthcare system

There is some evidence to suggest that foot problems are associated with an increased risk of falling. Menz *et al*'s^6^ prospective study of 176 older people identified ankle flexibility, toe plantarflexor strength and plantar sensation as significant and independent predictors of balance and functional test performance. These factors were later confirmed as predictors of falling during a 12-month follow-up of that cohort, in which foot pain was also identified as a predictor of falling.^11^ Mickle *et al*'s^12^ ^13^ prospective study of 312 people over 60 years of age also found that fallers had significantly higher prevalence of foot pain, and displayed significantly less strength of the hallux and were more likely to have hallux valgus and lesser toe deformities.

Inappropriate footwear may also impair balance and increase the risk of falling. Footwear characteristics considered detrimental to balance include high heels, soft soles and inadequate slip resistance.^14^ ^15^ Prospective studies have shown that walking barefoot or wearing only stockings inside the home and wearing shoes with an increased heel height and smaller contract area increases the risk of falling.^16–18^ People who have a history of falling are at increased risk of falls.^19^

Given the emerging evidence that foot problems and inappropriate footwear increase the risk of falling, it has been suggested that podiatry may have a role to play in falls prevention, with several guidelines recommending that older people have their feet and footwear examined by a podiatrist.^20^ ^21^

Several studies have also suggested that some treatments provided by podiatrists, such as lesion debridement,^22^ foot orthoses,^23^ foot and ankle exercises^24^ ^25^ and footwear advice may play a role in improving balance. Combining these therapies should allow for stability and improved function to be achieved at each level. Lesion debridement can improve function during gait if pain is reduced, exercise programmes focus on internal strengthening and flexibility and appropriate footwear with orthotic devices can provide external support, improved kinaesthesia and improved function. A trial conducted in Australia in 305 community dwelling older people with disabling foot pain showed a 36% reduction in falls for those receiving a multifaceted podiatry intervention, consisting of a foot orthosis, advice on footwear, subsidy for new footwear, a home-based programme of foot and ankle exercises, a falls prevention education booklet and routine podiatry care for 12 months.^10^ ^26^ The control group received only routine podiatry care for 12 months which consisted of toenail maintenance and scalpel debridement of hyperkeratotic lesions (corns and calluses). There have been no further large pragmatic trials looking at podiatric care combined with footwear advice, foot and ankle exercise and orthotic inserts for falls prevention, and there has been no assessment of whether such an intervention is economically viable within the UK healthcare setting. The REFORM (REducing Falls with ORthoses and a Multifaceted podiatry intervention) trial aims to address these issues. The main objectives are to investigate the clinical and cost-effectiveness of a multifaceted podiatry intervention for falls prevention and to assess views and experiences of the intervention and the trial process from the perspective of the participants and podiatrists.

## Methods and analysis

### Design

The REFORM study is a ‘cohort randomised controlled trial’ (cRCT). First we will recruit participants to the REFORM cohort and while this is being assembled, we will invite a selection of eligible participants from the site undertaking the pilot phase to take part in a REFORM pilot study. Once the pilot study is complete, we will then invite the remaining eligible participants to take part in the main REFORM trial. The REFORM main trial is a two arm, pragmatic, open, multicentre, randomised controlled trial with a 12-month follow-up.

### REFORM study objectives for the pilot phase

1. To demonstrate the feasibility of recruiting to the REFORM cohort.
2. To develop and pilot the multifaceted podiatry intervention including a foot and ankle exercise programme, supplementary DVD and booklet with approximately 60 participants.
3. To develop the podiatrist training package.
4. To pilot the falls calendar and other patient data collection questionnaires.
5. To assess participants’ views and experiences of the intervention and the trial process.
6. Pilot, review and refine if necessary recruitment methodology for the main trial.

All these objectives were achieved and the pilot has moved seamlessly into the main REFORM trial.

### REFORM study objectives for the main study

This study will aim to address the following:

1. To train the podiatrists to deliver the intervention.
2. To examine the clinical and cost-effectiveness of the multifaceted podiatry intervention for falls prevention.
3. To assess the podiatrists’ views and experiences of the intervention and trial process.

### Participants

#### Participant recruitment

Participants will be recruited from NHS podiatry clinics based in either primary or secondary care in the UK and one international site in the Republic of Ireland. The rationale for recruiting only from podiatry clinics is due to the logistical constraints imposed by the requirement for the control group to receive routine podiatry care. While it would be possible to recruit from GP practices, many of the patients identified in this setting would not be receiving routine podiatry care, and NHS podiatry service managers indicted that they would not have the capacity to see large numbers of additional patients.

Podiatrists within the NHS Trust or clinic at the Republic of Ireland or the REFORM research podiatrist will undertake a search of either electronic or paper patient medical notes to identify potential participants for the study. Patients aged 65 years and over who are registered with the service and have attended routine podiatry services within the past 6 months of the search being undertaken will be identified and will be eligible for an invitation mailing. Patients who have attended high-risk clinics, (eg, diabetes clinics), or who live in a nursing home will be excluded from the invitation mail out. Sites will be requested where possible to screen out patients in the following groups: patients with a life expectancy of less than 6 months; patients known to have dementia, a neurodegenerative disorder, neuropathy, a lower limb amputation or are chair or bed bound. All eligible patients will be sent an invitation pack (letter of invitation, participant information sheet, consent form, screening questionnaire and prepaid envelope) asking whether they would like to participate in the REFORM study. Where the clinic has the capacity, potential participants attending routine podiatry clinics may be approached opportunistically. Where a multidisciplinary team exists, other healthcare professionals such as occupational therapists, falls practitioners and physiotherapists may support the opportunistic recruitment.

Participants wishing to take part in the REFORM study will be asked to return their completed consent form and screening questionnaire by post to the York Trials Unit (YTU). Researchers at the YTU will assess the returned screening forms for participant eligibility. During the consenting stage, potential participants will be informed of the possibility of participating in other related studies, for example, an associated qualitative study and they will be asked to indicate (by ticking a box on the consent form), if they would prefer not to be approached about these studies. Participants can withdraw from the study at any point. The reason for withdrawal will not have to be declared; however, if provided, this will be recorded. Data will be retained for all participants up to the date of withdrawal, unless a participant specifically requests their details be removed. All data returned to the York Trials Unit by participants will be held in accordance with the Data Protection Act 1998.

### Inclusion criteria for the REFORM cohort

All eligible, consenting participants will be asked to complete a baseline questionnaire. Participants who return valid baseline data will be included in the REFORM cohort. Any participant reporting a score of 10 or more on the Geriatric Depression Scale^27^ ^28^ that is, more severe depression, will be referred to their general practitioner (GP).

### Exclusion criteria for the REFORM cohort

Participants will be ineligible for the REFORM cohort if they are under 65 years of age; report having neuropathy, dementia or other neurological condition such as Parkinson's disease, Alzheimer's, multiple sclerosis, Lou Gehrig's/amyotrophic lateral sclerosis or Huntington's disease; are unable to walk household distances (10 m) without the help of a walk aid such as a Zimmer frame or walker or person to assist; have had a lower limb amputation; or are unwilling to attend their podiatry clinic.

### Inclusion criteria for the REFORM trial

Participants will be eligible for the REFORM trial if they:

1. Have had one fall in the past 12 months; or one fall in the past 24 months requiring hospital attention; or report a fear of falling on their baseline questionnaire that is, have worried about falling at least some of the time, in the past 4 weeks;
2. Are community dwelling.

### Exclusion criteria for the REFORM trial

Participants who do not complete the baseline or run-in data collection instruments adequately, or who are unable to read or speak English will be excluded from the trial.

### Participants who do not wish to take part in the study

Participants who do not wish to take part in the main study are not required to return any forms to the YTU. However, all participants in the pilot phase of the study who are sent an invitation pack will be given the opportunity to decline participation and if willing, provide some demographic information and reason for declining. This will provide us with sufficient information to document the reasons why participants do not wish to take part in the study and will allow us to compare decliners to those who are participating. The recruitment pack in the main mail out will not contain a decline form.

### Sample size

#### The REFORM cohort

We propose to recruit up to 2600 participants into the REFORM cohort to allow us to sample for the pilot trial and to allow cohort attrition before we sample for the main REFORM trial.

#### Internal pilot phase

Pilot trial: In order to pilot the intervention, a random sample of 60 participants will be selected from the REFORM cohort. Participants will be allocated equally to each of the two groups that is, 30 participants per group. The allocation sequence will be computer generated by the YTU randomisation service and will be stratified by centre.

#### REFORM main trial

The REFORM trial is designed to detect a 10% point reduction in the number of people who fall over a 12-month period. Assuming this high-risk group has an underlying risk of falling in a 12-month period of 50%^29^ then in order to observe a reduction to 40% with 80% power and a two-sided 5% significance level we would require 890 participants (445 in each group, allowing for a 10% loss to follow-up).

### Randomisation

Participants who fulfil the eligibility criteria for the REFORM trial and who have provided written consent to take part in the study will be eligible for randomisation. Randomisation will be carried out by the YTU secure remote computer randomisation service. Eight hundred and ninety participants will be randomly allocated in a 1:1 ratio to either the intervention or control group. If more than 890 eligible participants are recruited then we will randomly allocate the remaining participants in a 2:1 ratio in favour of the control group. This will allow us to increase the power of the study, without putting patients at any additional risk and without incurring additional costs. In order to allow for manageable case-loads at individual sites, the randomisation is by permuted blocks and stratified by centre. The YTU will write to the participant's GP informing them of study participation and to participants who are allocated to the intervention group.

### Blinding

Owing to the nature of the intervention, blinding of participants will not be feasible. Blinding of members of the study team who are actively involved in the administration of the study and may collect primary outcome falls data or undertake data queries on secondary outcomes, or the health economist will not be possible. Members of the study team responsible for data entry and the statistical analysis of the study will be kept blind to group allocation.

### Intervention group

Participants allocated to receive the intervention will be seen by the podiatrist at the clinic they would normally attend for routine podiatry care, as soon as possible after randomisation. Participants are invited to attend two podiatry visits approximately 2–4 weeks apart, with further appointments if required in addition to their usual podiatry care.

#### Footwear advice and provision

Participants’ everyday footwear (indoor and outdoor footwear) will be assessed according to the following characteristics: appropriate size; method of fastening; height and width of the shoe's heel; thickness of outsole; heel counter stiffness; longitudinal sole rigidity; sole flexion point and tread pattern. Footwear will be deemed to be inappropriate if the shoes have any of the following characteristics: (1) heel height is greater than 4.5 cm; (2) no adjustable fixation of the upper; (3) no heel counter or the heel counter can be depressed to greater than 45°; (4) a fully worn/smooth/thin sole; (5) the shoe heel width is narrower than the participant's heel width by greater than or equal to 20%; or (6) incorrect shoe size.^10^ Participants with inappropriate footwear will be counselled regarding the hazardous footwear feature(s) identified during the assessment and will be advised on safer characteristics for future footwear purchases. Where possible, footwear will be provided for participants whose footwear is deemed to be inappropriate. The podiatrist will order shoes directly from two companies participating in the Healthy Footwear Guide (HFG) scheme^30^; DB shoes (Rushden, UK) or Hotter company (Skelmersdale, UK). As not all of the shoes manufactured by these companies fulfil the characteristics of a safe shoe, the trial team will assess the shoes and provide a list of suitable footwear from which participants can choose. In order to avoid incentivising patients to take part in the study, participants will only be told about footwear provision, if they are deemed to need new footwear. During the REFORM pilot trial we will explore the feasibility of making a footwear assessment, providing footwear advice and provision of footwear and if necessary we will revise the intervention if needed.

#### Foot orthoses

**Figure 1:**
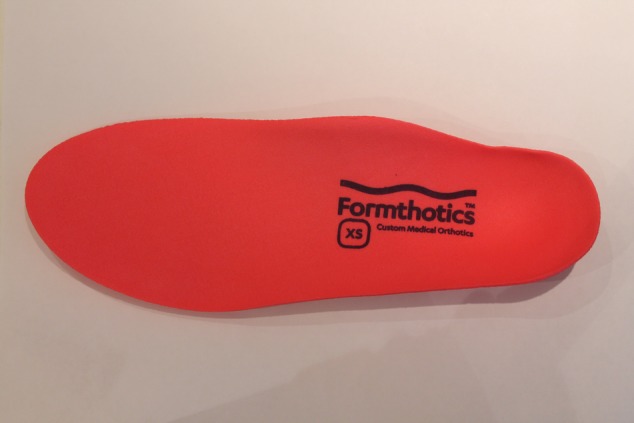
Formthotic orthotic device (colour for on-line version/monochrome for other format).

**Figure 2:**
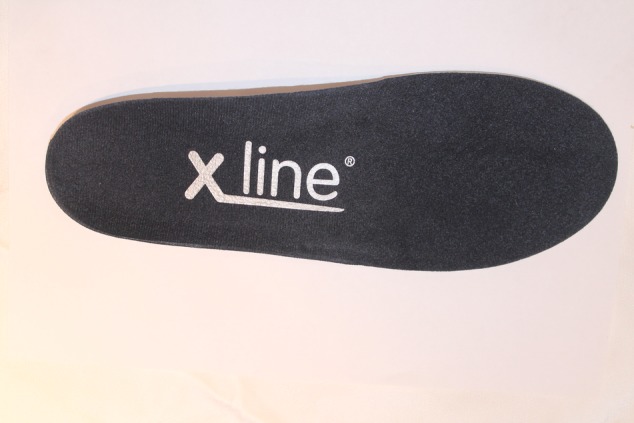
X-line orthotic device (colour for on-line version/monochrome for other format).

We originally planned to use the same orthotic device (Formthotics, Foot Science International, Christchurch, New Zealand) (figure 1) as our Australian collaborator^10^ ^26^ as they had demonstrated that this device, commonly used in Australia, was acceptable to patients and was shown to be associated with a reduction in falls. However, during the setup period of the pilot phase of the REFORM trial, podiatrists at the recruiting sites reported difficulties when fitting these devices with the addition of posting (the application of wedges under the forefoot or rearfoot) and the ease of fitting in patient's current shoes. These sites frequently use the x-line orthoses range (Healthystep, Mossley, UK; figure 2) as they are easy to modify and are easy to fit in patient's current shoes. During the pilot trial we will provide participants with both types of device to determine which device is most acceptable to a UK population. The Formthotics device will be issued as per the manufacturing guideline using the appropriate heating equipment and fitting procedure. The x-line PressurePerfect/Standard device will be fitted and if indicated, modified to improve foot posture as assessed in usual clinical practice by the individual clinician. If a participant already has insoles, whether shop bought or prescribed, the clinician will make a clinical judgement on the suitability of replacing their current insole with the trial insole. If the current insole is replaced with the trial insole then any current prescription/modifications maybe repeated, if applicable. We would consider the insole component of the intervention already addressed if the clinician felt it detrimental to replace their current insole with that of the trial insole. If the podiatrist feels that the patient requires more than what the current or trial insole can provide, or if they present with a musculoskeletal pathology, then a referral will be made in line with routine practice.

#### Home-based foot and ankle exercise programme

Participants will be prescribed a 30 min home-based foot and ankle exercise programme which will be undertaken three times per week, indefinitely. The exercise programme is aimed at stretching and strengthening the muscles of the foot and ankle. The exercises will be based on the programme developed by Spink *et al*^10^ ^26^ and will be adapted to make it suitable for a UK setting during the pilot phase of the trial. A summary of the individual exercises is given in table 1. The exercises will be demonstrated by the podiatrist and will be supplemented by a DVD and an illustrated explanatory booklet. Participants will be assessed for competence and safety at the baseline and follow-up appointments.

**Table 1:** Summary of the home-based foot and ankle exercises

Activity	Description	Dosage	Increments
Ankle range of motion/warm up	Sitting with the knee at 90°. Lift the foot to clear the ground and then rotate the foot slowly in a clockwise direction and then an anticlockwise	1×10 repetitions for each foot in each direction	None
Ankle inversion strength	Sitting upright, hip, knee and ankle at 90°. Invert foot against resistive exercise band. The band should be fixed at 90° to the foot from an additional chair/table leg	3×10 repetitions for each foot	Increase resistance strength of resistive exercise band
Ankle eversion strength	Sitting upright, hip, knee and ankle at 90°. Evert foot against resistive exercise band The band should be fixed at 90° to the foot from an additional chair/table leg	3×10 repetitions for each foot	Increase resistance strength of resistive exercise band
Ankle dorsiflexion strength	Sitting, hip, knee and ankle at 90°. Dorsiflex both feet to end range of motion and hold. Keep pulling feet up towards the body during the hold	Hold feet in dorsiflexion for 3×10 s	Increase repetitions up to maximum of 10
Intrinsic strengthening, toe plantarflexion strength and toe stretch	Sitting, hip, knee and ankle at 90°. Use the therapy ball under the toes to stretch the toes. The rest of the foot should be plantargrade. Then curl and point the toes up and over the ball. Use the therapy ball under the toes to stretch the toes. The rest of the foot should be plantargrade. With the heel on/close to the floor, curl the toes over the ball and attempt to pick up the ball with the toes	3×10 repetitions for each exercise both feet. Have a 30 sec break between each repetition	Increase up to a maximum of 50 repetitions
Ankle plantarflexion strength	From standing position, rise up onto toes of both feet and then slowly lower back down. Just before the heels contact the floor, rise back up onto the toes	3×10 repetitions	Increase repetitions up to maximum of 50
Calf stretch	Facing a wall and using hands on the wall for balance, step one foot in front of the other keeping feet hip width apart and hips, knees and feet facing the wall. Bend the knee closest to the wall and keep the back leg straight. Keep both heels in contact with the floor	Hold stretch for 3×20 s on each leg	Increase the stride length and forward lean to increase the stretch
Proprioception/balance training	From a standing position and holding on to a work surface/chair/wall for support, stand on one leg. Repeat on the other side	Hold for 30 s. Repeat 3 repetitions	Increase slowly to hold for 1 min per repetition. If competent, rise up on to toes on the one supporting leg: 3×10 repetitions

#### Routine podiatry care

Routine podiatry care will continue to be given at separate podiatry appointments in accordance with participant's usual care. This will aim to reduce painful conditions such as corns and callouses, that have been found to be associated with an increased risk of falls.

#### Falls prevention leaflet and trial newsletter

Participants will receive a copy of the latest falls prevention advice leaflet produced by Age UK (the current version ‘Staying steady’ was printed in June 2010). This leaflet will be sent to the participant in the post with their baseline questionnaire.

Participants will be sent a site and group specific newsletter at 3 and 12 months informing them about progress of the study. The intervention group's newsletter will include a section about the foot and ankle exercises in order to aid compliance. All participants will receive £5 in recognition of their participation and to offset any incidental expenses associated with completing the questionnaires.

### Control group

Participants allocated to the control group will receive the same falls prevention advice leaflet produced by Age UK. This leaflet will be sent to the participant in the post with their baseline questionnaire. Participants will continue to receive routine podiatry and GP access in accordance with their usual care. Participants will also be sent a site and group-specific newsletter at 3 and 12 months. All participants will receive £5 in recognition of their participation and to offset any incidental expenses associated with completing the questionnaires.

### Primary outcome measure for the REFORM trial

The primary outcome is the rate of falls (ie, falls/person/time) where a fall is defined as ‘an unexpected event in which the participant comes to rest on the ground, floor, or lower level’.^31^ Data will be collected via participant self-reported monthly falls calendars throughout the 12 months following randomisation. Participants will be asked to record each day whether or not they had any fall. Participants who do not return their falls calendar within 1 week of the due date will be telephoned by the YTU to obtain the missing data. Participants will also be given a Freephone number to report any falls as soon as possible after the fall. Information collected includes: date and location of fall; reason for fall; any injuries sustained (eg, a superficial wound or a broken bone); hospital admissions; footwear worn at the time of the fall; and if the patient was wearing an orthotic or using a walking aid. As we are collecting falls data at 6 and 12 months follow-up, these data will be used for those participants who do not return their monthly falls calendar.

### REFORM trial secondary outcome measures

All secondary outcomes are self-reported by the patient and collected by questionnaire at baseline, 6 and 12 months, or by monthly falls calendars. Secondary outcomes include: proportion of fallers (single and multiple); patient reported time to first fall during follow-up; health-related quality of life as measured by the EuroQoL-five dimensional scale (EQ-5D)^32^; fear of falling as measured by the question, ‘During the past 4 weeks have you worried about having a fall?’, fear of falling as measured by the Short Falls Efficacy Scale—International (FES-I),^33^ activities of daily living as measured by the Frenchay Activities Index (FAI),^34^ fracture rate, health service utilisation and depression as measured by the short form Geriatric Depression Scale (GDS).^28^

### Nested qualitative study

A qualitative evaluation will be carried out to examine the acceptability of the intervention as a whole package of care, to both the trial participants and the podiatry practitioners. Trial participants from the pilot phase of the study who are receiving the intervention will be asked about their experience of the intervention. Particular attention will be paid to the acceptability and compliance with the foot orthosis, exercise programme, podiatry service and footwear advice/purchase and how the intervention fits into the individual's wider experience of balance problems within their everyday lives.

Ten to 15 participants will be purposively selected from the pilot phase of the trial (the first 60 participants randomised) to ensure a representative spread according to falls history, age and gender. This maximum variation sampling approach^35^ will ensure a wide range of viewpoints are included in the data collection and analysis. Participants will be invited to attend a face-to-face interview or, if prefered, a telephone interview. Written informed consent will be obtained from the participant prior to the interview. The semistructured interviews will be conducted within approximately 2 weeks of receiving the 6-month follow-up questionnnaire. A follow-up telephone interview will be conducted within 2 weeks of receiving the 12-month follow-up questionnaire to discuss any changes (or not) recorded in the outcomes measured over the longer term follow-up period. All interviews will be conducted using a topic guide to ensure consistency across participants. However, the format will be flexible in order to allow participant-led data regarding what they constitute as important and/or successful in terms of treatment outcome.

We will interview a purposive sample of 5–10 practitioners who are providing the podiatry services for the study regarding the way in which they have delivered the intervention, the ease of delivery, their confidence in the intervention, and their experience of being involved in the trial. Face-to-face or telephone interviews will be conducted approximately 3 months into the trial process. Informed consent will be obtained prior to the interview being conducted.

### Adverse events

Details of any adverse events reported to the YTU either directly by the participant or by a member of the research team at the recruiting site will be recorded.

This study will report details of any serious adverse events (SAEs) that are required to be reported to the Research Ethics Committee (REC) under the current terms of the Standard Operating Procedures for RECs.

A SAE is defined as any untoward occurrence that:

1. Results in death;
2. Is life threatening;
3. Requires hospitalisation or prolongation of existing hospitalisation;
4. Results in persistent or significant disability or incapacity;
5. Consists of a congenital anomaly or birth defect, or
6. Is otherwise considered medically significant by the investigator.

An event is defined as ‘related’ if the event was due to the administration of any research procedure. An ‘unexpected event’ is defined as a type of event not listed in the protocol as an expected occurrence. Expected events include: aches and pains in the lower limb lasting for longer than 48 h; fall; new callus/corn formation, blisters, ulcers; skin irritation/injury including pressure sores and soft tissue injury.

In the context of this study, an occurrence of the type listed in (1) to (6) will be reported as an SAE only if:

1. The event is suspected to be related to an aspect of the research procedures (eg, wearing the orthotic, undertaking the exercise programme, completion of follow-up questionnaires, participation in feasibility or qualitative substudies, telephone contact), and;
2. It is an unexpected occurrence. Hospitalisations, disabling/incapacitating/life-threatening conditions, falls and deaths are expected in the study population due to the age of the cohort, they will therefore only be reported as SAEs if they appear to be related to an aspect of taking part in the study.

### Other data collected

Treatment details will be recorded by the podiatrist including: the number of podiatry visits; an eligibility checklist with details on relevant health conditions and test results; characteristics of current indoor and outdoor shoes; details relating to shoes ordered; details on the type and prescription of any current insole use; the type of insole issued/retained with any modifications made, and any amendments or advice given on the intervention due to safety reasons.

Information on adherence to the exercise, footwear advice and orthotic components of the intervention will be collected from participant self-reported questionnaire data at three, 6 and 12 months. Participants will be asked whether during the past month, they were wearing their orthotic ‘all of the time’, ‘most of the time’, ‘some of the time’, ‘a little of the time’ or ‘none of the time’. Participants will also be asked, for the past month, typically how many times a week they did the exercises: none, one, two, three or more than three times. Participants will be asked at 12 months if they were given footwear advice, and whether or not they followed any given advice.

### Statistical analysis

There will be a single analysis at the end of the trial, conducted using STATA (StataCorp, 4905 Lakeway Drive, College Station, Texas 77845, USA). All analyses will be conducted on an intention-to-treat basis, that is, including all randomised patients in the groups to which they were assigned. All tests will be two-sided at the 5% significance level. If more than 890 participants are recruited and unequal allocation is utilised, then analyses will be adjusted to take this into account.

- The REFORM cohort

Descriptive statistics will be presented for participants in the REFORM cohort.

- REFORM trial

Participant baseline data will be summarised descriptively by randomised arm. No formal statistical comparisons will be undertaken. Continuous measures will be reported as means and SDs while the categorical data will be reported as counts and percentages.

- Statistical analysis of the REFORM trial primary outcome

The number of falls per person will be analysed using a Poisson regression model adjusting for gender, age, centre and history of falling to estimate the difference in falls rate between the groups. If there is over dispersion, a negative binomial regression model adjusting for the same factors will be used.^36^ Point estimates and their associated 95% CIs will be provided.

- Statistical analysis of the REFORM trial secondary outcomes

The proportion of fallers versus non-fallers in each group will be compared by logistic regression adjusting for gender, age, centre and history of falling. ORs and their associated 95% CIs will be provided. The proportion of multiple fallers versus single or non-fallers in each group will be compared over the 12-month trial period using logistic regression adjusting for gender, age, centre and history of falling.

The time to the first fall will be derived as the number of days from randomisation until the patient reports having a fall as detailed from the participant's falls calendar, falls telephone data collection sheet or self-reported questionnaire. Participants who have not had a fall will be treated as censored at their date of trial exit, or date of last available assessment or 365 days/trial cessation, as appropriate. The proportion of patients yet to experience a fall will be summarised by a Kaplan-Meier survival curve for each group. The time to first fall will be analysed by Cox proportional hazard regression adjusting for gender, age, centre and history of falling. HRs and their associated 95% CIs will be provided. The proportional hazard assumption will be evaluated using Schoenfeld residuals. The median time to the first fall and its associated 95% CIs will be estimated from this adjusted model.

Patients with a score of six or more on the short form Geriatric Depression Scale will be categorised as having depression. The proportion of people with depression in each group will be compared over the 12-month trial period using logistic regression adjusting for gender, age, centre and history of falling.

The following secondary outcomes: Short Falls Efficacy Scale—International, fear of falling in the past 4 weeks, and the Frenchay Activities Index will be treated as continuous data and will be measured at baseline, month 6 and 12. A covariance pattern model incorporating all post randomisation time points will be used to compare the two groups on these outcomes adjusting for baseline score, gender, age, centre and history of falling. The correlation of observations within patients over time will be modelled and the model effects and their associated 95% CIs will be provided.

The proportion of patients obtaining at least one fracture over the 12-month follow-up period will be compared by logistic regression adjusting for gender, age, centre and history of falling. ORs and their associated 95% CIs will be provided. If there are patients that obtain multiple fractures in this time (from different events), then the proportion of patients obtaining multiple fractures versus single or no fractures in each group will be compared over the 12-month trial period using logistic regression adjusting for gender, age, centre and history of falling.

- Intervention adherence

Descriptive statistics will be used to summarise the adherence to the exercise and orthotic at 3, 6 and 12 months and adherence to following footwear advice. A Complier Average Causal Effect (CACE) analysis to assess the impact of compliance on treatment estimates will be considered.

- Missing data

The amount of missing data will be reported for each randomised arm.

- Qualitative analysis

All interviews will be audio recorded digitally and transcribed verbatim. The computer package ATLAS-ti will be used to manage the data. Data will be analysed according to the constant comparison method through thematic coding of the data.^37^ Coding will take place using a combination of a priori themes (according to the aims of the qualitative study and the outcome measures of interest to the trial) and emergent themes. However, in addition, as participants in the qualitative sample would also have responses to the quantitative data collected for the trial, this will allow for the possibility of taking a mixed-methods approach to data integration, in which the two forms of data can be used in a complementary way.^38^

- Adverse event data

Adverse event data will be summarised descriptively by randomised arm.

### Economic evaluation

The economic analysis will be performed using individual patient level data from the REFORM trial. The analytical approach will take the form of cost-effectiveness and cost-utility analyses. The cost-effectiveness approach will assess value for money in terms of cost per fall averted, and the cost-utility analysis will assess cost per quality adjusted life year (QALY) gained. The perspective for both analyses will be that of the UK NHS and Personal Social Services^39^ as well as that of society. Discounting for future cost and health benefit will not be included considering the time frame for the trial is 12 months after randomisation. The year of pricing will be set as the mid-year of the trial.

Health benefits associated with the treatments will be measured in terms of both estimates of the mean number of falls, corresponding to the main outcome of the trial, and mean QALYs, which is defined as a year lived with full health. The EQ-5D^40^ will be used to elicit patient utility values at different points in time and used to calculate QALYs for each patient using the area under the curve.^41^ These utility values are used as ‘quality adjustment’ for each patient's survival time.

Mean within-trial estimates of cost and health benefits will be estimated using the regression approach to allow for the correlation between costs and effects as well as adjusting for covariates. This analysis will also account for skewness and censoring associated with time to event and cost data.^42–44^ The result will be presented as incremental cost-effectiveness ratio (ICER) where the difference of mean cost estimates between two arms are divided by the difference of mean health benefit between two arms. The Net Monetary Benefit (NMB) will be also presented.^45^ The NMB provides an estimation of the gain (or loss) in resources of investing in a particular intervention when those resources might be used elsewhere.^46^

The uncertainty surrounding the decision to accept a treatment as the most cost-effective will be explored in cost-effectiveness acceptability curves (CEAC).^47^ These curves depict the probability of accepting a treatment as being cost-effective for a large range of willingness to pay values for an extra unit of health benefit. Sensitivity analysis will be conducted to explore the impact of underlying assumptions of the model and the range of unit costs on the cost-effectiveness results.

Despite the careful design of any trial the presence of missing data is unavoidable. The reasons for the presence of incomplete data are multiple. Similarly there are different methods to handle its analysis.^48^ The pattern of missing data for the economic analysis will be examined and described. The multiple imputation (MI) process has been recommended as the appropriate method to address the uncertainty in the results of economic evaluation due to missing data. Therefore, the base case analysis will be based on the MI, while the complete case data set will be used for the sensitivity analysis.

The main outcome of the REFORM trial, fall reduction, could be regarded as an intermediate outcome to achieve the final target—the reduction in fracture. However, due to the restriction in the length of follow-up, the long-term effect of decreasing the number of fractures, might not be observed within the timeframe of the current trial. Therefore, a further analysis is planned to model the possible long-term impact of the trial assuming that a falls reduction should also lead to a fracture reduction.

A decision analytic model approach will be adopted to perform such a task. The perspective will be the UK NHS and Personal Social Services and the time horizon for this analysis will be a life-time horizon. Life-time horizon refers to following up every single participant in a hypothetical cohort until the last participant dies. The hypothetical cohort will be constructed, based on the characteristics of the trial population, to estimate the QALY yield and cost saving of the long-term effect of the intervention. The model parameters which are not collected in the trial will be extracted from the existing literature. The model outputs will be the estimated expected mean costs, effectiveness, and QALYs associated with each alternative treatment. Estimated total costs and outcomes will be discounted properly according to the current guidance of health technology appraisal.^39^

Uncertainty regarding cost-effectiveness will be evaluated using probabilistic sensitivity analysis, where inputs into the analysis are defined as probability distributions which reflect uncertainty. The uncertainty surrounding the decision to adopt a given treatment option as a cost-effective treatment at different levels of willingness to pay will be represented in acceptability curves. The impact of assumptions undertaken in the analysis regarding the evidence over parameters or relating to the decision model (such as extrapolation) will be evaluated in a sensitivity analysis, if possible.

## Ethics and dissemination

### Ethics

All participants will give written informed consent prior to entry to the study.

### Dissemination

Results of the study will be disseminated through, national and international research conferences and in articles published in international peer-reviewed journals.

## Discussion

This study uses the cohort randomised controlled trial (cRCT) design. Existing trial designs have reported issues around recruitment, ethics, patient preferences and treatment comparisons.^49^ We chose this approach to find out whether the cRCT design can overcome these issues and to test its feasibility. We anticipate that using this design will offer the following advantages. First, we expect that recruitment rates will be enhanced. Some participants enrolled in the cohort will be immediately eligible for the main trial and can be randomised straight away. Others, however, will become eligible over time due to the fact that they have subsequently had a fall. If an alternative design had been chosen then these patients could not have been included in the study and would have been lost. Second, in this study participants are receiving routine podiatry care outside of the trial and apart from altruistic reasons the only incentive to take part in the study is the possibility of receiving the extra package of podiatry care. If a pragmatic design had been used, then the control patients would have been notified of their group allocation, and this may have resulted in a higher attrition rate. If this had occurred then there is the possibility that selection bias could be introduced. In addition to this, participants may also either knowingly or unknowingly bias the trial by reporting the number of falls they have had less conscientiously than those allocated to the intervention arm. In the cRCT design, participants are only notified of their allocation to the intervention group. Control participants are only aware of their participation in the study as a whole, thereby minimising the possibility of introducing attrition and reporting bias. Third, we expect that using the cRCT design will help minimise postrandomisation attrition rates. Unfortunately, many trials lose participants, and unless this occurs in a random manner, there is the possibility of selection bias being introduced. Many participants are lost in the early stages of a study for example, participants change their mind. Using the cRCT design, which effectively has a run-in period, allows the majority of patients to withdraw from the study prior to randomisation, thereby minimising postrandomisation attrition rates. Finally, one key feature of the cRCT design is the capacity to undertake multiple randomised controlled trials over time. Once this cohort is set up, we will apply for funding to undertake further trials using the participants in this study. This is not only a quick and cost-effective strategy to identifying trial participants but has the added advantage of reducing heterogeneity of trial populations enabling synthesis of trial data and indirect comparisons between trial treatments.

Falls in older people are a major health problem. Podiatry may have a role in preventing falls as there is some evidence to suggest that foot problems and inappropriate footwear may increase the risk of falling. A recent trial by Spink *et al*^10^ ^26^ has demonstrated that a podiatric intervention consisting of: footwear assessment and provision; foot orthotic device and a home-based foot and ankle exercise programme can reduce falls in patients aged over 65 years of age with disabling foot pain. As far as we are aware, such a multifaceted podiatry intervention has not been evaluated in a UK and Republic of Ireland setting. The REFORM trial aims to evaluate the clinical and cost-effectiveness of such a package of care in patients over the age of 65 years who have an increased risk of falling.

## Trial status

Recruitment and follow-up are in progress. Recruitment to the study began in October 2012 and will continue until approximately spring 2015. Patients will continue to be followed-up until approximately spring 2016.

## Supplementary Material

Author's manuscript

Reviewer comments
