# Supplementary material for: The REFORM study protocol: a cohort randomised controlled trial of a multifaceted podiatry intervention for the prevention of falls in older people
Source: BMJ Open. 2014 Dec 17;4(12):e006977. doi: 10.1136/bmjopen-2014-006977 (PMC4275677; doi:10.1136/bmjopen-2014-006977)
Supplement: Author's manuscript [file bmjopen-2014-006977.draft_revisions.pdf]

BMJ Open

# The REFORM study protocol: a cohort randomised controlled trial of a multifaceted podiatry intervention for the prevention of falls in older people

| | |
|---|---|
| Journal: | *BMJ Open* |
| Manuscript ID: | bmjopen-2014-006977 |
| Article Type: | Protocol |
| Date Submitted by the Author: | 22-Oct-2014 |
| Complete List of Authors: | Cockayne, Sarah; University of York, Department of Health Sciences<br>Adamson, Joy; University of York, Department of Health Sciences<br>Corbacho, Belen; University of York, Department of Health Sciences<br>Fairhurst, Caroline; University of York, Department of Health Sciences<br>Hewitt, Catherine; University of York, Department of Health Sciences<br>Hicks, Kate; University of York, Department of Health Sciences<br>Hull, Robin; Harrogate and District NHS Foundation Trust, Podiatry Services<br>Keenan, Anne-Maree; University of Leeds, Leeds Institute of Rheumatology and Musculoskeletal Medicine; NIHR Leeds Musculoskeletal Biomedical Research Unit, Leeds Teaching Hospitals NHS Trust<br>Lamb, Sallie; University of Oxford, Nuffield Department of Orthopaedics, Rheumatology and Musculoskeletal Sciences, Kadoorie Critical Care Research Centre, John Radcliffe Hospital<br>Loughrey, Lorraine; Leeds Teaching Hospitals NHS Trust, NIHR Leeds Musculoskeletal Biomedical Research Unit; University of York, Department of Health Sciences<br>McIntosh, Caroline; National University of Ireland, School of Health Sciences, Discipline of Podiatry<br>Menz, Hylton; LaTrobe University, Faculty of Health Sciences<br>Redmond, Anthony; University of Leeds, Leeds Institute of Rheumatology and Musculoskeletal Medicine; Leeds Teaching Hospitals NHS Trust, NIHR Leeds Musculoskeletal Biomedical Research Unit<br>Rodgers, Sara; University of York, Department of Health Sciences<br>Vernon, Wesley; Sheffield Teaching Hospitals NHS Foundation Trust, Podiatry<br>Watson, Jude; University of York, Department of Health Sciences<br>Torgerson, David; The University of York, |
| <b>Primary Subject Heading</b>: | Health services research |
| Secondary Subject Heading: | Health economics, Research methods, Qualitative research, Geriatric medicine |
| Keywords: | HEALTH ECONOMICS, STATISTICS & RESEARCH METHODS, Musculoskeletal disorders < ORTHOPAEDIC & TRAUMA SURGERY, QUALITATIVE RESEARCH |

For peer review only - http://bmjopen.bmj.com/site/about/guidelines.xhtml

For peer review only

# The REFORM study protocol: a cohort randomised controlled trial of a multifaceted podiatry intervention for the prevention of falls in older people

Sarah Cockayne[1§], Joy Adamson[1], Belen Corbacho Martin[1], Caroline Fairhurst[1], Catherine Hewitt[1], Kate Hicks[1], Robin Hull[2], Anne-Maree Kenan[3,4], Sarah E Lamb[5], Lorraine Loughrey[1,3], Caroline McIntosh[6], Hylton B Menz[7], Anthony C Redmond[3, 4], Sara Rodgers[1], Wesley Vernon[8], Judith Watson[1], David Torgerson[1] on behalf of the REFORM study

[1]York Trials Unit, Department of Health Sciences, University of York, YO10 5DD, UK

[2] Podiatry Services, Harrogate and District NHS Foundation Trust, Harrogate District Hospital, Lancaster Park Road, UK

[3]NIHR Leeds Musculoskeletal Biomedical Research Unit, Chapel Allerton Hospital, Leeds, UK

[4]Leeds Institute of Rheumatology and Musculoskeletal Medicine, University of Leeds

[5]Nuffield Department of Orthopaedics, Rheumatology and Musculoskeletal Sciences, Kadoorie Critical Care Research Centre, John Radcliffe Hospital, University of Oxford, UK

[6]National University of Ireland, Galway, Republic of Ireland

[7]Lower Extremity and Gait Studies Program, Faculty of Health Sciences, La Trobe University, Bundoora 3086, Victoria, Australia

[8]Podiatry Services, Sheffield Teaching Hospitals NHS Foundation Trust, Jordanthorpe Health Centre, 1 Dyche Close, Sheffield, UK

[§]Corresponding author

Email addresses:



For peer review only - http://bmjopen.bmj.com/site/about/guidelines.xhtml

[§]SC: sarah.cockayne@york.ac.uk telephone number 01904 321736

JA: joy.adamson@york.ac.uk

BC: belen.corbacho@york.ac.uk

CF: caroline.fairhurst@york.ac.uk

CH: catherine.hewitt@york.ac.uk

KH: kate.hicks@york.ac.uk

RH: Robin.Hull@hdft.nhs.uk

AMK: a.keenan@leeds.ac.uk

SEL: sarah.lamb@ndorms.ox.ac.uk

LL: L.R.Loughrey@leeds.ac.uk

CM: caroline.mcintosh@nuigalway.ie

HBM: h.menz@latrobe.edu.au

ACR: a.redmond@leeds.ac.uk

SR: sara.rodgers@york.ac.uk

WV: wesley.vernon@nhs.net

JW: jude.watson@york.ac.uk

DT: david.torgerson@york.ac.uk

**Key words**

Cohort randomised controlled trial, multifaceted podiatry intervention, prevention of falls.

Word count 6919

For peer review only

## ABSTRACT

### Introduction

Falls and fall related injuries are a serious cause of morbidity and cost to society. Foot problems and inappropriate footwear may increase the risk of falls; therefore podiatric interventions may play a role in reducing falls. Two Cochrane systematic reviews identified only one study of a podiatry intervention aimed to reduce falls, which was undertaken in Australia. The REFORM trial aims to evaluate the clinical and cost-effectiveness of a multifaceted podiatry intervention in reducing falls in people aged 65 years and over in a UK and Irish setting.

### Methods and analysis

This multicentre, cohort randomised controlled trial will recruit 2,600 participants from routine podiatry clinics in the UK and Ireland to the REFORM cohort. In order to detect a 10% point reduction in falls from 50% to 40%, with 80% power 890 participants will be randomised to receive routine podiatry care and a falls prevention leaflet or routine podiatry care, a falls prevention leaflet and a multifaceted podiatry intervention. The primary outcome is rate of falls (falls/person/time) over 12 months assessed by patient self-report falls diary. Secondary self-report outcome measures include: the proportion of single and multiple fallers and time to first fall over a 12 month period; Short Falls Efficacy Scale – International; fear of falling in the past four weeks; Frenchay Activities Index; fracture rate; Geriatric Depression Scale; EQ-5D 3-L; health service utilisation at 6 and 12 months. A qualitative study will examine the acceptability of the package of care to participants and podiatrists.

### Ethics and dissemination




For peer review only - http://bmjopen.bmj.com/site/about/guidelines.xhtml

The trial has received a favourable opinion from the East of England - Cambridge East Research Ethics Committee and Galway Research Ethics Committee. The trial results will be published in peer-reviewed journals and at conference presentations.

**Trial registration number** Current Controlled Trials ISRCTN68240461

**Strengths and limitations of this study**

- This study is the first UK study evaluating the clinical and cost-effectiveness of podiatric care combined with footwear advice and provision (if required), orthotic inserts and foot and ankle exercises for falls prevention in people over 65 years of age.
- This study uses the novel cohort randomised controlled trial design.
- The study uses an unblinded, patient self-report primary outcome measure.
- It will not be possible to determine the clinical and cost-effectiveness of this intervention in patients who fall but do not receive routine podiatry care.



For peer review only - http://bmjopen.bmj.com/site/about/guidelines.xhtml

## BACKGROUND

Falls and fall related injuries are a serious cause of morbidity and cost to society [1], a burden which will increase with an ageing population. The National Service Framework (NSF) for Older People [2] recognises the importance of fall related injuries, and calls for health improvement plans to be devised that will reduce this burden.

It is well recognised that falls result from interactions between environmental hazards, a variety of medical conditions and physiological risk factors [3]. Foot problems may also increase the risk of falls. Foot problems affect one in three community dwelling people over the age of 65 years [4] and are associated with reduced walking speed and difficulty in performing activities of daily living [5-7].

There have been two relevant Cochrane reviews on falls prevention, one relating to falls in community dwelling older people [8] and one focusing on falls in hospitals and aged care facilities [9]. At the time of designing the study neither identified any randomised controlled trials (RCTs) focusing on podiatry-related interventions. A subsequent update has identified one trial of a podiatry based intervention for the prevention of falls [10] which related to the Australian health care system

There is some evidence to suggest that foot problems are associated with an increased risk of falling. Menz *et al's* [6] prospective study of 176 older people identified ankle flexibility, toe plantarflexor strength and plantar sensation as significant and independent predictors of balance and functional test performance. These factors were later confirmed as predictors of falling during a 12 month follow up of that cohort, in which foot pain was also identified as a



For peer review only - http://bmjopen.bmj.com/site/about/guidelines.xhtml

For peer review only

predictor of falling [11]. Mickle *et al's* [12, 13] prospective study of 312 people over 60 years of age also found that fallers had significantly higher prevalence of foot pain, and displayed significantly less strength of the hallux and were more likely to have hallux valgus and lesser toe deformities.

Inappropriate footwear may also impair balance and increase the risk of falling. Footwear characteristics considered detrimental to balance include high heels, soft soles and inadequate slip resistance [14, 15]. Prospective studies have shown that walking barefoot or wearing only stockings inside the home and wearing shoes with an increased heel height and smaller contract area increases the risk of falling [16-18]. People who have a history of falling are at increased risk of falls.

Given the emerging evidence that foot problems and inappropriate footwear increase the risk of falling, it has been suggested that podiatry may have a role to play in falls prevention, with several guidelines recommending that older people have their feet and footwear examined by a podiatrist [19, 20].

Several studies have also suggested that some treatments provided by podiatrists, such as lesion debridement [21], foot orthoses [22], foot and ankle exercises [23, 24] and footwear advice may play a role in improving balance. Combining these therapies should allow for stability and improved function to be achieved at each level. Lesion debridement can improve function during gait if pain is reduced, exercise programmes focus on internal strengthening and flexibility, and appropriate footwear with orthotic devices can provide external support, improved kinaesthesia and improved function. A trial conducted in Australia in 305 community dwelling older people with disabling foot pain showed a 36%


For peer review only - http://bmjopen.bmj.com/site/about/guidelines.xhtml

reduction in falls for those receiving a multifaceted podiatry intervention, consisting of a foot orthosis, advice on footwear, subsidy for new footwear, a home based programme of foot and ankle exercises, a falls prevention education booklet and routine podiatry care for 12 months [10, 25]. The control group received only routine podiatry care for 12 months which consisted of toenail maintenance and scalpel debridement of hyperkeratotic lesions (corns and calluses). There have been no further large pragmatic trials looking at podiatric care combined with footwear advice, foot and ankle exercise and orthotic inserts for falls prevention, and there has been no assessment of whether such an intervention is economically viable within the UK health care setting. The REFORM (REducing Falls with ORthoses and a Multifaceted podiatry intervention) trial aims to address these issues. The main objectives are to investigate the clinical and cost-effectiveness of a multifaceted podiatry intervention for falls prevention and to assess views and experiences of the intervention and the trial process from the perspective of the participants and podiatrists.

## METHODS AND ANALYSIS

### Design

The REFORM study is a "cohort randomised controlled trial" (cRCT). Firstly we will recruit participants to the REFORM cohort and whilst this is being assembled, we will invite a selection of eligible participants from the site undertaking the pilot phase to take part in a REFORM pilot study. Once the pilot study is complete, we will then invite the remaining eligible participants to take part in the main REFORM trial. The REFORM main trial is a two arm, pragmatic, open, multicentre, randomised controlled trial with a 12 month follow-up.


For peer review only - http://bmjopen.bmj.com/site/about/guidelines.xhtml
For peer review only

**REFORM study objectives for the pilot phase**

(i) To demonstrate the feasibility of recruiting to the REFORM cohort

(ii) To develop and pilot the multifaceted podiatry intervention including a foot and ankle exercise programme, supplementary DVD and booklet with approximately 60 participants

(iii) To develop the podiatrist training package

(iv) To pilot the falls calendar and other patient data collection questionnaires

(v) To assess participants' views and experiences of the intervention and the trial process

(vi) Pilot, review and refine if necessary recruitment methodology for the main trial

Upon successful achievement of these objectives, we will seamlessly progress to the main REFORM trial.

**REFORM study objectives for the main study**

This study will aim to address the following:

(i) To train the podiatrists to deliver the intervention

(ii) To examine the clinical and cost-effectiveness of the multifaceted podiatry intervention for falls prevention

(iii) To assess the podiatrists' views and experiences of the intervention and trial process

**Participants**

Participant recruitment

Participants will be recruited from NHS podiatry clinics based in either primary or secondary care in the UK and one international site in the Republic of Ireland. The rationale for



For peer review only - http://bmjopen.bmj.com/site/about/guidelines.xhtml

recruiting only from podiatry clinics is due to the logistical constraints imposed by the requirement for the control group to receive routine podiatry care. Whilst it would be possible to recruit from GP practices, many of the patients identified in this setting would not be receiving routine podiatry care, and NHS podiatry service managers indicted that they would not have the capacity to see large numbers of additional patients.

Podiatrists within the NHS Trust or clinic at the Republic of Ireland or the REFORM research podiatrist will undertake a search of either electronic or paper patient medical notes to identify potential participants for the study. Patients aged 65 years and over who are registered with the service and have attended routine podiatry services within the past six months of the search being undertaken will be identified and will be eligible for an invitation mailing. Patients who have attended high risk clinics, (for example diabetes clinics), or who live in a nursing home will be excluded from the invitation mail out. Sites will be requested where possible to screen out patients in the following groups: patients with a life expectancy of less than six months; patients known to have dementia, a neurodegenerative disorder, neuropathy, a lower limb amputation, or are chair or bed bound. All eligible patients will be sent an invitation pack (letter of invitation, participant information sheet, consent form, screening questionnaire and pre-paid envelope) asking whether they would like to participate in the REFORM study. Where the clinic has the capacity, potential participants attending routine podiatry clinics may be approached opportunistically. Where a multidisciplinary team exists, other healthcare professionals such as occupational therapists, falls practitioners and physiotherapists may support the opportunistic recruitment.

Participants wishing to take part in the REFORM study will be asked to return their completed consent form and screening questionnaire by post to the York Trials Unit (YTU).



For peer review only - http://bmjopen.bmj.com/site/about/guidelines.xhtml

Researchers at the YTU will assess the returned screening forms for participant eligibility. During the consenting stage, potential participants will be informed of the possibility of participating in other related studies, e.g. an associated qualitative study and they will be asked to indicate (by ticking a box on the consent form), if they would prefer not to be approached about these studies. Participants can withdraw from the study at any point. The reason for withdrawal will not have to be declared; however, if provided, this will be recorded. Data will be retained for all participants up to the date of withdrawal, unless a participant specifically requests their details be removed.

**Inclusion criteria for the REFORM cohort**

All eligible, consenting participants will be asked to complete a baseline questionnaire. Participants who return valid baseline data will be included in the REFORM cohort. Any participant reporting a score of 10 or more on the Geriatric Depression Scale [26, 27] i.e. more severe depression, will be referred to their general practitioner (GP).

**Exclusion criteria for the REFORM cohort**

Participants will be ineligible for the REFORM cohort if they are under 65 years of age; report having neuropathy, dementia or other neurological condition such as Parkinson's disease, Alzheimer's, multiple sclerosis, Lou Gehrig's/amyotrophic lateral sclerosis or Huntington's disease; are unable to walk household distances (10 metres) without the help of a walk aid such as a Zimmer frame or walker or person to assist; have had a lower limb amputation; or are unwilling to attend their podiatry clinic.

**Inclusion criteria for the REFORM trial**

Participants will be eligible for the REFORM trial if they:



For peer review only - http://bmjopen.bmj.com/site/about/guidelines.xhtml

(i) have had one fall in the past 12 months; or one fall in the past 24 months requiring hospital attention; or report a fear of falling on their baseline questionnaire

i.e. have worried about falling at least some of the time, in the past four weeks

(ii) are community dwelling

**Exclusion criteria for the REFORM Trial**

Participants who do not complete the baseline or run-in data collection instruments adequately, or who are unable to read or speak English will be excluded from the trial**.**

**Participants who do not wish to take part in the study**

Participants who do not wish to take part in the main study are not required to return any forms to the YTU. However, all participants in the pilot phase of the study who are sent an invitation pack will be given the opportunity to decline participation and if willing, provide some demographic information and reason for declining. This will provide us with sufficient information to document the reasons why participants do not wish to take part in the study and will allow us to compare decliners to those who are participating. The recruitment pack in the main mail out will not contain a decline form.

**Sample size**

The REFORM cohort

We propose to recruit up to 2,600 participants into the REFORM cohort to allow us to sample for the pilot trial and to allow cohort attrition before we sample for the main REFORM trial.

Internal pilot phase



For peer review only - http://bmjopen.bmj.com/site/about/guidelines.xhtml

Pilot trial: In order to pilot the intervention, a random sample of 60 participants will be selected from the REFORM cohort. Participants will be allocated equally to each of the two groups i.e. 30 participants per group. The allocation sequence will be computer generated by the YTU randomisation service and will be stratified by centre.

REFORM main trial

The REFORM trial is designed to detect a 10% point reduction in the number of people who fall over a 12 month period. Assuming this high risk group has an underlying risk of falling in a 12 month period of 50% [28] then in order to observe a reduction to 40% with 80% power and a two-sided 5% significance level we would require 890 participants (445 in each group, allowing for a 10% loss to follow up).

**Randomisation**

Participants who fulfil the eligibility criteria for the REFORM trial and who have provided written consent to take part in the study will be eligible for randomisation. Randomisation will be carried out by the YTU secure remote computer randomisation service. Eight hundred and ninety participants will be randomly allocated in a 1:1 ratio to either the intervention or control group. If more than 890 eligible participants are recruited then we will randomly allocate the remaining participants in a 2:1 ratio in favour of the control group. This will allow us to increase the power of the study, without putting patients at any additional risk and without incurring additional costs. In order to allow for manageable case-loads at individual sites, the randomisation is by permuted blocks and stratified by centre. The YTU will write to the participant’s GP informing them of study participation and to participants who are allocated to the intervention group.



For peer review only - http://bmjopen.bmj.com/site/about/guidelines.xhtml

**Blinding**

Due to the nature of the intervention, blinding of participants will not be feasible. Blinding of members of the study team who are actively involved in the administration of the study and may collect primary outcome falls data or undertake data queries on secondary outcomes, or the health economist will not be possible. Members of the study team responsible for data entry and the statistical analysis of the study will be kept blind to group allocation.

**Intervention group**

Participants allocated to receive the intervention will be seen by the podiatrist at the clinic they would normally attend for routine podiatry care, as soon as possible after randomisation. Participants are invited to attend two podiatry visits approximately two to four weeks apart, with further appointments if required in addition to their usual podiatry care.

(i) Footwear advice and provision

Participants' everyday footwear (indoor and outdoor footwear) will be assessed according to the following characteristics: appropriate size; method of fastening; height and width of the shoe's heel; thickness of outsole; heel counter stiffness; longitudinal sole rigidity; sole flexion point and tread pattern. Footwear will be deemed to be inappropriate if the shoes have any of the following characteristics: (i) heel height is greater than 4.5cm; (ii) no adjustable fixation of the upper; (iii) no heel counter or the heel counter can be depressed to greater than 45°; (iv) a fully worn/smooth/thin sole; (v) the shoe heel width is narrower than the participant's heel width by greater than or equal to 20%; or (vi) incorrect shoe size [10]. Participants with inappropriate footwear will be counselled regarding the hazardous footwear feature(s) identified during the assessment and will be advised on safer characteristics for future



For peer review only - http://bmjopen.bmj.com/site/about/guidelines.xhtml

footwear purchases. Where possible, footwear will be provided for participants whose footwear is deemed to be inappropriate. The podiatrist will order shoes directly from two companies participating in the Healthy Footwear Guide (HFG) scheme [29]; DB shoes (Rushden, UK) or Hotter company (Skelmersdale, UK). As not all of the shoes manufactured by these companies fulfil the characteristics of a safe shoe, the trial team will assess the shoes and provide a list of suitable footwear from which participants can choose. In order to avoid incentivising patients to take part in the study, participants will only be told about footwear provision, if they are deemed to need new footwear. During the REFORM pilot trial we will explore the feasibility of making a footwear assessment, providing footwear advice and provision of footwear and if necessary we will revise the intervention if needed.

(ii) Foot orthoses

We originally planned to use the same orthotic device (Formthotics™, Foot Science International, Christchurch, NZ) (Figure 1) as our Australian collaborator [10, 25] as they had demonstrated that this device, commonly used in Australia, was acceptable to patients and was shown to be associated with a reduction in falls. However, during the setup period of the pilot phase of the REFORM trial, podiatrists at the recruiting sites reported difficulties when fitting these devices with the addition of posting (the application of wedges under the forefoot or rearfoot) and the ease of fitting in patient's current shoes. These sites frequently use the x-line® orthoses range (Healthystep, Mossley, UK) (Figure 2) as they are easy to modify and are easy to fit in patient's current shoes. During the pilot trial we will provide participants with both types of device to determine which device is most acceptable to a UK population. The Formthotics™ device will be issued as per the manufacturing guideline using the appropriate heating equipment and fitting procedure. The x-line® PressurePerfect/Standard device will be fitted and if indicated, modified to improve foot posture as assessed in usual



For peer review only - http://bmjopen.bmj.com/site/about/guidelines.xhtml

clinical practice by the individual clinician. If a participant already has insoles, whether shop bought or prescribed, the clinician will make a clinical judgement on the suitability of replacing their current insole with the trial insole. If the current insole is replaced with the trial insole then any current prescription/modifications maybe repeated, if applicable. We would consider the insole component of the intervention already addressed if the clinician felt it detrimental to replace their current insole with that of the trial insole. If the podiatrist feels that the patient requires more than what the current or trial insole can provide, or if they present with a musculoskeletal pathology, then a referral will be made in line with routine practice.

(iii) Home-based foot and ankle exercise programme

Participants will be prescribed a 30 minute home-based foot and ankle exercise programme which will be undertaken three times per week, indefinitely. The exercise programme is aimed at stretching and strengthening the muscles of the foot and ankle. The exercises will be based on the programme developed by Spink *et al* [10, 25] and will be adapted to make it suitable for a UK setting during the pilot phase of the trial. A summary of the individual exercises is given in Table 1. The exercises will be demonstrated by the podiatrist and will be supplemented by a DVD and an illustrated explanatory booklet. Participants will be assessed for competence and safety at the baseline and follow-up appointments.

(iv) Routine podiatry care

Routine podiatry care will continue to be given at separate podiatry appointments in accordance with participant's usual care. This will aim to reduce painful conditions such as corns and callouses, that have been found to be associated with an increased risk of falls.



For peer review only - http://bmjopen.bmj.com/site/about/guidelines.xhtml

**Table 1 Summary of the home based foot and ankle exercises**

| Activity | Description | Dosage | Increments |
|---|---|---|---|
| Ankle range of motion/ warm up | Sitting with the knee at 90 degrees. Lift the foot to clear the ground and then rotate the foot slowly in a clockwise direction and then an anti-clockwise. | 1x10 repetitions for each foot in each direction. | None. |
| Ankle inversion strength | Sitting upright, hip, knee and ankle at 90º. Invert foot against resistive exercise band. The band should be fixed at 90 degrees to the foot from an additional chair/table leg. | 3x10 repetitions for each foot. | Increase resistance strength of resistive exercise band. |
| Ankle eversion strength | Sitting upright, hip, knee and ankle at 90º. Evert foot against resistive exercise band The band should be fixed at 90 degrees to the foot from an additional chair/table leg | 3x10 repetitions for each foot. | Increase resistance strength of resistive exercise band. |
| Ankle dorsiflexion strength | Sitting, hip, knee and ankle at 90º.<br>Dorsiflex both feet to end range of motion and hold. Keep pulling feet up towards the body during the hold. | Hold feet in dorsiflexion for 3x10 seconds. | Increase repetitions up to maximum of 10. |
| Intrinsic strengthening, toe plantarflexion strength and toe stretch | Sitting, hip, knee and ankle at 90 º.<br>1) Use the therapy ball under the toes to stretch the toes. The rest of the foot should be plantargrade. Then curl and point the toes up and over the ball.<br>2) Use the therapy ball under the toes to stretch the toes. The rest of the foot should be plantargrade. With the heel on/close to the floor, curl the toes over the ball and attempt to pick up the ball with the toes. | 3 x10 repetitions for each exercise both feet. Have a 30 sec break between each repetition | Increase up to a maximum of 50 repetitions. |
| Ankle plantarflexion strength | From standing position, rise up onto toes of both feet and then slowly lower back down. | 3x10 repetitions. | Increase repetitions up to maximum of 50. |

For peer review only



For peer review only - http://bmjopen.bmj.com/site/about/guidelines.xhtml

| | | | |
|---|---|---|---|
| | Just before the heels contact the floor, rise back up onto the toes. | | |
| Calf stretch | Facing a wall and using hands on the wall for balance, step one foot in front of the other keeping feet hip width apart and hips, knees and feet facing the wall. Bend the knee closest to the wall and keep the back leg straight. Keep both heels in contact with the floor. | Hold stretch for 3x20 seconds on each leg. | Increase the stride length and forward lean to increase the stretch |
| Proprioception/balance training | From a standing position and holding on to a work surface/chair/wall for support, stand on one leg. Repeat on the other side | Hold for 30 seconds. Repeat 3 repetitions. | Increase slowly to hold for 1 min per repetition. If competent, rise up on to toes on the one supporting leg: 3 x 10 repetitions. |

(vi) Falls prevention leaflet and trial newsletter

Participants will receive a copy of the latest falls prevention advice leaflet produced by Age UK (the current version 'Staying steady' was printed in June 2010). This leaflet will be sent to the participant in the post with their baseline questionnaire.

Participants will be sent a site and group specific newsletter at three and 12 months informing them about progress of the study. The intervention group's newsletter will include a section about the foot and ankle exercises in order to aid compliance. All participants will receive £5 in recognition of their participation and to offset any incidental expenses associated with completing the questionnaires.



For peer review only - http://bmjopen.bmj.com/site/about/guidelines.xhtml

**Control group**

Participants allocated to the control group will receive the same falls prevention advice leaflet produced by Age UK. This leaflet will be sent to the participant in the post with their baseline questionnaire. Participants will continue to receive routine podiatry and GP access in accordance with their usual care. Participants will also be sent a site and group-specific newsletter at three and 12 months. All participants will receive £5 in recognition of their participation and to offset any incidental expenses associated with completing the questionnaires.

**Primary outcome measure for the REFORM trial**

The primary outcome is the rate of falls (i.e. falls/person/time) where a fall is defined as "an unexpected event in which the participant comes to rest on the ground, floor, or lower level" [30]. Data will be collected via participant self-reported monthly falls calendars throughout the 12 months following randomisation. Participants will be asked to record each day whether or not they had any fall. Participants who do not return their falls calendar within one week of the due date will be telephoned by the YTU to obtain the missing data. Participants will also be given a Freephone number to report any falls as soon as possible after the fall. Information collected includes: date and location of fall; reason for fall; any injuries sustained (e.g. a superficial wound or a broken bone); hospital admissions; footwear worn at the time of the fall; and if the patient was wearing an orthotic or using a walking aid. As we are collecting falls data at six and 12 months follow up, these data will be used for those participants who do not return their monthly falls calendar.

For peer review only



For peer review only - http://bmjopen.bmj.com/site/about/guidelines.xhtml

**REFORM trial secondary outcome measures**

All secondary outcomes are self-reported by the patient and collected by questionnaire at baseline, six and 12 months, or by monthly falls calendars. Secondary outcomes include: proportion of fallers (single and multiple); patient reported time to first fall during follow-up; health related quality of life as measured by the EQ-5D [30]; fear of falling as measured by the question, "During the past 4 weeks have you worried about having a fall?", fear of falling as measured by the Short Falls Efficacy Scale – International (FES-I) [31], activities of daily living as measured by the Frenchay Activities Index (FAI) [32], fracture rate, health service utilisation and depression as measured by the short form Geriatric Depression Scale (GDS) [27].

**Nested qualitative study**

A qualitative evaluation will be carried out to examine the acceptability of the intervention as a whole package of care, to both the trial participants and the podiatry practitioners. Trial participants from the pilot phase of the study who are receiving the intervention will be asked about their experience of the intervention. Particular attention will be paid to the acceptability and compliance with the foot orthosis, exercise programme, podiatry service and footwear advice/purchase and how the intervention fits into the individual's wider experience of balance problems within their everyday lives.

Ten to 15 participants will be purposively selected from the pilot phase of the trial (the first 60 participants randomised) to ensure a representative spread according to falls history, age and gender. This maximum variation sampling approach [33] will ensure a wide range of viewpoints are included in the data collection and analysis. Participants will be invited to attend a face-to-face interview or, if prefered, a telephone interview. Written informed



For peer review only - http://bmjopen.bmj.com/site/about/guidelines.xhtml

For peer review only

consent will be obtained from the participant prior to the interview. The semi-structured interviews will be conducted within approximately two weeks of receiving the six month follow-up questionnnaire. A follow-up telephone interview will be conducted within two weeks of receiving the 12 month follow-up questionnaire to discuss any changes (or not) recorded in the outcomes measured over the longer term follow-up period. All interviews will be conducted using a topic guide to ensure consistency across participants. However, the format will be flexible in order to allow participant-led data regarding what they constitute as important and/or successful in terms of treatment outcome.

We will interview a purposive sample of five to ten practitioners who are providing the podiatry services for the study regarding the way in which they have delivered the intervention, the ease of delivery, their confidence in the intervention, and their experience of being involved in the trial. Face-to-face or telephone interviews will be conducted approximately three months into the trial process. Informed consent will be obtained prior to the interview being conducted.

**Adverse events**

Details of any adverse events reported to the YTU either directly by the participant or by a member of the research team at the recruiting site will be recorded.

This study will report details of any serious adverse events (SAEs) that are required to be reported to the Research Ethics Committee (REC) under the current terms of the Standard Operating Procedures for RECs.

A SAE is defined as any untoward occurrence that:



For peer review only - http://bmjopen.bmj.com/site/about/guidelines.xhtml

For peer review only

(i) Results in death;

(ii) Is life threatening;

(iii) Requires hospitalisation or prolongation of existing hospitalisation;

(iv) Results in persistent or significant disability or incapacity;

(v) Consists of a congenital anomaly or birth defect, or

(vi) Is otherwise considered medically significant by the investigator.

An event is defined as 'related' if the event was due to the administration of any research procedure. An 'unexpected event' is defined as a type of event not listed in the protocol as an expected occurrence. Expected events include: aches and pains in the lower limb lasting for longer than 48 hours; fall; new callus/corn formation, blisters, ulcers; skin irritation/injury including pressure sores and soft tissue injury.

In the context of this study, an occurrence of the type listed in (i) to (vi) will be reported as an SAE only if:

(i)

the event is suspected to be related to an aspect of the research procedures (e.g. wearing the orthotic, undertaking the exercise programme, completion of follow-up questionnaires, participation in feasibility or qualitative sub-studies, telephone contact), and;

(ii)

it is an unexpected occurrence. Hospitalisations, disabling / incapacitating / life-threatening conditions, falls and deaths are expected in the study population due to the age of the cohort, they will therefore only be reported as SAEs if they appear to be related to an aspect of taking part in the study.



For peer review only - http://bmjopen.bmj.com/site/about/guidelines.xhtml

## Other data collected

Treatment details will be recorded by the podiatrist including: the number of podiatry visits; an eligibility checklist with details on relevant health conditions and test results; characteristics of current indoor and outdoor shoes; details relating to shoes ordered; details on the type and prescription of any current insole use; the type of insole issued/retained with any modifications made, and any amendments or advice given on the intervention due to safety reasons.

Information on adherence to the exercise, footwear advice and orthotic components of the intervention will be collected from participant self-reported questionnaire data at three, six and 12 months. Participants will be asked whether during the past month, they were wearing their orthotic 'all of the time', 'most of the time', 'some of the time', 'a little of the time' or 'none of the time'. Participants will also be asked, for the past month, typically how many times a week they did the exercises: none, one, two, three, or more than three times. Participants will be asked at 12 months if they were given footwear advice, and whether or not they followed any given advice.

## Statistical analysis

There will be a single analysis at the end of the trial, conducted using STATA (StataCorp, 4905 Lakeway Drive, College Station, Texas 77845 USA). All analyses will be conducted on an intention to treat basis, i.e. including all randomised patients in the groups to which they were assigned. All tests will be two-sided at the 5% significance level. If more than 890 participants are recruited and unequal allocation is utilised, then analyses will be adjusted to take this into account.



For peer review only - http://bmjopen.bmj.com/site/about/guidelines.xhtml

For peer review only

(i) The REFORM cohort

Descriptive statistics will be presented for participants in the REFORM cohort.

(ii) REFORM trial

Participant baseline data will be summarised descriptively by randomised arm. No formal statistical comparisons will be undertaken. Continuous measures will be reported as means and standard deviations whilst the categorical data will be reported as counts and percentages.

(iii) Statistical analysis of the REFORM trial primary outcome

The number of falls per person will be analysed using a Poisson regression model adjusting for gender, age, centre and history of falling to estimate the difference in falls rate between the groups. If there is over dispersion, a negative binomial regression model adjusting for the same factors will be used [34]. Point estimates and their associated 95% confidence intervals will be provided.

(iv) Statistical analysis of the REFORM trial secondary outcomes

The proportion of fallers versus non-fallers in each group will be compared by logistic regression adjusting for gender, age, centre and history of falling. Odds ratios and their associated 95% confidence intervals will be provided. The proportion of multiple fallers versus single or non-fallers in each group will be compared over the 12 month trial period using logistic regression adjusting for gender, age, centre and history of falling.

The time to the first fall will be derived as the number of days from randomisation until the patient reports having a fall as detailed from the participant's falls calendar, falls telephone



For peer review only - http://bmjopen.bmj.com/site/about/guidelines.xhtml

data collection sheet or self-reported questionnaire. Participants who have not had a fall will be treated as censored at their date of trial exit, or date of last available assessment or 365 days/trial cessation, as appropriate. The proportion of patients yet to experience a fall will be summarised by a Kaplan Meier survival curve for each group. The time to first fall will be analysed by Cox Proportional Hazard regression adjusting for gender, age, centre and history of falling. Hazard ratios and their associated 95% confidence intervals will be provided. The proportional hazard assumption will be evaluated using Schoenfeld residuals. The median time to the first fall and its associated 95% confidence intervals will be estimated from this adjusted model.

Patients with a score of six or more on the short form Geriatric Depression Scale will be categorised as having depression. The proportion of people with depression in each group will be compared over the 12 month trial period using logistic regression adjusting for gender, age, centre and history of falling.

The following secondary outcomes: Short Falls Efficacy Scale - International, fear of falling in the past four weeks, and the Frenchay Activities Index will be treated as continuous data and will be measured at baseline, month six and month 12. A covariance pattern model incorporating all post randomisation time points will be used to compare the two groups on these outcomes adjusting for baseline score, gender, age, centre and history of falling. The correlation of observations within patients over time will be modelled and the model effects and their associated 95% confidence intervals will be provided.

The proportion of patients obtaining at least one fracture over the 12 month follow-up period will be compared by logistic regression adjusting for gender, age, centre and history of



For peer review only - http://bmjopen.bmj.com/site/about/guidelines.xhtml

For peer review only

falling. Odds ratios and their associated 95% confidence intervals will be provided. If there are patients that obtain multiple fractures in this time (from different events), then the proportion of patients obtaining multiple fractures versus single or no fractures in each group will be compared over the 12 month trial period using logistic regression adjusting for gender, age, centre and history of falling.

(v) Intervention adherence

Descriptive statistics will be used to summarise the adherence to the exercise and orthotic at three, six, and 12 months and adherence to following footwear advice. A Complier Average Causal Effect (CACE) analysis to assess the impact of compliance on treatment estimates will be considered.

(vi) Missing data

The amount of missing data will be reported for each randomised arm.

(vii) Qualitative analysis

All interviews will be audio recorded digitally and transcribed verbatim. The computer package ATLAS-ti will be used to manage the data. Data will be analysed according to the constant comparison method through thematic coding of the data [35]. Coding will take place using a combination of *a priori* themes (according to the aims of the qualitative study and the outcome measures of interest to the trial) and emergent themes. However, in addition, as participants in the qualitative sample would also have responses to the quantitative data collected for the trial, this will allow for the possibility of taking a mixed-methods approach to data integration, in which the two forms of data can be used in a complementary way [36].



For peer review only - http://bmjopen.bmj.com/site/about/guidelines.xhtml

For peer review only

(viii) Adverse event data

Adverse event data will be summarised descriptively by randomised arm.

**Economic evaluation**

The economic analysis will be performed using individual patient level data from the REFORM trial. The analytical approach will take the form of cost-effectiveness and cost-utility analyses. The cost-effectiveness approach will assess value for money in terms of cost per fall averted, and the cost-utility analysis will assess cost per quality adjusted life year (QALY) gained. The perspective for both analyses will be that of the UK NHS and Personal Social Services [37] as well as that of society. Discounting for future cost and health benefit will not be included considering the time frame for the trial is 12 months after randomisation. The year of pricing will be set as the mid-year of the trial.

Health benefits associated with the treatments will be measured in terms of both estimates of the mean number of falls, corresponding to the main outcome of the trial, and mean QALYs, which is defined as a year lived with full health. The EQ-5D [38] will be used to elicit patient utility values at different points in time and used to calculate QALYs for each patient using the area under the curve [39]. These utility values are used as ‘quality adjustment’ for each patient’s survival time.

Mean within-trial estimates of cost and health benefits will be estimated using the regression approach to allow for the correlation between costs and effects as well as adjusting for covariates. This analysis will also account for skewness and censoring associated with time to event and cost data [40-42]. The result will be presented as incremental cost-effectiveness ratio (ICER) where the difference of mean cost estimates between two arms are divided by



For peer review only - http://bmjopen.bmj.com/site/about/guidelines.xhtml

the difference of mean health benefit between two arms. The Net Monetary Benefit (NMB) will be also presented [43]. The NMB provides an estimation of the gain (or loss) in resources of investing in a particular intervention when those resources might be used elsewhere [44].

The uncertainty surrounding the decision to accept a treatment as the most cost-effective will be explored in cost-effectiveness acceptability curves (CEAC) [45]. These curves depict the probability of accepting a treatment as being cost-effective for a large range of willingness to pay values for an extra unit of health benefit. Sensitivity analysis will be conducted to explore the impact of underlying assumptions of the model and the range of unit costs on the cost-effectiveness results.

Despite the careful design of any trial the presence of missing data is unavoidable. The reasons for the presence of incomplete data are multiple. Similarly there are different methods to handle its analysis [46]. The pattern of missing data for the economic analysis will be examined and described. The Multiple Imputation (MI) process has been recommended as the appropriate method to address the uncertainty in the results of economic evaluation due to missing data. Therefore the base case analysis will be based on the MI, whilst the complete case dataset will be used for the sensitivity analysis.

The main outcome of the REFORM trial, fall reduction, could be regarded as an intermediate outcome to achieve the final target - the reduction in fracture. However, due to the restriction in the length of follow-up, the long-term effect of decreasing the number of fractures, might not be observed within the timeframe of the current trial. Therefore a further analysis is

For peer review only


For peer review only - http://bmjopen.bmj.com/site/about/guidelines.xhtml

planned to model the possible long-term impact of the trial assuming that a falls reduction should also lead to a fracture reduction.

A decision analytic model approach will be adopted to perform such a task. The perspective will be the UK NHS and Personal Social Services and the time horizon for this analysis will be a life-time horizon. Life-time horizon refers to following up every single participant in a hypothetical cohort until the last participant dies. The hypothetical cohort will be constructed, based on the characteristics of the trial population, to estimate the QALY yield and cost saving of the long-term effect of the intervention. The model parameters which are not collected in the trial will be extracted from the existing literature. The model outputs will be the estimated expected mean costs, effectiveness, and QALYs associated with each alternative treatment. Estimated total costs and outcomes will be discounted properly according to the current guidance of health technology appraisal [37].

Uncertainty regarding cost-effectiveness will be evaluated using probabilistic sensitivity analysis, where inputs into the analysis are defined as probability distributions which reflect uncertainty. The uncertainty surrounding the decision to adopt a given treatment option as a cost-effective treatment at different levels of willingness to pay will be represented in acceptability curves. The impact of assumptions undertaken in the analysis regarding the evidence over parameters or relating to the decision model (such as extrapolation) will be evaluated in a sensitivity analysis, if possible.



For peer review only - http://bmjopen.bmj.com/site/about/guidelines.xhtml

For peer review only

## ETHICS AND DISSEMINATION

### Ethics

The study has been approved by the NRES East of England - Cambridge East Research Ethics Committee and Galway Research Ethics Committee, Republic of Ireland. It has also been approved by the National Health Service (NHS) research and development (R&D) committee responsible for each participating trust as well as the University of York, Department of Health Sciences Research Governance Committee. All participants will give written informed consent prior to entry to the study.

### Dissemination

Results of the study will be disseminated through, national and international research conferences and in articles published in international peer-reviewed journals.

## DISCUSSION

This study uses the cohort randomised controlled trial (cRCT) design. Existing trial designs have reported issues around recruitment, ethics, patient preferences and treatment comparisons [47]. We chose this approach to find out whether the cRCT design can overcome these issues and to test its feasibility. We anticipate that using this design will offer the following advantages. First, we expect that recruitment rates will be enhanced. Some participants enrolled in the cohort will be immediately eligible for the main trial and can be randomised straight away. Others, however, will become eligible over time due to the fact that they have subsequently had a fall. If an alternative design had been chosen then these patients could not have been included in the study and would have been lost. Second, in this study participants are receiving routine podiatry care outside of the trial and apart from altruistic reasons the only incentive to take part in the study is the possibility of receiving the



For peer review only - http://bmjopen.bmj.com/site/about/guidelines.xhtml

extra package of podiatry care. If a pragmatic design had been used, then the control patients would have been notified of their group allocation, and this may have resulted in a higher attrition rate. If this had occurred then there is the possibility that selection bias could be introduced. In addition to this, participants may also either knowingly or unknowingly bias the trial by reporting the number of falls they have had less conscientiously than those allocated to the intervention arm. In the cRCT design, participants are only notified of their allocation to the intervention group. Control participants are only aware of their participation in the study as a whole, thereby minimising the possibility of introducing attrition and reporting bias. Third, we expect that using the cRCT design will help minimise post-randomisation attrition rates. Unfortunately, many trials lose participants, and unless this occurs in a random manner, there is the possibility of selection bias being introduced. Many participants are lost in the early stages of a study e.g. participants change their mind. Using the cRCT design, which effectively has a run-in period, allows the majority of patients to withdraw from the study prior to randomisation, thereby minimising post-randomisation attrition rates. Finally, one key feature of the cRCT design is the capacity to undertake multiple randomised controlled trials over time. Once this cohort is set up, we will apply for funding to undertake further trials using the participants in this study. This is not only a quick and cost-effective strategy to identifying trial participants but has the added advantage of reducing heterogeneity of trial populations enabling synthesis of trial data and indirect comparisons between trial treatments.

Falls in older people are a major health problem. Podiatry may have a role in preventing falls as there is some evidence to suggest that foot problems and inappropriate footwear may increase the risk of falling. A recent trial by Spink *et al* [10, 25] has demonstrated that a podiatric intervention consisting of: footwear assessment and provision; foot orthotic device



For peer review only - http://bmjopen.bmj.com/site/about/guidelines.xhtml

and a home-based foot and ankle exercise programme can reduce falls in patients aged over 65 years of age with disabling foot pain. As far as we are aware, such a multifaceted podiatry intervention has not been evaluated in a UK and Republic of Ireland setting. The REFORM trial aims to evaluate the clinical and cost-effectiveness of such a package of care in patients over the age of 65 years who have an increased risk of falling.

## TRIAL STATUS

Recruitment and follow up are in progress. Recruitment to the study began in October 2012 and will continue until approximately spring 2015. Patients will continue to be followed-up until approximately spring 2016.

## COMPETING INTERESTS

The authors declare that they have no competing interests.

## AUTHORS' CONTRIBUTIONS

DT and SC wrote the original protocol. SC, JA, CH, RH, AMK, SL, CM, HBM, ACR, WV JW and DT were applicants on the NIHR HTA funding application they and BCM, CF, KH, LL, SR have helped to further refine the protocol and have made substantial contributions to the design of the project. All authors read and approved the final manuscript. We would like to thank the Trial Steering/Data Monitoring and Ethics Committee for their input and advice with the study.

## ACKNOWLEDGEMENTS

This study is funded by the National Institute for Health Research (NIHR)



For peer review only - http://bmjopen.bmj.com/site/about/guidelines.xhtml

Health Technology Assessment (HTA) Programme (Programme grant number 09/77/01). The views and opinions expressed herein are those of the authors and do not necessarily reflect those of the Department of Health. Lamb was supported by the National Institute of Health Research (NIHR) CLAHRC, Oxford Health NHS Trust and the NIHR BRU, Nuffield Orthopaedic Centre, University of Oxford. HBM is currently a National Health and Medical Research Council of Australia Senior Research Fellow (ID: 1020925). The research team would like to thank the independent members of the Trial Steering/Data Monitoring and Ethics Committee Professor Roger Francis, Dr Sara Brookes, Dr Margaret May, Professor Chris Nester and Dr Ian Mathieson for their advice and for overseeing the study.

## REFERENCES

1. Iglesias CP, Manca A, Torgerson DJ. The health-related quality of life and cost implications of falls in elderly women. Osteoporos Int 2009;20(6):869-78 doi: 10.1007/s00198-008-0753-5 [published Online First: 10 October 2008].

2. Department of Health (DH) 2001. National Service Framework for Older People: London Department of Health. Available at: https://www.gov.uk/government/publications/quality-standards-for-care-services-for-older-people (accessed 20/10/2014)

3. Lord S, Sherrington C, Menz H, et al. Falls in older people: Risk factors and strategies for prevention. Second edition, Cambridge, UK:Cambridge University Press 2007.

4. Dunn JE, Link CL, Felson DT, et al. Prevalence of Foot and Ankle Conditions in a Multiethnic Community Sample of Older Adults. Am J Epidemiol 2004;159(5):491-498

5. Benvenuti F, Ferrucci L, Guralnik J, et al. Foot pain and disability in older persons: an epidemiologic survey. J Am Geriatr Soc. 1995;43(5):479-84.



For peer review only - http://bmjopen.bmj.com/site/about/guidelines.xhtml

6. Menz HB, Morris ME, Lord SR. Foot and Ankle Characteristics Associated With Impaired Balance and Functional Ability in Older People. J Gerontol A Biol Sci Med Sci. 2005 Dec;60(12):1546-1552

7. Barr EL, Browning C, Lord SR, et al. Foot and leg problems are important determinants of functional status in community dwelling older people. Disabil Rehabil 2005;27(16):917-23

8. Gillespie LD, Robertson MC, Gillespie WJ, et al. Interventions for preventing falls in older people living in the community. Cochrane Database Syst Rev 2009;2(CD007146)

9. Cameron ID, Murray GR, Gillespie LD, et al. Interventions for preventing falls in older people in nursing care facilities and hospitals. Cochrane Database Syst Rev 2010;1(1)

10. Spink MJ, Menz HB, Fotoohabadi MR, et al. Effectiveness of a multifaceted podiatry intervention to prevent falls in community dwelling older people with disabling foot pain: randomised controlled trial. BMJ 2011;342

11. Menz H, Morris M, Lord S. Foot and ankle risk factors for falls in older people: A prospective study. J Gerontol A Biol Sci Med Sci 2006;61(8):866 - 70

12. Mickle KJ, Munro BJ, Lord SR, et al. Foot pain, plantar pressures, and falls in older people: a prospective study. J Am Geriatr Soc 2010;58(10):1936-40

13. Mickle KJ, Munro BJ, Lord SR, et al. ISB Clinical Biomechanics Award 2009: toe weakness and deformity increase the risk of falls in older people. Clin Biomech (Bristol, Avon) 2009;24(10):787-91

14. Menant JC, Steele JR, Menz HB, et al. Optimizing footwear for older people at risk of falls. J Rehabil Res Dev 2008;45(8):1167 - 81

15. Hatton AL, Rome K, Dixon J, et al. Footwear interventions: a review of their sensorimotor and mechanical effects on balance performance and gait in older adults. J Am Podiatr Med Assoc 2013;103(6):516-33



For peer review only - http://bmjopen.bmj.com/site/about/guidelines.xhtml

For peer review only

16. Koepsell T, Wolf M, Buchner D, et al. Footwear style and risk of falls in older adults. J Am Geriatr Soc 2004;52(9):1495 - 501

17. Kelsey JL, Procter-Gray E, Nguyen U-SD, et al;. Footwear and falls in the home among older individuals in the MOBILIZE Boston study. Footwear science 2010;2(3):123 - 29

18. Menz HB, Morris ME, Lord SR. Footwear characteristics and risk of indoor and outdoor falls in older people. Gerontology 2006;52(3):174 - 80

19. Society AG. Guideline for the prevention of falls in older persons. J Am Geriatr Soc 2001;49(5):664 - 72

20. Australian Commission on Safety and Quality in Health Care. Preventing falls and harm from falls in older people: best practice guidelines for Australian community care. Australian Commission on Safety and Quality in Health Care, 2009. Available at http://www.safetyandquality.gov.au/publications/preventing-falls-and-harm-from-falls-in-older-people-best-practice-guidelines-for-australian-hospitals-2009/ (accessed 20/10/2014)

21. Balanowski K, Flynn L. Effect of painful keratoses debridement on foot pain, balance and function in older adults. Gait Posture 2005;22(4):302 - 07

22. Hijmans JM, Geertzen JHB, Dijkstra PU, et al. A systematic review of the effects of shoes and other ankle or foot appliances on balance in older people and people with peripheral nervous system disorders. Gait Posture 2007;25(2):316-23 doi: http://dx.doi.org/10.1016/j.gaitpost.2006.03.010 [published Online First: 9 May 2006]

23. Kobayashi R, Hosoda M, Minematsu A, et al. Effects of toe grasp training for the aged on spontaneous postural sway. J Phys Ther Sci 1999;11(1):31 - 34

24. Schwenk M, Jordan ED, Honarvararaghi B, et al. Effectiveness of foot and ankle exercise programs on reducing the risk of falling in older adults: A systematic review and meta-analysis of randomized controlled trials. J Am Podiatr Med Assoc 2013;103(6):534-47



For peer review only - http://bmjopen.bmj.com/site/about/guidelines.xhtml

25. Spink MJ, Menz HB, Lord SR. Efficacy of a multifaceted podiatry intervention to improve balance and prevent falls in older people: study protocol for a randomised trial. BMC Geriatr 2008;8(1):30

26. Sheikh JI, Yesavage JA. Geriatric Depression Scale (GDS): Recent evidence and development of a shorter version. In Clinical Gerontology: A Guide to Assessment and Intervention. Edited by Brink TL. NY, NY Haworth Press 1986:165-173.

27. Brink TL, Yesavage JA, Lum O, et al. Screening tests for geriatric depression. Clin Gerontol 1982;1(1):37 - 43

28. Pighills AC, Torgerson DJ, Sheldon TA, et al. Environmental assessment and modification to prevent falls in older people. J Am Geriatr Soc 2011;59(1):26 - 33

29. Vernon W. The Healthy Footwear Guide. Podiatry Now 2009;5:35

30. Kind P. The EuroQoL instrument: an index of health-related quality of life. Quality of life and pharmacoeconomics in clinical trials 1996;2:191 - 201

31. Kempen GI, Yardley L, Van Haastregt JC, et al. The Short FES-I: a shortened version of the falls efficacy scale-international to assess fear of falling. Age Ageing 2008;37(1):45 - 50

32. J Schuling, R de Haan, M Limburg et al. The Frenchay Activities Index. Assessment of functional status in stroke patients. Stroke 1993;24:1173 – 77.

33. Patton MQ. Qualitative Evaluation and Research Methods. 2nd edition: Newbury Park, CA: Sage 1990

34. Robertson MC, Campbell AJ, Herbison P. Statistical Analysis of Efficacy in Falls Prevention Trials. J Gerontol A Biol Sci Med Sci 2005;60(4):530-534

35. Silverman D. Interpreting Qualitative Data: Methods for Analysing Talk, Text and Interaction: Methods for Analyzing Talk, Text and Interaction. London, UK: Sage 1993.



For peer review only - http://bmjopen.bmj.com/site/about/guidelines.xhtml

36. Adamson J. Combined qualitative and quantitative designs. In: Bowling A, Ebrahim S eds. Handbook of health research methods: Investigation, measurement and analysis. Maidenhead UK: Open University Press 2005:230-245.

37. National Institute for Health and Care Excellence. Guide to the methods of technology apraisal: London: NICE, 2013. http://www.nice.org.uk/article/pmg9/chapter/Foreword http://www.nice.org.uk/article/PMG9/chapter/Foreword (accessed October 2014).

38. Brooks R. EuroQol: the current state of play. Health policy 1996;37(1):53 - 72

39. Matthews J, Altman DG, Campbell M, et al. Analysis of serial measurements in medical research. BMJ 1990;300(6719):230

40. Nixon RM, Thompson SG. Methods for incorporating covariate adjustment, subgroup analysis and between-centre differences into cost-effectiveness evaluations. Health Econ 2005;14(12):1217 - 29

41. O'Hagan A, Stevens JW, Montmartin J. Bayesian cost-effectiveness analysis from clinical trial data. Stat Med 2001;20(5):733-53

42. Willan AR, Lin D, Manca A. Regression methods for cost-effectiveness analysis with censored data. Stat Med 2005;24(1):131 - 45

43. Stinnett AA, Mullahy J. Net health benefits: a new framework for the analysis of uncertainty in cost-effectiveness analysis. Med Decis Making 1998;18(Suppl 2):S68-80

44. Claxton K. Exploring uncertainty in cost-effectiveness analysis. Pharmacoeconomics 2008;26(9):781 - 98

45. Van Hout BA, Al MJ, Gordon GS, et al. Costs, effects and C/E-ratios alongside a clinical trial. Health econ 1994;3(5):309 - 19

46. Manca A, Palmer S. Handling missing data in patient-level cost-effectiveness analysis alongside randomised clinical trials. Appl Health Econ Health Policy 2005;4(2):65 - 75

For peer review only



For peer review only - http://bmjopen.bmj.com/site/about/guidelines.xhtml

47. Relton C, Torgerson D, O'Cathain A, et al. Rethinking pragmatic randomised controlled trials: introducing the "cohort multiple randomised controlled trial" design. BMJ 2010;340

For peer review only



For peer review only - http://bmjopen.bmj.com/site/about/guidelines.xhtml

**REFORM study protocol Figure 1 Formthotic orthotic device**

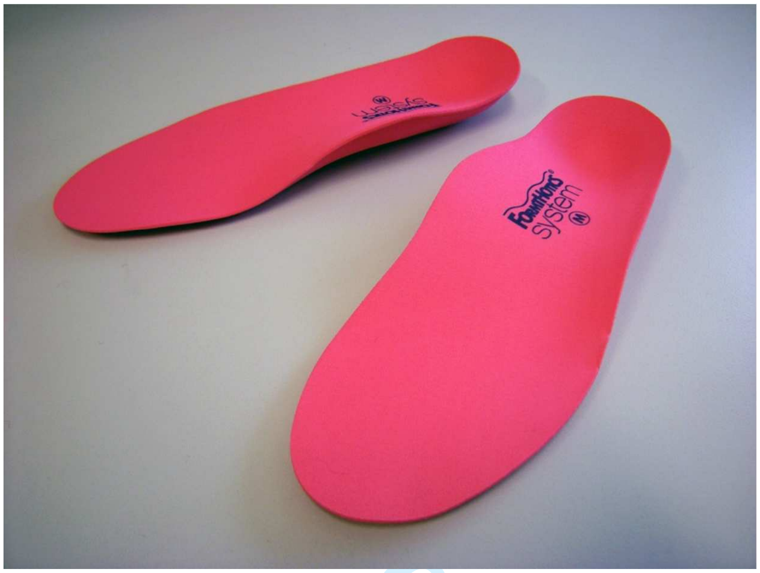


**Figure 2 X-line orthotic device**

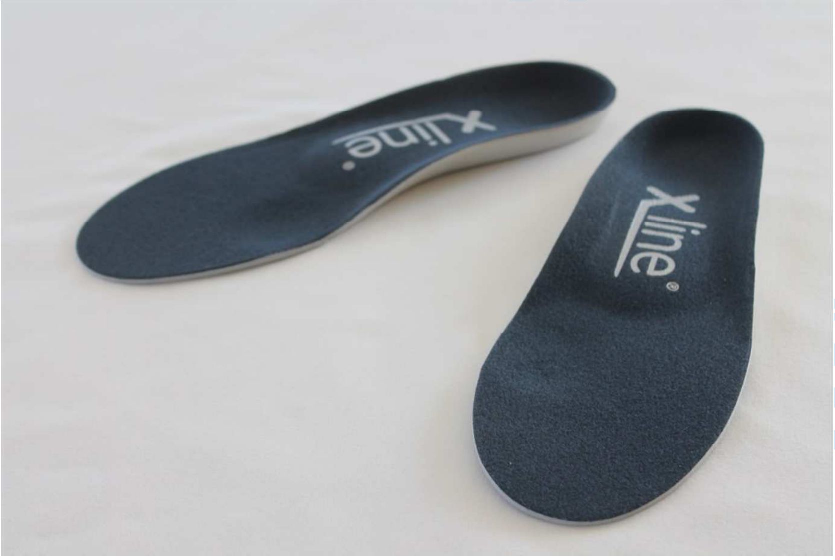



For peer review only - http://bmjopen.bmj.com/site/about/guidelines.xhtml

**REFORM study protocol Figure 1 Formthotic orthotic device**

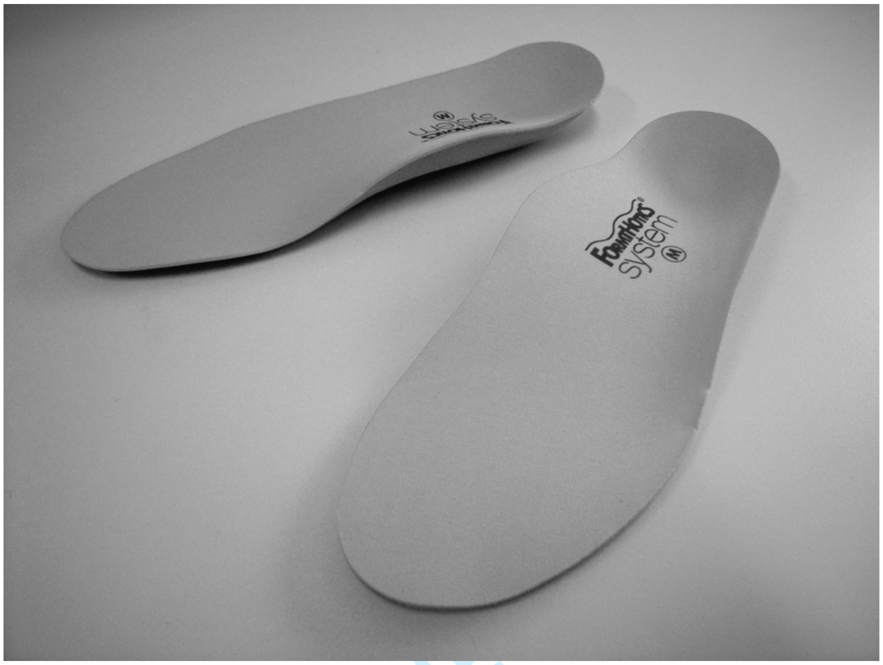

**Figure 2 X-line orthotic device**

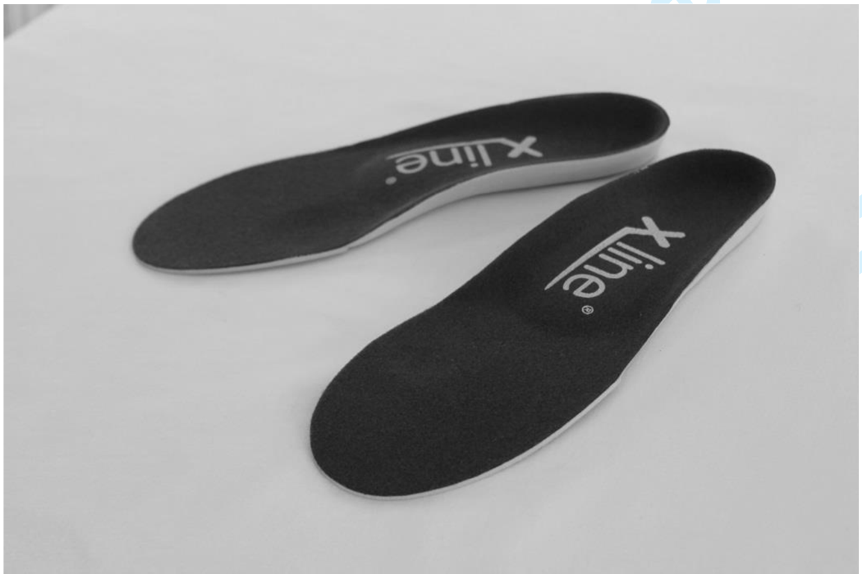

For peer review only - http://bmjopen.bmj.com/site/about/guidelines.xhtml

BMJ Open

# The REFORM study protocol: a cohort randomised controlled trial of a multifaceted podiatry intervention for the prevention of falls in older people

| | |
|---|---|
| Journal: | *BMJ Open* |
| Manuscript ID: | bmjopen-2014-006977.R1 |
| Article Type: | Protocol |
| Date Submitted by the Author: | 17-Nov-2014 |
| Complete List of Authors: | Cockayne, Sarah; University of York, Department of Health Sciences<br>Adamson, Joy; University of York, Department of Health Sciences<br>Corbacho, Belen; University of York, Department of Health Sciences<br>Fairhurst, Caroline; University of York, Department of Health Sciences<br>Hewitt, Catherine; University of York, Department of Health Sciences<br>Hicks, Kate; University of York, Department of Health Sciences<br>Hull, Robin; Harrogate and District NHS Foundation Trust, Podiatry Services<br>Keenan, Anne-Maree; University of Leeds, Leeds Institute of Rheumatology and Musculoskeletal Medicine; NIHR Leeds Musculoskeletal Biomedical Research Unit, Leeds Teaching Hospitals NHS Trust<br>Lamb, Sallie; University of Oxford, Nuffield Department of Orthopaedics, Rheumatology and Musculoskeletal Sciences, Kadoorie Critical Care Research Centre, John Radcliffe Hospital<br>Loughrey, Lorraine; Leeds Teaching Hospitals NHS Trust, NIHR Leeds Musculoskeletal Biomedical Research Unit; University of York, Department of Health Sciences<br>McIntosh, Caroline; National University of Ireland, School of Health Sciences, Discipline of Podiatry<br>Menz, Hylton; LaTrobe University, Faculty of Health Sciences<br>Redmond, Anthony; University of Leeds, Leeds Institute of Rheumatology and Musculoskeletal Medicine; Leeds Teaching Hospitals NHS Trust, NIHR Leeds Musculoskeletal Biomedical Research Unit<br>Rodgers, Sara; University of York, Department of Health Sciences<br>Vernon, Wesley; Sheffield Teaching Hospitals NHS Foundation Trust, Podiatry<br>Watson, Jude; University of York, Department of Health Sciences<br>Torgerson, David; The University of York, |
| <b>Primary Subject Heading</b>: | Health services research |
| Secondary Subject Heading: | Health economics, Research methods, Qualitative research, Geriatric medicine |
| Keywords: | HEALTH ECONOMICS, STATISTICS & RESEARCH METHODS, Musculoskeletal disorders < ORTHOPAEDIC & TRAUMA SURGERY, QUALITATIVE RESEARCH |
| | |

For peer review only

# The REFORM study protocol: a cohort randomised controlled trial of a multifaceted podiatry intervention for the prevention of falls in older people

Sarah Cockayne[1§], Joy Adamson[1], Belen Corbacho Martin[1], Caroline Fairhurst[1], Catherine Hewitt[1], Kate Hicks[1], Robin Hull[2], Anne-Maree Kenan[3,4], Sarah E Lamb[5], Lorraine Loughrey[1,3], Caroline McIntosh[6], Hylton B Menz[7], Anthony C Redmond[3, 4], Sara Rodgers[1], Wesley Vernon[8], Judith Watson[1], David Torgerson[1] on behalf of the REFORM study

[1]York Trials Unit, Department of Health Sciences, University of York, YO10 5DD, UK

[2] Podiatry Services, Harrogate and District NHS Foundation Trust, Harrogate District Hospital, Lancaster Park Road, UK

[3]NIHR Leeds Musculoskeletal Biomedical Research Unit, Chapel Allerton Hospital, Leeds, UK

[4]Leeds Institute of Rheumatology and Musculoskeletal Medicine, University of Leeds

[5]Nuffield Department of Orthopaedics, Rheumatology and Musculoskeletal Sciences, Kadoorie Critical Care Research Centre, John Radcliffe Hospital, University of Oxford, UK

[6]National University of Ireland, Galway, Republic of Ireland

[7]Lower Extremity and Gait Studies Program, Faculty of Health Sciences, La Trobe University, Bundoora 3086, Victoria, Australia

[8]Podiatry Services, Sheffield Teaching Hospitals NHS Foundation Trust, Jordanthorpe Health Centre, 1 Dyche Close, Sheffield, UK

[§]Corresponding author

Email addresses:



For peer review only - http://bmjopen.bmj.com/site/about/guidelines.xhtml

§SC: sarah.cockayne@york.ac.uk telephone number 01904 321736

JA: joy.adamson@york.ac.uk

BC: belen.corbacho@york.ac.uk

CF: caroline.fairhurst@york.ac.uk

CH: catherine.hewitt@york.ac.uk

KH: kate.hicks@york.ac.uk

RH: Robin.Hull@hdft.nhs.uk

AMK: a.keenan@leeds.ac.uk

SEL: sarah.lamb@ndorms.ox.ac.uk

LL: L.R.Loughrey@leeds.ac.uk

CM: caroline.mcintosh@nuigalway.ie

HBM: h.menz@latrobe.edu.au

ACR: a.redmond@leeds.ac.uk

SR: sara.rodgers@york.ac.uk

WV: wesley.vernon@nhs.net

JW: jude.watson@york.ac.uk

DT: david.torgerson@york.ac.uk

**Key words**

Cohort randomised controlled trial, multifaceted podiatry intervention, prevention of falls.

Word count 6919



For peer review only - http://bmjopen.bmj.com/site/about/guidelines.xhtml

For peer review only

## ABSTRACT


### Introduction

Falls and fall related injuries are a serious cause of morbidity and cost to society. Foot problems and inappropriate footwear may increase the risk of falls; therefore podiatric interventions may play a role in reducing falls. Two Cochrane systematic reviews identified only one study of a podiatry intervention aimed to reduce falls, which was undertaken in Australia. The REFORM trial aims to evaluate the clinical and cost-effectiveness of a multifaceted podiatry intervention in reducing falls in people aged 65 years and over in a UK and Irish setting.

### Methods and analysis

This multicentre, cohort randomised controlled trial will recruit 2,600 participants from routine podiatry clinics in the UK and Ireland to the REFORM cohort. In order to detect a 10% point reduction in falls from 50% to 40%, with 80% power 890 participants will be randomised to receive routine podiatry care and a falls prevention leaflet or routine podiatry care, a falls prevention leaflet and a multifaceted podiatry intervention.  The primary outcome is rate of falls (falls/person/time) over 12 months assessed by patient self-report falls diary. Secondary self-report outcome measures include: the proportion of single and multiple fallers and time to first fall over a 12 month period; Short Falls Efficacy Scale – International; fear of falling in the past four weeks; Frenchay Activities Index; fracture rate; Geriatric Depression Scale; EQ-5D 3-L; health service utilisation at 6 and 12 months. A qualitative study will examine the acceptability of the package of care to participants and podiatrists.

### Ethics and dissemination




For peer review only - http://bmjopen.bmj.com/site/about/guidelines.xhtml

The trial has received a favourable opinion from the East of England - Cambridge East Research Ethics Committee and Galway Research Ethics Committee. The trial results will be published in peer-reviewed journals and at conference presentations.

**Trial registration number** Current Controlled Trials ISRCTN68240461assigned 01/07/2011

**Strengths and limitations of this study**

- This study is the first UK study evaluating the clinical and cost-effectiveness of podiatric care combined with footwear advice and provision (if required), orthotic inserts and foot and ankle exercises for falls prevention in people over 65 years of age.
- This study uses the novel cohort randomised controlled trial design.
- The study uses an unblinded, patient self-report primary outcome measure.
- It will not be possible to determine the clinical and cost-effectiveness of this intervention in patients who fall but do not receive routine podiatry care.



For peer review only - http://bmjopen.bmj.com/site/about/guidelines.xhtml

## BACKGROUND

Falls and fall related injuries are a serious cause of morbidity and cost to society [1], a burden which will increase with an ageing population. The National Service Framework (NSF) for Older People [2] recognises the importance of fall related injuries, and calls for health improvement plans to be devised that will reduce this burden.

It is well recognised that falls result from interactions between environmental hazards, a variety of medical conditions and physiological risk factors [3]. Foot problems may also increase the risk of falls. Foot problems affect one in three community dwelling people over the age of 65 years [4] and are associated with reduced walking speed and difficulty in performing activities of daily living [5-7].

There have been two relevant Cochrane reviews on falls prevention, one relating to falls in community dwelling older people [8] and one focusing on falls in hospitals and aged care facilities [9]. At the time of designing the study neither identified any randomised controlled trials (RCTs) focusing on podiatry-related interventions. A subsequent update has identified one trial of a podiatry based intervention for the prevention of falls [10] which related to the Australian health care system

There is some evidence to suggest that foot problems are associated with an increased risk of falling. Menz *et al's* [6] prospective study of 176 older people identified ankle flexibility, toe plantarflexor strength and plantar sensation as significant and independent predictors of balance and functional test performance. These factors were later confirmed as predictors of falling during a 12 month follow up of that cohort, in which foot pain was also identified as a



For peer review only - http://bmjopen.bmj.com/site/about/guidelines.xhtml

predictor of falling [11]. Mickle *et al's* [12, 13] prospective study of 312 people over 60 years of age also found that fallers had significantly higher prevalence of foot pain, and displayed significantly less strength of the hallux and were more likely to have hallux valgus and lesser toe deformities.

Inappropriate footwear may also impair balance and increase the risk of falling. Footwear characteristics considered detrimental to balance include high heels, soft soles and inadequate slip resistance [14, 15]. Prospective studies have shown that walking barefoot or wearing only stockings inside the home and wearing shoes with an increased heel height and smaller contract area increases the risk of falling [16-18]. People who have a history of falling are at increased risk of falls. [19]

Given the emerging evidence that foot problems and inappropriate footwear increase the risk of falling, it has been suggested that podiatry may have a role to play in falls prevention, with several guidelines recommending that older people have their feet and footwear examined by a podiatrist [20, 21].

Several studies have also suggested that some treatments provided by podiatrists, such as lesion debridement [22], foot orthoses [23], foot and ankle exercises [24, 25] and footwear advice may play a role in improving balance. Combining these therapies should allow for stability and improved function to be achieved at each level. Lesion debridement can improve function during gait if pain is reduced, exercise programmes focus on internal strengthening and flexibility, and appropriate footwear with orthotic devices can provide external support, improved kinaesthesia and improved function. A trial conducted in Australia in 305 community dwelling older people with disabling foot pain showed a 36%



For peer review only - http://bmjopen.bmj.com/site/about/guidelines.xhtml

For peer review only

reduction in falls for those receiving a multifaceted podiatry intervention, consisting of a foot orthosis, advice on footwear, subsidy for new footwear, a home based programme of foot and ankle exercises, a falls prevention education booklet and routine podiatry care for 12 months [10, 26]. The control group received only routine podiatry care for 12 months which consisted of toenail maintenance and scalpel debridement of hyperkeratotic lesions (corns and calluses). There have been no further large pragmatic trials looking at podiatric care combined with footwear advice, foot and ankle exercise and orthotic inserts for falls prevention, and there has been no assessment of whether such an intervention is economically viable within the UK health care setting. The REFORM (REducing Falls with ORthoses and a Multifaceted podiatry intervention) trial aims to address these issues. The main objectives are to investigate the clinical and cost-effectiveness of a multifaceted podiatry intervention for falls prevention and to assess views and experiences of the intervention and the trial process from the perspective of the participants and podiatrists.

## METHODS AND ANALYSIS

### Design

The REFORM study is a "cohort randomised controlled trial" (cRCT). Firstly we will recruit participants to the REFORM cohort and whilst this is being assembled, we will invite a selection of eligible participants from the site undertaking the pilot phase to take part in a REFORM pilot study. Once the pilot study is complete, we will then invite the remaining eligible participants to take part in the main REFORM trial. The REFORM main trial is a two arm, pragmatic, open, multicentre, randomised controlled trial with a 12 month follow-up.



For peer review only - http://bmjopen.bmj.com/site/about/guidelines.xhtml

**REFORM study objectives for the pilot phase**

(i) To demonstrate the feasibility of recruiting to the REFORM cohort

(ii) To develop and pilot the multifaceted podiatry intervention including a foot and ankle exercise programme, supplementary DVD and booklet with approximately 60 participants

(iii) To develop the podiatrist training package

(iv) To pilot the falls calendar and other patient data collection questionnaires

(v) To assess participants' views and experiences of the intervention and the trial process

(vi) Pilot, review and refine if necessary recruitment methodology for the main trial

All these objectives were achieved and the pilot has moved seamlessly into the main REFORM trial.

**REFORM study objectives for the main study**

This study will aim to address the following:

(i) To train the podiatrists to deliver the intervention

(ii) To examine the clinical and cost-effectiveness of the multifaceted podiatry intervention for falls prevention

(iii) To assess the podiatrists' views and experiences of the intervention and trial process

**Participants**

Participant recruitment

Participants will be recruited from NHS podiatry clinics based in either primary or secondary care in the UK and one international site in the Republic of Ireland. The rationale for



For peer review only - http://bmjopen.bmj.com/site/about/guidelines.xhtml

recruiting only from podiatry clinics is due to the logistical constraints imposed by the requirement for the control group to receive routine podiatry care. Whilst it would be possible to recruit from GP practices, many of the patients identified in this setting would not be receiving routine podiatry care, and NHS podiatry service managers indicted that they would not have the capacity to see large numbers of additional patients.

Podiatrists within the NHS Trust or clinic at the Republic of Ireland or the REFORM research podiatrist will undertake a search of either electronic or paper patient medical notes to identify potential participants for the study. Patients aged 65 years and over who are registered with the service and have attended routine podiatry services within the past six months of the search being undertaken will be identified and will be eligible for an invitation mailing. Patients who have attended high risk clinics, (for example diabetes clinics), or who live in a nursing home will be excluded from the invitation mail out. Sites will be requested where possible to screen out patients in the following groups: patients with a life expectancy of less than six months; patients known to have dementia, a neurodegenerative disorder, neuropathy, a lower limb amputation, or are chair or bed bound. All eligible patients will be sent an invitation pack (letter of invitation, participant information sheet, consent form, screening questionnaire and pre-paid envelope) asking whether they would like to participate in the REFORM study. Where the clinic has the capacity, potential participants attending routine podiatry clinics may be approached opportunistically. Where a multidisciplinary team exists, other healthcare professionals such as occupational therapists, falls practitioners and physiotherapists may support the opportunistic recruitment.

Participants wishing to take part in the REFORM study will be asked to return their completed consent form and screening questionnaire by post to the York Trials Unit (YTU).



For peer review only - http://bmjopen.bmj.com/site/about/guidelines.xhtml

Researchers at the YTU will assess the returned screening forms for participant eligibility. During the consenting stage, potential participants will be informed of the possibility of participating in other related studies, e.g. an associated qualitative study and they will be asked to indicate (by ticking a box on the consent form), if they would prefer not to be approached about these studies. Participants can withdraw from the study at any point. The reason for withdrawal will not have to be declared; however, if provided, this will be recorded. Data will be retained for all participants up to the date of withdrawal, unless a participant specifically requests their details be removed. All data returned to the York Trials Unit by participants will be held in accordance with the Data Protection Act 1998.

**Inclusion criteria for the REFORM cohort**

All eligible, consenting participants will be asked to complete a baseline questionnaire. Participants who return valid baseline data will be included in the REFORM cohort. Any participant reporting a score of 10 or more on the Geriatric Depression Scale [27, 28] i.e. more severe depression, will be referred to their general practitioner (GP).

**Exclusion criteria for the REFORM cohort**

Participants will be ineligible for the REFORM cohort if they are under 65 years of age; report having neuropathy, dementia or other neurological condition such as Parkinson's disease, Alzheimer's, multiple sclerosis, Lou Gehrig's/amyotrophic lateral sclerosis or Huntington's disease; are unable to walk household distances (10 metres) without the help of a walk aid such as a Zimmer frame or walker or person to assist; have had a lower limb amputation; or are unwilling to attend their podiatry clinic.

**Inclusion criteria for the REFORM trial**



For peer review only - http://bmjopen.bmj.com/site/about/guidelines.xhtml

For peer review only

Participants will be eligible for the REFORM trial if they:

(i) have had one fall in the past 12 months; or one fall in the past 24 months requiring hospital attention; or report a fear of falling on their baseline questionnaire i.e. have worried about falling at least some of the time, in the past four weeks

(ii) are community dwelling

**Exclusion criteria for the REFORM Trial**

Participants who do not complete the baseline or run-in data collection instruments adequately, or who are unable to read or speak English will be excluded from the trial**.**

**Participants who do not wish to take part in the study**

Participants who do not wish to take part in the main study are not required to return any forms to the YTU. However, all participants in the pilot phase of the study who are sent an invitation pack will be given the opportunity to decline participation and if willing, provide some demographic information and reason for declining. This will provide us with sufficient information to document the reasons why participants do not wish to take part in the study and will allow us to compare decliners to those who are participating. The recruitment pack in the main mail out will not contain a decline form.

**Sample size**

The REFORM cohort

We propose to recruit up to 2,600 participants into the REFORM cohort to allow us to sample for the pilot trial and to allow cohort attrition before we sample for the main REFORM trial.



For peer review only - http://bmjopen.bmj.com/site/about/guidelines.xhtml

Internal pilot phase

Pilot trial: In order to pilot the intervention, a random sample of 60 participants will be selected from the REFORM cohort. Participants will be allocated equally to each of the two groups i.e. 30 participants per group. The allocation sequence will be computer generated by the YTU randomisation service and will be stratified by centre.

REFORM main trial

The REFORM trial is designed to detect a 10% point reduction in the number of people who fall over a 12 month period. Assuming this high risk group has an underlying risk of falling in a 12 month period of 50% [29] then in order to observe a reduction to 40% with 80% power and a two-sided 5% significance level we would require 890 participants (445 in each group, allowing for a 10% loss to follow up).

**Randomisation**

Participants who fulfil the eligibility criteria for the REFORM trial and who have provided written consent to take part in the study will be eligible for randomisation. Randomisation will be carried out by the YTU secure remote computer randomisation service. Eight hundred and ninety participants will be randomly allocated in a 1:1 ratio to either the intervention or control group. If more than 890 eligible participants are recruited then we will randomly allocate the remaining participants in a 2:1 ratio in favour of the control group. This will allow us to increase the power of the study, without putting patients at any additional risk and without incurring additional costs. In order to allow for manageable case-loads at individual sites, the randomisation is by permuted blocks and stratified by centre. The YTU will write to the participant's GP informing them of study participation and to participants who are allocated to the intervention group.



For peer review only - http://bmjopen.bmj.com/site/about/guidelines.xhtml

For peer review only

**Blinding**

Due to the nature of the intervention, blinding of participants will not be feasible. Blinding of members of the study team who are actively involved in the administration of the study and may collect primary outcome falls data or undertake data queries on secondary outcomes, or the health economist will not be possible. Members of the study team responsible for data entry and the statistical analysis of the study will be kept blind to group allocation.

**Intervention group**

Participants allocated to receive the intervention will be seen by the podiatrist at the clinic they would normally attend for routine podiatry care, as soon as possible after randomisation. Participants are invited to attend two podiatry visits approximately two to four weeks apart, with further appointments if required in addition to their usual podiatry care.

(i) Footwear advice and provision

Participants' everyday footwear (indoor and outdoor footwear) will be assessed according to the following characteristics: appropriate size; method of fastening; height and width of the shoe's heel; thickness of outsole; heel counter stiffness; longitudinal sole rigidity; sole flexion point and tread pattern. Footwear will be deemed to be inappropriate if the shoes have any of the following characteristics: (i) heel height is greater than 4.5cm; (ii) no adjustable fixation of the upper; (iii) no heel counter or the heel counter can be depressed to greater than 45°; (iv) a fully worn/smooth/thin sole; (v) the shoe heel width is narrower than the participant's heel width by greater than or equal to 20%; or (vi) incorrect shoe size [10]. Participants with inappropriate footwear will be counselled regarding the hazardous footwear feature(s) identified during the assessment and will be advised on safer characteristics for future



For peer review only - http://bmjopen.bmj.com/site/about/guidelines.xhtml

For peer review only

footwear purchases. Where possible, footwear will be provided for participants whose footwear is deemed to be inappropriate. The podiatrist will order shoes directly from two companies participating in the Healthy Footwear Guide (HFG) scheme [30]; DB shoes (Rushden, UK) or Hotter company (Skelmersdale, UK). As not all of the shoes manufactured by these companies fulfil the characteristics of a safe shoe, the trial team will assess the shoes and provide a list of suitable footwear from which participants can choose. In order to avoid incentivising patients to take part in the study, participants will only be told about footwear provision, if they are deemed to need new footwear. During the REFORM pilot trial we will explore the feasibility of making a footwear assessment, providing footwear advice and provision of footwear and if necessary we will revise the intervention if needed.

(ii) Foot orthoses

We originally planned to use the same orthotic device (Formthotics™, Foot Science International, Christchurch, NZ) (Figure 1) as our Australian collaborator [10, 26] as they had demonstrated that this device, commonly used in Australia, was acceptable to patients and was shown to be associated with a reduction in falls. However, during the setup period of the pilot phase of the REFORM trial, podiatrists at the recruiting sites reported difficulties when fitting these devices with the addition of posting (the application of wedges under the forefoot or rearfoot) and the ease of fitting in patient's current shoes. These sites frequently use the x-line® orthoses range (Healthystep, Mossley, UK) (Figure 2) as they are easy to modify and are easy to fit in patient's current shoes. During the pilot trial we will provide participants with both types of device to determine which device is most acceptable to a UK population. The Formthotics™ device will be issued as per the manufacturing guideline using the appropriate heating equipment and fitting procedure. The x-line® PressurePerfect/Standard device will be fitted and if indicated, modified to improve foot posture as assessed in usual



For peer review only - http://bmjopen.bmj.com/site/about/guidelines.xhtml

clinical practice by the individual clinician. If a participant already has insoles, whether shop bought or prescribed, the clinician will make a clinical judgement on the suitability of replacing their current insole with the trial insole. If the current insole is replaced with the trial insole then any current prescription/modifications maybe repeated, if applicable. We would consider the insole component of the intervention already addressed if the clinician felt it detrimental to replace their current insole with that of the trial insole. If the podiatrist feels that the patient requires more than what the current or trial insole can provide, or if they present with a musculoskeletal pathology, then a referral will be made in line with routine practice.

(iii) Home-based foot and ankle exercise programme

Participants will be prescribed a 30 minute home-based foot and ankle exercise programme which will be undertaken three times per week, indefinitely. The exercise programme is aimed at stretching and strengthening the muscles of the foot and ankle. The exercises will be based on the programme developed by Spink *et al* [10, 26] and will be adapted to make it suitable for a UK setting during the pilot phase of the trial. A summary of the individual exercises is given in Table 1. The exercises will be demonstrated by the podiatrist and will be supplemented by a DVD and an illustrated explanatory booklet. Participants will be assessed for competence and safety at the baseline and follow-up appointments.

(iv) Routine podiatry care

Routine podiatry care will continue to be given at separate podiatry appointments in accordance with participant's usual care. This will aim to reduce painful conditions such as corns and callouses, that have been found to be associated with an increased risk of falls.



For peer review only - http://bmjopen.bmj.com/site/about/guidelines.xhtml

**Table 1 Summary of the home based foot and ankle exercises**

| Activity | Description | Dosage | Increments |
|---|---|---|---|
| Ankle range of motion/ warm up | Sitting with the knee at 90 degrees. Lift the foot to clear the ground and then rotate the foot slowly in a clockwise direction and then an anti-clockwise. | 1x10 repetitions for each foot in each direction. | None. |
| Ankle inversion strength | Sitting upright, hip, knee and ankle at 90º. Invert foot against resistive exercise band. The band should be fixed at 90 degrees to the foot from an additional chair/table leg. | 3x10 repetitions for each foot. | Increase resistance strength of resistive exercise band. |
| Ankle eversion strength | Sitting upright, hip, knee and ankle at 90º. Evert foot against resistive exercise band The band should be fixed at 90 degrees to the foot from an additional chair/table leg | 3x10 repetitions for each foot. | Increase resistance strength of resistive exercise band. |
| Ankle dorsiflexion strength | Sitting, hip, knee and ankle at 90º.<br>Dorsiflex both feet to end range of motion and hold. Keep pulling feet up towards the body during the hold. | Hold feet in dorsiflexion for 3x10 seconds. | Increase repetitions up to maximum of 10. |
| Intrinsic strengthening, toe plantarflexion strength and toe stretch | Sitting, hip, knee and ankle at 90 º.<br>1) Use the therapy ball under the toes to stretch the toes. The rest of the foot should be plantargrade. Then curl and point the toes up and over the ball.<br>2) Use the therapy ball under the toes to stretch the toes. The rest of the foot should be plantargrade. With the heel on/close to the floor, curl the toes over the ball and attempt to pick up the ball with the toes. | 3 x10 repetitions for each exercise both feet. Have a 30 sec break between each repetition | Increase up to a maximum of 50 repetitions. |
| Ankle plantarflexion strength | From standing position, rise up onto toes of both feet and then slowly lower back down. | 3x10 repetitions. | Increase repetitions up to maximum of 50. |



For peer review only - http://bmjopen.bmj.com/site/about/guidelines.xhtml

| | | | |
|---|---|---|---|
| | Just before the heels contact the floor, rise back up onto the toes. | | |
| Calf stretch | Facing a wall and using hands on the wall for balance, step one foot in front of the other keeping feet hip width apart and hips, knees and feet facing the wall. Bend the knee closest to the wall and keep the back leg straight. Keep both heels in contact with the floor. | Hold stretch for 3x20 seconds on each leg. | Increase the stride length and forward lean to increase the stretch |
| Proprioception/balance training | From a standing position and holding on to a work surface/chair/wall for support, stand on one leg. Repeat on the other side | Hold for 30 seconds. Repeat 3 repetitions. | Increase slowly to hold for 1 min per repetition. If competent, rise up on to toes on the one supporting leg: 3 x 10 repetitions. |

(vi) Falls prevention leaflet and trial newsletter

Participants will receive a copy of the latest falls prevention advice leaflet produced by Age UK (the current version 'Staying steady' was printed in June 2010). This leaflet will be sent to the participant in the post with their baseline questionnaire.

Participants will be sent a site and group specific newsletter at three and 12 months informing them about progress of the study. The intervention group's newsletter will include a section about the foot and ankle exercises in order to aid compliance. All participants will receive £5 in recognition of their participation and to offset any incidental expenses associated with completing the questionnaires.



For peer review only - http://bmjopen.bmj.com/site/about/guidelines.xhtml

**Control group**

Participants allocated to the control group will receive the same falls prevention advice leaflet produced by Age UK. This leaflet will be sent to the participant in the post with their baseline questionnaire. Participants will continue to receive routine podiatry and GP access in accordance with their usual care. Participants will also be sent a site and group-specific newsletter at three and 12 months. All participants will receive £5 in recognition of their participation and to offset any incidental expenses associated with completing the questionnaires.

**Primary outcome measure for the REFORM trial**

The primary outcome is the rate of falls (i.e. falls/person/time) where a fall is defined as "an unexpected event in which the participant comes to rest on the ground, floor, or lower level" [31]. Data will be collected via participant self-reported monthly falls calendars throughout the 12 months following randomisation. Participants will be asked to record each day whether or not they had any fall. Participants who do not return their falls calendar within one week of the due date will be telephoned by the YTU to obtain the missing data. Participants will also be given a Freephone number to report any falls as soon as possible after the fall. Information collected includes: date and location of fall; reason for fall; any injuries sustained (e.g. a superficial wound or a broken bone); hospital admissions; footwear worn at the time of the fall; and if the patient was wearing an orthotic or using a walking aid. As we are collecting falls data at six and 12 months follow up, these data will be used for those participants who do not return their monthly falls calendar.



For peer review only - http://bmjopen.bmj.com/site/about/guidelines.xhtml

**REFORM trial secondary outcome measures**

All secondary outcomes are self-reported by the patient and collected by questionnaire at baseline, six and 12 months, or by monthly falls calendars. Secondary outcomes include: proportion of fallers (single and multiple); patient reported time to first fall during follow-up; health related quality of life as measured by the EQ-5D [32]; fear of falling as measured by the question, "During the past 4 weeks have you worried about having a fall?", fear of falling as measured by the Short Falls Efficacy Scale – International (FES-I) [33], activities of daily living as measured by the Frenchay Activities Index (FAI) [34], fracture rate, health service utilisation and depression as measured by the short form Geriatric Depression Scale (GDS) [28].

**Nested qualitative study**

A qualitative evaluation will be carried out to examine the acceptability of the intervention as a whole package of care, to both the trial participants and the podiatry practitioners. Trial participants from the pilot phase of the study who are receiving the intervention will be asked about their experience of the intervention. Particular attention will be paid to the acceptability and compliance with the foot orthosis, exercise programme, podiatry service and footwear advice/purchase and how the intervention fits into the individual's wider experience of balance problems within their everyday lives.

Ten to 15 participants will be purposively selected from the pilot phase of the trial (the first 60 participants randomised) to ensure a representative spread according to falls history, age and gender. This maximum variation sampling approach [35] will ensure a wide range of viewpoints are included in the data collection and analysis. Participants will be invited to attend a face-to-face interview or, if prefered, a telephone interview. Written informed



For peer review only - http://bmjopen.bmj.com/site/about/guidelines.xhtml

consent will be obtained from the participant prior to the interview. The semi-structured interviews will be conducted within approximately two weeks of receiving the six month follow-up questionnnaire. A follow-up telephone interview will be conducted within two weeks of receiving the 12 month follow-up questionnaire to discuss any changes (or not) recorded in the outcomes measured over the longer term follow-up period. All interviews will be conducted using a topic guide to ensure consistency across participants. However, the format will be flexible in order to allow participant-led data regarding what they constitute as important and/or successful in terms of treatment outcome.

We will interview a purposive sample of five to ten practitioners who are providing the podiatry services for the study regarding the way in which they have delivered the intervention, the ease of delivery, their confidence in the intervention, and their experience of being involved in the trial. Face-to-face or telephone interviews will be conducted approximately three months into the trial process. Informed consent will be obtained prior to the interview being conducted.

**Adverse events**

Details of any adverse events reported to the YTU either directly by the participant or by a member of the research team at the recruiting site will be recorded.

This study will report details of any serious adverse events (SAEs) that are required to be reported to the Research Ethics Committee (REC) under the current terms of the Standard Operating Procedures for RECs.

A SAE is defined as any untoward occurrence that:



For peer review only - http://bmjopen.bmj.com/site/about/guidelines.xhtml

For peer review only

(i) Results in death;

(ii) Is life threatening;

(iii) Requires hospitalisation or prolongation of existing hospitalisation;

(iv) Results in persistent or significant disability or incapacity;

(v) Consists of a congenital anomaly or birth defect, or

(vi) Is otherwise considered medically significant by the investigator.

An event is defined as 'related' if the event was due to the administration of any research procedure. An 'unexpected event' is defined as a type of event not listed in the protocol as an expected occurrence. Expected events include: aches and pains in the lower limb lasting for longer than 48 hours; fall; new callus/corn formation, blisters, ulcers; skin irritation/injury including pressure sores and soft tissue injury.

In the context of this study, an occurrence of the type listed in (i) to (vi) will be reported as an SAE only if:

(i)

the event is suspected to be related to an aspect of the research procedures (e.g. wearing the orthotic, undertaking the exercise programme, completion of follow-up questionnaires, participation in feasibility or qualitative sub-studies, telephone contact), and;

(ii)

it is an unexpected occurrence. Hospitalisations, disabling / incapacitating / life-threatening conditions, falls and deaths are expected in the study population due to the age of the cohort, they will therefore only be reported as SAEs if they appear to be related to an aspect of taking part in the study.



For peer review only - http://bmjopen.bmj.com/site/about/guidelines.xhtml

**Other data collected**

Treatment details will be recorded by the podiatrist including: the number of podiatry visits; an eligibility checklist with details on relevant health conditions and test results; characteristics of current indoor and outdoor shoes; details relating to shoes ordered; details on the type and prescription of any current insole use; the type of insole issued/retained with any modifications made, and any amendments or advice given on the intervention due to safety reasons.

Information on adherence to the exercise, footwear advice and orthotic components of the intervention will be collected from participant self-reported questionnaire data at three, six and 12 months. Participants will be asked whether during the past month, they were wearing their orthotic 'all of the time', 'most of the time', 'some of the time', 'a little of the time' or 'none of the time'. Participants will also be asked, for the past month, typically how many times a week they did the exercises: none, one, two, three, or more than three times. Participants will be asked at 12 months if they were given footwear advice, and whether or not they followed any given advice.

**Statistical analysis**

There will be a single analysis at the end of the trial, conducted using STATA (StataCorp, 4905 Lakeway Drive, College Station, Texas 77845 USA). All analyses will be conducted on an intention to treat basis, i.e. including all randomised patients in the groups to which they were assigned. All tests will be two-sided at the 5% significance level. If more than 890 participants are recruited and unequal allocation is utilised, then analyses will be adjusted to take this into account.



For peer review only - http://bmjopen.bmj.com/site/about/guidelines.xhtml

For peer review only

(i) The REFORM cohort

Descriptive statistics will be presented for participants in the REFORM cohort.

(ii) REFORM trial

Participant baseline data will be summarised descriptively by randomised arm. No formal statistical comparisons will be undertaken. Continuous measures will be reported as means and standard deviations whilst the categorical data will be reported as counts and percentages.

(iii) Statistical analysis of the REFORM trial primary outcome

The number of falls per person will be analysed using a Poisson regression model adjusting for gender, age, centre and history of falling to estimate the difference in falls rate between the groups. If there is over dispersion, a negative binomial regression model adjusting for the same factors will be used [36]. Point estimates and their associated 95% confidence intervals will be provided.

(iv) Statistical analysis of the REFORM trial secondary outcomes

The proportion of fallers versus non-fallers in each group will be compared by logistic regression adjusting for gender, age, centre and history of falling. Odds ratios and their associated 95% confidence intervals will be provided. The proportion of multiple fallers versus single or non-fallers in each group will be compared over the 12 month trial period using logistic regression adjusting for gender, age, centre and history of falling.

The time to the first fall will be derived as the number of days from randomisation until the patient reports having a fall as detailed from the participant’s falls calendar, falls telephone



For peer review only - http://bmjopen.bmj.com/site/about/guidelines.xhtml

data collection sheet or self-reported questionnaire. Participants who have not had a fall will be treated as censored at their date of trial exit, or date of last available assessment or 365 days/trial cessation, as appropriate. The proportion of patients yet to experience a fall will be summarised by a Kaplan Meier survival curve for each group. The time to first fall will be analysed by Cox Proportional Hazard regression adjusting for gender, age, centre and history of falling. Hazard ratios and their associated 95% confidence intervals will be provided. The proportional hazard assumption will be evaluated using Schoenfeld residuals. The median time to the first fall and its associated 95% confidence intervals will be estimated from this adjusted model.

Patients with a score of six or more on the short form Geriatric Depression Scale will be categorised as having depression. The proportion of people with depression in each group will be compared over the 12 month trial period using logistic regression adjusting for gender, age, centre and history of falling.

The following secondary outcomes: Short Falls Efficacy Scale - International, fear of falling in the past four weeks, and the Frenchay Activities Index will be treated as continuous data and will be measured at baseline, month six and month 12. A covariance pattern model incorporating all post randomisation time points will be used to compare the two groups on these outcomes adjusting for baseline score, gender, age, centre and history of falling. The correlation of observations within patients over time will be modelled and the model effects and their associated 95% confidence intervals will be provided.

The proportion of patients obtaining at least one fracture over the 12 month follow-up period will be compared by logistic regression adjusting for gender, age, centre and history of



For peer review only - http://bmjopen.bmj.com/site/about/guidelines.xhtml

falling. Odds ratios and their associated 95% confidence intervals will be provided. If there are patients that obtain multiple fractures in this time (from different events), then the proportion of patients obtaining multiple fractures versus single or no fractures in each group will be compared over the 12 month trial period using logistic regression adjusting for gender, age, centre and history of falling.

(v) Intervention adherence

Descriptive statistics will be used to summarise the adherence to the exercise and orthotic at three, six, and 12 months and adherence to following footwear advice. A Complier Average Causal Effect (CACE) analysis to assess the impact of compliance on treatment estimates will be considered.

(vi) Missing data

The amount of missing data will be reported for each randomised arm.

(vii) Qualitative analysis

All interviews will be audio recorded digitally and transcribed verbatim. The computer package ATLAS-ti will be used to manage the data. Data will be analysed according to the constant comparison method through thematic coding of the data [37]. Coding will take place using a combination of *a priori* themes (according to the aims of the qualitative study and the outcome measures of interest to the trial) and emergent themes. However, in addition, as participants in the qualitative sample would also have responses to the quantitative data collected for the trial, this will allow for the possibility of taking a mixed-methods approach to data integration, in which the two forms of data can be used in a complementary way [38].



For peer review only - http://bmjopen.bmj.com/site/about/guidelines.xhtml

(viii) Adverse event data

Adverse event data will be summarised descriptively by randomised arm.

**Economic evaluation**

The economic analysis will be performed using individual patient level data from the REFORM trial. The analytical approach will take the form of cost-effectiveness and cost-utility analyses. The cost-effectiveness approach will assess value for money in terms of cost per fall averted, and the cost-utility analysis will assess cost per quality adjusted life year (QALY) gained. The perspective for both analyses will be that of the UK NHS and Personal Social Services [39] as well as that of society. Discounting for future cost and health benefit will not be included considering the time frame for the trial is 12 months after randomisation. The year of pricing will be set as the mid-year of the trial.

Health benefits associated with the treatments will be measured in terms of both estimates of the mean number of falls, corresponding to the main outcome of the trial, and mean QALYs, which is defined as a year lived with full health. The EQ-5D [40] will be used to elicit patient utility values at different points in time and used to calculate QALYs for each patient using the area under the curve [41]. These utility values are used as 'quality adjustment' for each patient's survival time.

Mean within-trial estimates of cost and health benefits will be estimated using the regression approach to allow for the correlation between costs and effects as well as adjusting for covariates. This analysis will also account for skewness and censoring associated with time to event and cost data [42-44]. The result will be presented as incremental cost-effectiveness ratio (ICER) where the difference of mean cost estimates between two arms are divided by



For peer review only - http://bmjopen.bmj.com/site/about/guidelines.xhtml

the difference of mean health benefit between two arms. The Net Monetary Benefit (NMB) will be also presented [45]. The NMB provides an estimation of the gain (or loss) in resources of investing in a particular intervention when those resources might be used elsewhere [46].

The uncertainty surrounding the decision to accept a treatment as the most cost-effective will be explored in cost-effectiveness acceptability curves (CEAC) [47]. These curves depict the probability of accepting a treatment as being cost-effective for a large range of willingness to pay values for an extra unit of health benefit. Sensitivity analysis will be conducted to explore the impact of underlying assumptions of the model and the range of unit costs on the cost-effectiveness results.

Despite the careful design of any trial the presence of missing data is unavoidable. The reasons for the presence of incomplete data are multiple. Similarly there are different methods to handle its analysis [48]. The pattern of missing data for the economic analysis will be examined and described. The Multiple Imputation (MI) process has been recommended as the appropriate method to address the uncertainty in the results of economic evaluation due to missing data. Therefore the base case analysis will be based on the MI, whilst the complete case dataset will be used for the sensitivity analysis.

The main outcome of the REFORM trial, fall reduction, could be regarded as an intermediate outcome to achieve the final target - the reduction in fracture. However, due to the restriction in the length of follow-up, the long-term effect of decreasing the number of fractures, might not be observed within the timeframe of the current trial. Therefore a further analysis is


For peer review only - http://bmjopen.bmj.com/site/about/guidelines.xhtml
For peer review only

planned to model the possible long-term impact of the trial assuming that a falls reduction should also lead to a fracture reduction.

A decision analytic model approach will be adopted to perform such a task. The perspective will be the UK NHS and Personal Social Services and the time horizon for this analysis will be a life-time horizon. Life-time horizon refers to following up every single participant in a hypothetical cohort until the last participant dies. The hypothetical cohort will be constructed, based on the characteristics of the trial population, to estimate the QALY yield and cost saving of the long-term effect of the intervention. The model parameters which are not collected in the trial will be extracted from the existing literature. The model outputs will be the estimated expected mean costs, effectiveness, and QALYs associated with each alternative treatment. Estimated total costs and outcomes will be discounted properly according to the current guidance of health technology appraisal [39].

Uncertainty regarding cost-effectiveness will be evaluated using probabilistic sensitivity analysis, where inputs into the analysis are defined as probability distributions which reflect uncertainty. The uncertainty surrounding the decision to adopt a given treatment option as a cost-effective treatment at different levels of willingness to pay will be represented in acceptability curves. The impact of assumptions undertaken in the analysis regarding the evidence over parameters or relating to the decision model (such as extrapolation) will be evaluated in a sensitivity analysis, if possible.



For peer review only - http://bmjopen.bmj.com/site/about/guidelines.xhtml

## ETHICS AND DISSEMINATION

### Ethics

The study (including the original application and subsequent amendments) has been approved by the NRES East of England - Cambridge East Research Ethics Committee and Galway Research Ethics Committee, Republic of Ireland. It has also been approved by the National Health Service (NHS) research and development (R&D) committee responsible for each participating trust as well as the University of York, Department of Health Sciences Research Governance Committee. All participants will give written informed consent prior to entry to the study.

### Dissemination

Results of the study will be disseminated through, national and international research conferences and in articles published in international peer-reviewed journals.

## DISCUSSION

This study uses the cohort randomised controlled trial (cRCT) design. Existing trial designs have reported issues around recruitment, ethics, patient preferences and treatment comparisons [49]. We chose this approach to find out whether the cRCT design can overcome these issues and to test its feasibility. We anticipate that using this design will offer the following advantages. First, we expect that recruitment rates will be enhanced. Some participants enrolled in the cohort will be immediately eligible for the main trial and can be randomised straight away. Others, however, will become eligible over time due to the fact that they have subsequently had a fall. If an alternative design had been chosen then these patients could not have been included in the study and would have been lost. Second, in this study participants are receiving routine podiatry care outside of the trial and apart from



For peer review only - http://bmjopen.bmj.com/site/about/guidelines.xhtml

altruistic reasons the only incentive to take part in the study is the possibility of receiving the extra package of podiatry care. If a pragmatic design had been used, then the control patients would have been notified of their group allocation, and this may have resulted in a higher attrition rate. If this had occurred then there is the possibility that selection bias could be introduced. In addition to this, participants may also either knowingly or unknowingly bias the trial by reporting the number of falls they have had less conscientiously than those allocated to the intervention arm. In the cRCT design, participants are only notified of their allocation to the intervention group. Control participants are only aware of their participation in the study as a whole, thereby minimising the possibility of introducing attrition and reporting bias. Third, we expect that using the cRCT design will help minimise post-randomisation attrition rates. Unfortunately, many trials lose participants, and unless this occurs in a random manner, there is the possibility of selection bias being introduced. Many participants are lost in the early stages of a study e.g. participants change their mind. Using the cRCT design, which effectively has a run-in period, allows the majority of patients to withdraw from the study prior to randomisation, thereby minimising post-randomisation attrition rates. Finally, one key feature of the cRCT design is the capacity to undertake multiple randomised controlled trials over time. Once this cohort is set up, we will apply for funding to undertake further trials using the participants in this study. This is not only a quick and cost-effective strategy to identifying trial participants but has the added advantage of reducing heterogeneity of trial populations enabling synthesis of trial data and indirect comparisons between trial treatments.

Falls in older people are a major health problem. Podiatry may have a role in preventing falls as there is some evidence to suggest that foot problems and inappropriate footwear may increase the risk of falling. A recent trial by Spink *et al* [10, 26] has demonstrated that a



For peer review only - http://bmjopen.bmj.com/site/about/guidelines.xhtml

podiatric intervention consisting of: footwear assessment and provision; foot orthotic device and a home-based foot and ankle exercise programme can reduce falls in patients aged over 65 years of age with disabling foot pain. As far as we are aware, such a multifaceted podiatry intervention has not been evaluated in a UK and Republic of Ireland setting. The REFORM trial aims to evaluate the clinical and cost-effectiveness of such a package of care in patients over the age of 65 years who have an increased risk of falling.

## TRIAL STATUS

Recruitment and follow up are in progress. Recruitment to the study began in October 2012 and will continue until approximately spring 2015. Patients will continue to be followed-up until approximately spring 2016.

For peer review only



For peer review only - http://bmjopen.bmj.com/site/about/guidelines.xhtml

**AUTHORS' CONTRIBUTIONS**

DT and SC

wrote the original protocol. SC, JA, CH, RH, AMK, SL, CM, HBM, ACR, WV JW and DT were applicants on the NIHR HTA funding application they and BCM, CF, KH, LL, SR have helped to further refine the protocol and have made substantial contributions to the design of the project. All authors read and approved the final manuscript. We would like to thank the Trial Steering/Data Monitoring and Ethics Committee for their input and advice with the study.

**COMPETING INTERESTS**

The authors declare that they have no competing interests.

**ACKNOWLEDGEMENTS**

This study is funded by the National Institute for Health Research (NIHR) Health Technology Assessment (HTA) Programme (Programme grant number 09/77/01). The views and opinions expressed herein are those of the authors and do not necessarily reflect those of the Department of Health. The University of York is the study sponsor and is legal responsibility for the initiation and management of the study: Sponsor representative Mrs Sue Final, Research Innovation Office York Science Park Heslington, York UK YO10 5DG. Lamb was supported by the National Institute of Health Research (NIHR) CLAHRC, Oxford Health NHS Trust and the NIHR BRU, Nuffield Orthopaedic Centre, University of Oxford. HBM is currently a National Health and Medical Research Council of Australia Senior Research Fellow (ID: 1020925). The research team would like to thank the independent members of the Trial Steering/Data Monitoring and Ethics Committee Professor Roger



For peer review only - http://bmjopen.bmj.com/site/about/guidelines.xhtml

Francis, Dr Sara Brookes, Dr Margaret May, Professor Chris Nester and Dr Ian Mathieson for their advice, overseeing the study and reviewing adverse event data.

**Data sharing:**

No additional data available.

**Figure Legends**

Figure 1 Formthotic orthotic device (colour for on-line version/monochrome for other format)

Figure 2 X-line orthotic device (colour for on-line version/monochrome for other format)

For peer review only



For peer review only - http://bmjopen.bmj.com/site/about/guidelines.xhtml

## REFERENCES

1. Iglesias CP, Manca A, Torgerson DJ. The health-related quality of life and cost implications of falls in elderly women. Osteoporos Int 2009;20(6):869-78 doi: 10.1007/s00198-008-0753-5 [published Online First: 10 October 2008].

2. Department of Health (DH) 2001. National Service Framework for Older People: London Department of Health. Available at: https://www.gov.uk/government/publications/quality-standards-for-care-services-for-older-people (accessed 20/10/2014)

3. Lord S, Sherrington C, Menz H, et al. Falls in older people: Risk factors and strategies for prevention. Second edition, Cambridge, UK:Cambridge University Press 2007.

4. Dunn JE, Link CL, Felson DT, et al. Prevalence of Foot and Ankle Conditions in a Multiethnic Community Sample of Older Adults. Am J Epidemiol 2004;159(5):491-498

5. Benvenuti F, Ferrucci L, Guralnik J, et al. Foot pain and disability in older persons: an epidemiologic survey. J Am Geriatr Soc. 1995;43(5):479-84.

6. Menz HB, Morris ME, Lord SR. Foot and Ankle Characteristics Associated With Impaired Balance and Functional Ability in Older People. J Gerontol A Biol Sci Med Sci. 2005 Dec;60(12):1546-1552

7. Barr EL, Browning C, Lord SR, et al. Foot and leg problems are important determinants of functional status in community dwelling older people. Disabil Rehabil 2005;27(16):917-23

8. Gillespie LD, Robertson MC, Gillespie WJ, et al. Interventions for preventing falls in older people living in the community. Cochrane Database Syst Rev 2009;2(CD007146)

9. Cameron ID, Murray GR, Gillespie LD, et al. Interventions for preventing falls in older people in nursing care facilities and hospitals. Cochrane Database Syst Rev 2010;1(1)



For peer review only - http://bmjopen.bmj.com/site/about/guidelines.xhtml

10. Spink MJ, Menz HB, Fotoohabadi MR, et al. Effectiveness of a multifaceted podiatry intervention to prevent falls in community dwelling older people with disabling foot pain: randomised controlled trial. BMJ 2011;342

11. Menz H, Morris M, Lord S. Foot and ankle risk factors for falls in older people: A prospective study. J Gerontol A Biol Sci Med Sci 2006;61(8):866 - 70

12. Mickle KJ, Munro BJ, Lord SR, et al. Foot pain, plantar pressures, and falls in older people: a prospective study. J Am Geriatr Soc 2010;58(10):1936-40

13. Mickle KJ, Munro BJ, Lord SR, et al. ISB Clinical Biomechanics Award 2009: toe weakness and deformity increase the risk of falls in older people. Clin Biomech (Bristol, Avon) 2009;24(10):787-91

14. Menant JC, Steele JR, Menz HB, et al. Optimizing footwear for older people at risk of falls. J Rehabil Res Dev 2008;45(8):1167 - 81

15. Hatton AL, Rome K, Dixon J, et al. Footwear interventions: a review of their sensorimotor and mechanical effects on balance performance and gait in older adults. J Am Podiatr Med Assoc 2013;103(6):516-33

16. Koepsell T, Wolf M, Buchner D, et al. Footwear style and risk of falls in older adults. J Am Geriatr Soc 2004;52(9):1495 - 501

17. Kelsey JL, Procter-Gray E, Nguyen U-SD, et al;. Footwear and falls in the home among older individuals in the MOBILIZE Boston study. Footwear science 2010;2(3):123 - 29

18. Menz HB, Morris ME, Lord SR. Footwear characteristics and risk of indoor and outdoor falls in older people. Gerontology 2006;52(3):174 - 80

19. Deandrea S, Lucenteforte E, Bravi F, et al. Risk Factors for Falls in Community-dwelling Older People. A Systematic Review and Meta-analysis. Epidemiology 2010;21(5):658-668

20.Society AG. Guideline for the prevention of falls in older persons. J Am Geriatr Soc 2001;49(5):664 - 72



For peer review only - http://bmjopen.bmj.com/site/about/guidelines.xhtml

21. Australian Commission on Safety and Quality in Health Care. Preventing falls and harm from falls in older people: best practice guidelines for Australian community care. Australian Commission on Safety and Quality in Health Care, 2009. Available at http://www.safetyandquality.gov.au/publications/preventing-falls-and-harm-from-falls-in-older-people-best-practice-guidelines-for-australian-hospitals-2009/ (accessed 20/10/2014)

22. Balanowski K, Flynn L. Effect of painful keratoses debridement on foot pain, balance and function in older adults. Gait Posture 2005;22(4):302 - 07

23. Hijmans JM, Geertzen JHB, Dijkstra PU, et al. A systematic review of the effects of shoes and other ankle or foot appliances on balance in older people and people with peripheral nervous system disorders. Gait Posture 2007;25(2):316-23 doi: http://dx.doi.org/10.1016/j.gaitpost.2006.03.010 [published Online First: 9 May 2006]

24. Kobayashi R, Hosoda M, Minematsu A, et al. Effects of toe grasp training for the aged on spontaneous postural sway. J Phys Ther Sci 1999;11(1):31 - 34

25. Schwenk M, Jordan ED, Honarvararaghi B, et al. Effectiveness of foot and ankle exercise programs on reducing the risk of falling in older adults: A systematic review and meta-analysis of randomized controlled trials. J Am Podiatr Med Assoc 2013;103(6):534-47

26. Spink MJ, Menz HB, Lord SR. Efficacy of a multifaceted podiatry intervention to improve balance and prevent falls in older people: study protocol for a randomised trial. BMC Geriatr 2008;8(1):30

27. Sheikh JI, Yesavage JA. Geriatric Depression Scale (GDS): Recent evidence and development of a shorter version. In Clinical Gerontology: A Guide to Assessment and Intervention. Edited by Brink TL. NY, NY Haworth Press 1986:165-173.

28. Brink TL, Yesavage JA, Lum O, et al. Screening tests for geriatric depression. Clin Gerontol 1982;1(1):37 - 43



For peer review only - http://bmjopen.bmj.com/site/about/guidelines.xhtml

29. Pighills AC, Torgerson DJ, Sheldon TA, et al. Environmental assessment and modification to prevent falls in older people. J Am Geriatr Soc 2011;59(1):26 - 33

30. Vernon W. The Healthy Footwear Guide. Podiatry Now 2009;5:35

31. Lamb S, Jorstad-Stein EC, Hauer K, et al. Development of a common outcome data set for fall injury prevention trials: the Prevention of Falls Network Europe consensus. J Am Geriatr Soc 2005;53(9):1618-22

32. Kind P. The EuroQoL instrument: an index of health-related quality of life. Quality of life and pharmacoeconomics in clinical trials 1996;2:191 - 201

33. Kempen GI, Yardley L, Van Haastregt JC, et al. The Short FES-I: a shortened version of the falls efficacy scale-international to assess fear of falling. Age Ageing 2008;37(1):45 - 50

34. J Schuling, R de Haan, M Limburg et al. The Frenchay Activities Index. Assessment of functional status in stroke patients. Stroke 1993;24:1173 – 77.

35. Patton MQ. Qualitative Evaluation and Research Methods. 2nd edition: Newbury Park, CA: Sage 1990

36. Robertson MC, Campbell AJ, Herbison P. Statistical Analysis of Efficacy in Falls Prevention Trials. J Gerontol A Biol Sci Med Sci 2005;60(4):530-534

37. Silverman D. Interpreting Qualitative Data: Methods for Analysing Talk, Text and Interaction: Methods for Analyzing Talk, Text and Interaction. London, UK: Sage 1993.

38. Adamson J. Combined qualitative and quantitative designs. In: Bowling A, Ebrahim S eds. Handbook of health research methods: Investigation, measurement and analysis. Maidenhead UK: Open University Press 2005:230-245.

39. National Institute for Health and Care Excellence. Guide to the methods of technology apraisal: London: NICE, 2013. http://www.nice.org.uk/article/pmg9/chapter/Foreword http://www.nice.org.uk/article/PMG9/chapter/Foreword (accessed October 2014).

40. Brooks R. EuroQol: the current state of play. Health policy 1996;37(1):53 - 72

For peer review only



For peer review only - http://bmjopen.bmj.com/site/about/guidelines.xhtml

41. Matthews J, Altman DG, Campbell M, et al. Analysis of serial measurements in medical research. BMJ 1990;300(6719):230

42. Nixon RM, Thompson SG. Methods for incorporating covariate adjustment, subgroup analysis and between-centre differences into cost-effectiveness evaluations. Health Econ 2005;14(12):1217 - 29

43. O'Hagan A, Stevens JW, Montmartin J. Bayesian cost-effectiveness analysis from clinical trial data. Stat Med 2001;20(5):733-53

44. Willan AR, Lin D, Manca A. Regression methods for cost-effectiveness analysis with censored data. Stat Med 2005;24(1):131 - 45

45. Stinnett AA, Mullahy J. Net health benefits: a new framework for the analysis of uncertainty in cost-effectiveness analysis. Med Decis Making 1998;18(Suppl 2):S68-80

46. Claxton K. Exploring uncertainty in cost-effectiveness analysis. Pharmacoeconomics 2008;26(9):781 - 98

47. Van Hout BA, Al MJ, Gordon GS, et al. Costs, effects and C/E-ratios alongside a clinical trial. Health econ 1994;3(5):309 - 19

48. Manca A, Palmer S. Handling missing data in patient-level cost-effectiveness analysis alongside randomised clinical trials. Appl Health Econ Health Policy 2005;4(2):65 - 75

49. Relton C, Torgerson D, O'Cathain A, et al. Rethinking pragmatic randomised controlled trials: introducing the "cohort multiple randomised controlled trial" design. BMJ 2010;340

For peer review only

# The REFORM study protocol: a cohort randomised controlled trial of a multifaceted podiatry intervention for the prevention of falls in older people

Sarah Cockayne[1§], Joy Adamson[1], Belen Corbacho Martin[1], Caroline Fairhurst[1], Catherine Hewitt[1], Kate Hicks[1], Robin Hull[2], Anne-Maree Kenan[3,4], Sarah E Lamb[5], Lorraine Loughrey[1,3], Caroline McIntosh[6], Hylton B Menz[7], Anthony C Redmond[3, 4], Sara Rodgers[1], Wesley Vernon[8], Judith Watson[1], David Torgerson[1] on behalf of the REFORM study

[1]York Trials Unit, Department of Health Sciences, University of York, YO10 5DD, UK

[2] Podiatry Services, Harrogate and District NHS Foundation Trust, Harrogate District Hospital, Lancaster Park Road, UK

[3]NIHR Leeds Musculoskeletal Biomedical Research Unit, Chapel Allerton Hospital, Leeds, UK

[4]Leeds Institute of Rheumatology and Musculoskeletal Medicine, University of Leeds

[5]Nuffield Department of Orthopaedics, Rheumatology and Musculoskeletal Sciences, Kadoorie Critical Care Research Centre, John Radcliffe Hospital, University of Oxford, UK

[6]National University of Ireland, Galway, Republic of Ireland

[7]Lower Extremity and Gait Studies Program, Faculty of Health Sciences, La Trobe University, Bundoora 3086, Victoria, Australia

[8]Podiatry Services, Sheffield Teaching Hospitals NHS Foundation Trust, Jordanthorpe Health Centre, 1 Dyche Close, Sheffield, UK

[§]Corresponding author

Email addresses:



For peer review only - http://bmjopen.bmj.com/site/about/guidelines.xhtml
For peer review only

§SC: sarah.cockayne@york.ac.uk telephone number 01904 321736

JA: joy.adamson@york.ac.uk

BC: belen.corbacho@york.ac.uk

CF: caroline.fairhurst@york.ac.uk

CH: catherine.hewitt@york.ac.uk

KH: kate.hicks@york.ac.uk

RH: Robin.Hull@hdft.nhs.uk

AMK: a.keenan@leeds.ac.uk

SEL: sarah.lamb@ndorms.ox.ac.uk

LL: L.R.Loughrey@leeds.ac.uk

CM: caroline.mcintosh@nuigalway.ie

HBM: h.menz@latrobe.edu.au

ACR: a.redmond@leeds.ac.uk

SR: sara.rodgers@york.ac.uk

WV: wesley.vernon@nhs.net

JW: jude.watson@york.ac.uk

DT: david.torgerson@york.ac.uk

**Key words**

Cohort randomised controlled trial, multifaceted podiatry intervention, prevention of falls.

Word count 6919



For peer review only - http://bmjopen.bmj.com/site/about/guidelines.xhtml

**ABSTRACT**

**Introduction**

Falls and fall related injuries are a serious cause of morbidity and cost to society. Foot problems and inappropriate footwear may increase the risk of falls; therefore podiatric interventions may play a role in reducing falls. Two Cochrane systematic reviews identified only one study of a podiatry intervention aimed to reduce falls, which was undertaken in Australia. The REFORM trial aims to evaluate the clinical and cost-effectiveness of a multifaceted podiatry intervention in reducing falls in people aged 65 years and over in a UK and Irish setting.

**Methods and analysis**

This multicentre, cohort randomised controlled trial will recruit 2,600 participants from routine podiatry clinics in the UK and Ireland to the REFORM cohort. In order to detect a 10% point reduction in falls from 50% to 40%, with 80% power 890 participants will be randomised to receive routine podiatry care and a falls prevention leaflet or routine podiatry care, a falls prevention leaflet and a multifaceted podiatry intervention. The primary outcome is rate of falls (falls/person/time) over 12 months assessed by patient self-report falls diary. Secondary self-report outcome measures include: the proportion of single and multiple fallers and time to first fall over a 12 month period; Short Falls Efficacy Scale – International; fear of falling in the past four weeks; Frenchay Activities Index; fracture rate; Geriatric Depression Scale; EQ-5D 3-L; health service utilisation at 6 and 12 months. A qualitative study will examine the acceptability of the package of care to participants and podiatrists.

**Ethics and dissemination**




For peer review only - http://bmjopen.bmj.com/site/about/guidelines.xhtml

The trial has received a favourable opinion from the East of England - Cambridge East Research Ethics Committee and Galway Research Ethics Committee. The trial results will be published in peer-reviewed journals and at conference presentations.

**Trial registration number** Current Controlled Trials ISRCTN68240461assigned 01/07/2011

**Strengths and limitations of this study**

- This study is the first UK study evaluating the clinical and cost-effectiveness of podiatric care combined with footwear advice and provision (if required), orthotic inserts and foot and ankle exercises for falls prevention in people over 65 years of age.
- This study uses the novel cohort randomised controlled trial design.
- The study uses an unblinded, patient self-report primary outcome measure.
- It will not be possible to determine the clinical and cost-effectiveness of this intervention in patients who fall but do not receive routine podiatry care.

For peer review only



For peer review only - http://bmjopen.bmj.com/site/about/guidelines.xhtml

## BACKGROUND

Falls and fall related injuries are a serious cause of morbidity and cost to society [1], a burden which will increase with an ageing population. The National Service Framework (NSF) for Older People [2] recognises the importance of fall related injuries, and calls for health improvement plans to be devised that will reduce this burden.

It is well recognised that falls result from interactions between environmental hazards, a variety of medical conditions and physiological risk factors [3]. Foot problems may also increase the risk of falls.  Foot problems affect one in three community dwelling people over the age of 65 years [4] and are associated with reduced walking speed and difficulty in performing activities of daily living [5-7].

There have been two relevant Cochrane reviews on falls prevention, one relating to falls in community dwelling older people [8] and one focusing on falls in hospitals and aged care facilities [9]. At the time of designing the study neither identified any randomised controlled trials (RCTs) focusing on podiatry-related interventions.  A subsequent update has identified one trial of a podiatry based intervention for the prevention of falls [10] which related to the Australian health care system

There is some evidence to suggest that foot problems are associated with an increased risk of falling.  Menz *et al's* [6] prospective study of 176 older people identified ankle flexibility, toe plantarflexor strength and plantar sensation as significant and independent predictors of balance and functional test performance. These factors were later confirmed as predictors of falling during a 12 month follow up of that cohort, in which foot pain was also identified as a



For peer review only - http://bmjopen.bmj.com/site/about/guidelines.xhtml

For peer review only

predictor of falling [11]. Mickle *et al's* [12, 13] prospective study of 312 people over 60 years of age also found that fallers had significantly higher prevalence of foot pain, and displayed significantly less strength of the hallux and were more likely to have hallux valgus and lesser toe deformities.

Inappropriate footwear may also impair balance and increase the risk of falling. Footwear characteristics considered detrimental to balance include high heels, soft soles and inadequate slip resistance [14, 15]. Prospective studies have shown that walking barefoot or wearing only stockings inside the home and wearing shoes with an increased heel height and smaller contract area increases the risk of falling [16-18]. People who have a history of falling are at increased risk of falls. [19]

Given the emerging evidence that foot problems and inappropriate footwear increase the risk of falling, it has been suggested that podiatry may have a role to play in falls prevention, with several guidelines recommending that older people have their feet and footwear examined by a podiatrist [~~19~~, 20, 21].

Several studies have also suggested that some treatments provided by podiatrists, such as lesion debridement [2~~1~~2], foot orthoses [2~~2~~3], foot and ankle exercises [2~~3~~4, 2~~4~~5] and footwear advice may play a role in improving balance. Combining these therapies should allow for stability and improved function to be achieved at each level. Lesion debridement can improve function during gait if pain is reduced, exercise programmes focus on internal strengthening and flexibility, and appropriate footwear with orthotic devices can provide external support, improved kinaesthesia and improved function. A trial conducted in Australia in 305 community dwelling older people with disabling foot pain showed a 36%

For peer review only



For peer review only - http://bmjopen.bmj.com/site/about/guidelines.xhtml

reduction in falls for those receiving a multifaceted podiatry intervention, consisting of a foot orthosis, advice on footwear, subsidy for new footwear, a home based programme of foot and ankle exercises, a falls prevention education booklet and routine podiatry care for 12 months [10, 2~~5~~6]. The control group received only routine podiatry care for 12 months which consisted of toenail maintenance and scalpel debridement of hyperkeratotic lesions (corns and calluses). There have been no further large pragmatic trials looking at podiatric care combined with footwear advice, foot and ankle exercise and orthotic inserts for falls prevention, and there has been no assessment of whether such an intervention is economically viable within the UK health care setting. The REFORM (REducing Falls with ORthoses and a Multifaceted podiatry intervention) trial aims to address these issues. The main objectives are to investigate the clinical and cost-effectiveness of a multifaceted podiatry intervention for falls prevention and to assess views and experiences of the intervention and the trial process from the perspective of the participants and podiatrists.

For peer review only

## METHODS AND ANALYSIS

### Design

The REFORM study is a “cohort randomised controlled trial” (cRCT). Firstly we will recruit participants to the REFORM cohort and whilst this is being assembled, we will invite a selection of eligible participants from the site undertaking the pilot phase to take part in a REFORM pilot study. Once the pilot study is complete, we will then invite the remaining eligible participants to take part in the main REFORM trial. The REFORM main trial is a two arm, pragmatic, open, multicentre, randomised controlled trial with a 12 month follow-up.



For peer review only - http://bmjopen.bmj.com/site/about/guidelines.xhtml

**REFORM study objectives for the pilot phase**

(i) To demonstrate the feasibility of recruiting to the REFORM cohort

(ii) To develop and pilot the multifaceted podiatry intervention including a foot and ankle exercise programme, supplementary DVD and booklet with approximately 60 participants

(iii) To develop the podiatrist training package

(iv) To pilot the falls calendar and other patient data collection questionnaires

(v) To assess participants' views and experiences of the intervention and the trial process

(vi) Pilot, review and refine if necessary recruitment methodology for the main trial

~~Upon successful achievement of~~ All these objectives were achieved and the pilot has moved ~~we will~~ seamlessly ~~progress~~ into the main REFORM trial.

**REFORM study objectives for the main study**

This study will aim to address the following:

(i) To train the podiatrists to deliver the intervention

(ii) To examine the clinical and cost-effectiveness of the multifaceted podiatry intervention for falls prevention

(iii) To assess the podiatrists' views and experiences of the intervention and trial process

**Participants**

Participant recruitment

Participants will be recruited from NHS podiatry clinics based in either primary or secondary care in the UK and one international site in the Republic of Ireland. The rationale for



For peer review only - http://bmjopen.bmj.com/site/about/guidelines.xhtml

recruiting only from podiatry clinics is due to the logistical constraints imposed by the requirement for the control group to receive routine podiatry care. Whilst it would be possible to recruit from GP practices, many of the patients identified in this setting would not be receiving routine podiatry care, and NHS podiatry service managers indicted that they would not have the capacity to see large numbers of additional patients.

Podiatrists within the NHS Trust or clinic at the Republic of Ireland or the REFORM research podiatrist will undertake a search of either electronic or paper patient medical notes to identify potential participants for the study. Patients aged 65 years and over who are registered with the service and have attended routine podiatry services within the past six months of the search being undertaken will be identified and will be eligible for an invitation mailing. Patients who have attended high risk clinics, (for example diabetes clinics), or who live in a nursing home will be excluded from the invitation mail out. Sites will be requested where possible to screen out patients in the following groups: patients with a life expectancy of less than six months; patients known to have dementia, a neurodegenerative disorder, neuropathy, a lower limb amputation, or are chair or bed bound. All eligible patients will be sent an invitation pack (letter of invitation, participant information sheet, consent form, screening questionnaire and pre-paid envelope) asking whether they would like to participate in the REFORM study. Where the clinic has the capacity, potential participants attending routine podiatry clinics may be approached opportunistically. Where a multidisciplinary team exists, other healthcare professionals such as occupational therapists, falls practitioners and physiotherapists may support the opportunistic recruitment.

Participants wishing to take part in the REFORM study will be asked to return their completed consent form and screening questionnaire by post to the York Trials Unit (YTU).


For peer review only - http://bmjopen.bmj.com/site/about/guidelines.xhtml

Researchers at the YTU will assess the returned screening forms for participant eligibility. During the consenting stage, potential participants will be informed of the possibility of participating in other related studies, e.g. an associated qualitative study and they will be asked to indicate (by ticking a box on the consent form), if they would prefer not to be approached about these studies. Participants can withdraw from the study at any point. The reason for withdrawal will not have to be declared; however, if provided, this will be recorded. Data will be retained for all participants up to the date of withdrawal, unless a participant specifically requests their details be removed. All data returned to the York Trials Unit by participants will be held in accordance with the Data Protection Act 1998.

**Inclusion criteria for the REFORM cohort**

All eligible, consenting participants will be asked to complete a baseline questionnaire. Participants who return valid baseline data will be included in the REFORM cohort. Any participant reporting a score of 10 or more on the Geriatric Depression Scale [~~26~~, 27, 28] i.e. more severe depression, will be referred to their general practitioner (GP).

**Exclusion criteria for the REFORM cohort**

Participants will be ineligible for the REFORM cohort if they are under 65 years of age; report having neuropathy, dementia or other neurological condition such as Parkinson's disease, Alzheimer's, multiple sclerosis, Lou Gehrig's/amyotrophic lateral sclerosis or Huntington's disease; are unable to walk household distances (10 metres) without the help of a walk aid such as a Zimmer frame or walker or person to assist; have had a lower limb amputation; or are unwilling to attend their podiatry clinic.

**Inclusion criteria for the REFORM trial**



For peer review only - http://bmjopen.bmj.com/site/about/guidelines.xhtml

Participants will be eligible for the REFORM trial if they:

(i) have had one fall in the past 12 months; or one fall in the past 24 months requiring hospital attention; or report a fear of falling on their baseline questionnaire i.e. have worried about falling at least some of the time, in the past four weeks

(ii) are community dwelling

**Exclusion criteria for the REFORM Trial**

Participants who do not complete the baseline or run-in data collection instruments adequately, or who are unable to read or speak English will be excluded from the trial.

**Participants who do not wish to take part in the study**

Participants who do not wish to take part in the main study are not required to return any forms to the YTU. However, all participants in the pilot phase of the study who are sent an invitation pack will be given the opportunity to decline participation and if willing, provide some demographic information and reason for declining. This will provide us with sufficient information to document the reasons why participants do not wish to take part in the study and will allow us to compare decliners to those who are participating. The recruitment pack in the main mail out will not contain a decline form.

**Sample size**

The REFORM cohort

We propose to recruit up to 2,600 participants into the REFORM cohort to allow us to sample for the pilot trial and to allow cohort attrition before we sample for the main REFORM trial.



For peer review only - http://bmjopen.bmj.com/site/about/guidelines.xhtml

Internal pilot phase

Pilot trial: In order to pilot the intervention, a random sample of 60 participants will be selected from the REFORM cohort. Participants will be allocated equally to each of the two groups i.e. 30 participants per group. The allocation sequence will be computer generated by the YTU randomisation service and will be stratified by centre.

REFORM main trial

The REFORM trial is designed to detect a 10% point reduction in the number of people who fall over a 12 month period. Assuming this high risk group has an underlying risk of falling in a 12 month period of 50% [29~~8~~] then in order to observe a reduction to 40% with 80% power and a two-sided 5% significance level we would require 890 participants (445 in each group, allowing for a 10% loss to follow up).

**Randomisation**

Participants who fulfil the eligibility criteria for the REFORM trial and who have provided written consent to take part in the study will be eligible for randomisation. Randomisation will be carried out by the YTU secure remote computer randomisation service. Eight hundred and ninety participants will be randomly allocated in a 1:1 ratio to either the intervention or control group. If more than 890 eligible participants are recruited then we will randomly allocate the remaining participants in a 2:1 ratio in favour of the control group. This will allow us to increase the power of the study, without putting patients at any additional risk and without incurring additional costs. In order to allow for manageable case-loads at individual sites, the randomisation is by permuted blocks and stratified by centre. The YTU will write to the participant's GP informing them of study participation and to participants who are allocated to the intervention group.

For peer review only



For peer review only - http://bmjopen.bmj.com/site/about/guidelines.xhtml

**Blinding**

Due to the nature of the intervention, blinding of participants will not be feasible. Blinding of members of the study team who are actively involved in the administration of the study and may collect primary outcome falls data or undertake data queries on secondary outcomes, or the health economist will not be possible. Members of the study team responsible for data entry and the statistical analysis of the study will be kept blind to group allocation.

**Intervention group**

Participants allocated to receive the intervention will be seen by the podiatrist at the clinic they would normally attend for routine podiatry care, as soon as possible after randomisation. Participants are invited to attend two podiatry visits approximately two to four weeks apart, with further appointments if required in addition to their usual podiatry care.

(i) Footwear advice and provision

Participants' everyday footwear (indoor and outdoor footwear) will be assessed according to the following characteristics: appropriate size; method of fastening; height and width of the shoe's heel; thickness of outsole; heel counter stiffness; longitudinal sole rigidity; sole flexion point and tread pattern. Footwear will be deemed to be inappropriate if the shoes have any of the following characteristics: (i) heel height is greater than 4.5cm; (ii) no adjustable fixation of the upper; (iii) no heel counter or the heel counter can be depressed to greater than 45°; (iv) a fully worn/smooth/thin sole; (v) the shoe heel width is narrower than the participant's heel width by greater than or equal to 20%; or (vi) incorrect shoe size [10]. Participants with inappropriate footwear will be counselled regarding the hazardous footwear feature(s) identified during the assessment and will be advised on safer characteristics for future



For peer review only - http://bmjopen.bmj.com/site/about/guidelines.xhtml

footwear purchases. Where possible, footwear will be provided for participants whose footwear is deemed to be inappropriate. The podiatrist will order shoes directly from two companies participating in the Healthy Footwear Guide (HFG) scheme [2930]; DB shoes (Rushden, UK) or Hotter company (Skelmersdale, UK). As not all of the shoes manufactured by these companies fulfil the characteristics of a safe shoe, the trial team will assess the shoes and provide a list of suitable footwear from which participants can choose. In order to avoid incentivising patients to take part in the study, participants will only be told about footwear provision, if they are deemed to need new footwear. During the REFORM pilot trial we will explore the feasibility of making a footwear assessment, providing footwear advice and provision of footwear and if necessary we will revise the intervention if needed.

(ii) Foot orthoses

We originally planned to use the same orthotic device (Formthotics™, Foot Science International, Christchurch, NZ) (Figure 1) as our Australian collaborator [10, 265] as they had demonstrated that this device, commonly used in Australia, was acceptable to patients and was shown to be associated with a reduction in falls. However, during the setup period of the pilot phase of the REFORM trial, podiatrists at the recruiting sites reported difficulties when fitting these devices with the addition of posting (the application of wedges under the forefoot or rearfoot) and the ease of fitting in patient's current shoes. These sites frequently use the x-line® orthoses range (Healthystep, Mossley, UK) (Figure 2) as they are easy to modify and are easy to fit in patient's current shoes. During the pilot trial we will provide participants with both types of device to determine which device is most acceptable to a UK population. The Formthotics™ device will be issued as per the manufacturing guideline using the appropriate heating equipment and fitting procedure. The x-line® PressurePerfect/Standard device will be fitted and if indicated, modified to improve foot

For peer review only

posture as assessed in usual clinical practice by the individual clinician. If a participant already has insoles, whether shop bought or prescribed, the clinician will make a clinical judgement on the suitability of replacing their current insole with the trial insole. If the current insole is replaced with the trial insole then any current prescription/modifications maybe repeated, if applicable. We would consider the insole component of the intervention already addressed if the clinician felt it detrimental to replace their current insole with that of the trial insole. If the podiatrist feels that the patient requires more than what the current or trial insole can provide, or if they present with a musculoskeletal pathology, then a referral will be made in line with routine practice.

(iii) Home-based foot and ankle exercise programme

Participants will be prescribed a 30 minute home-based foot and ankle exercise programme which will be undertaken three times per week, indefinitely. The exercise programme is aimed at stretching and strengthening the muscles of the foot and ankle. The exercises will be based on the programme developed by Spink *et al* [10, 2~~5~~6] and will be adapted to make it suitable for a UK setting during the pilot phase of the trial. A summary of the individual exercises is given in Table 1. The exercises will be demonstrated by the podiatrist and will be supplemented by a DVD and an illustrated explanatory booklet. Participants will be assessed for competence and safety at the baseline and follow-up appointments.

(iv) Routine podiatry care

Routine podiatry care will continue to be given at separate podiatry appointments in accordance with participant's usual care. This will aim to reduce painful conditions such as corns and callouses, that have been found to be associated with an increased risk of falls.

For peer review only



For peer review only - http://bmjopen.bmj.com/site/about/guidelines.xhtml

**Table 1 Summary of the home based foot and ankle exercises**

| **Activity** | **Description** | **Dosage** | **Increments** |
|---|---|---|---|
| Ankle range of motion/ warm up | Sitting with the knee at 90 degrees. Lift the foot to clear the ground and then rotate the foot slowly in a clockwise direction and then an anti-clockwise. | 1x10 repetitions for each foot in each direction. | None. |
| Ankle inversion strength | Sitting upright, hip, knee and ankle at 90º. Invert foot against resistive exercise band. The band should be fixed at 90 degrees to the foot from an additional chair/table leg. | 3x10 repetitions for each foot. | Increase resistance strength of resistive exercise band. |
| Ankle eversion strength | Sitting upright, hip, knee and ankle at 90º. Evert foot against resistive exercise band The band should be fixed at 90 degrees to the foot from an additional chair/table leg | 3x10 repetitions for each foot. | Increase resistance strength of resistive exercise band. |
| Ankle dorsiflexion strength | Sitting, hip, knee and ankle at 90º.<br>Dorsiflex both feet to end range of motion and hold. Keep pulling feet up towards the body during the hold. | Hold feet in dorsiflexion for 3x10 seconds. | Increase repetitions up to maximum of 10. |
| Intrinsic strengthening, toe plantarflexion strength and toe stretch | Sitting, hip, knee and ankle at 90 º.<br>1) Use the therapy ball under the toes to stretch the toes. The rest of the foot should be plantargrade. Then curl and point the toes up and over the ball.<br>2) Use the therapy ball under the toes to stretch the toes. The rest of the foot should be plantargrade. With the heel on/close to the floor, curl the toes over the ball and attempt to pick up the ball with the toes. | 3 x10 repetitions for each exercise both feet. Have a 30 sec break between each repetition | Increase up to a maximum of 50 repetitions. |
| Ankle plantarflexion strength | From standing position, rise up onto toes of both feet and then slowly lower back down. | 3x10 repetitions. | Increase repetitions up to maximum of 50. |



For peer review only - http://bmjopen.bmj.com/site/about/guidelines.xhtml

| | | | |
|---|---|---|---|
| | Just before the heels contact the floor, rise back up onto the toes. | | |
| Calf stretch | Facing a wall and using hands on the wall for balance, step one foot in front of the other keeping feet hip width apart and hips, knees and feet facing the wall. Bend the knee closest to the wall and keep the back leg straight. Keep both heels in contact with the floor. | Hold stretch for 3x20 seconds on each leg. | Increase the stride length and forward lean to increase the stretch |
| Proprioception/balance training | From a standing position and holding on to a work surface/chair/wall for support, stand on one leg. Repeat on the other side | Hold for 30 seconds. Repeat 3 repetitions. | Increase slowly to hold for 1 min per repetition. If competent, rise up on to toes on the one supporting leg: 3 x 10 repetitions. |

(vi) Falls prevention leaflet and trial newsletter

Participants will receive a copy of the latest falls prevention advice leaflet produced by Age UK (the current version 'Staying steady' was printed in June 2010). This leaflet will be sent to the participant in the post with their baseline questionnaire.

Participants will be sent a site and group specific newsletter at three and 12 months informing them about progress of the study. The intervention group's newsletter will include a section about the foot and ankle exercises in order to aid compliance. All participants will receive £5 in recognition of their participation and to offset any incidental expenses associated with completing the questionnaires.



For peer review only - http://bmjopen.bmj.com/site/about/guidelines.xhtml

**Control group**

Participants allocated to the control group will receive the same falls prevention advice leaflet produced by Age UK. This leaflet will be sent to the participant in the post with their baseline questionnaire. Participants will continue to receive routine podiatry and GP access in accordance with their usual care. Participants will also be sent a site and group-specific newsletter at three and 12 months. All participants will receive £5 in recognition of their participation and to offset any incidental expenses associated with completing the questionnaires.

**Primary outcome measure for the REFORM trial**

The primary outcome is the rate of falls (i.e. falls/person/time) where a fall is defined as "an unexpected event in which the participant comes to rest on the ground, floor, or lower level" [3~~0~~1]. Data will be collected via participant self-reported monthly falls calendars throughout the 12 months following randomisation. Participants will be asked to record each day whether or not they had any fall. Participants who do not return their falls calendar within one week of the due date will be telephoned by the YTU to obtain the missing data. Participants will also be given a Freephone number to report any falls as soon as possible after the fall. Information collected includes: date and location of fall; reason for fall; any injuries sustained (e.g. a superficial wound or a broken bone); hospital admissions; footwear worn at the time of the fall; and if the patient was wearing an orthotic or using a walking aid. As we are collecting falls data at six and 12 months follow up, these data will be used for those participants who do not return their monthly falls calendar.



For peer review only - http://bmjopen.bmj.com/site/about/guidelines.xhtml

For peer review only

**REFORM trial secondary outcome measures**

All secondary outcomes are self-reported by the patient and collected by questionnaire at baseline, six and 12 months, or by monthly falls calendars. Secondary outcomes include: proportion of fallers (single and multiple); patient reported time to first fall during follow-up; health related quality of life as measured by the EQ-5D [3~~0~~2]; fear of falling as measured by the question, “During the past 4 weeks have you worried about having a fall?”, fear of falling as measured by the Short Falls Efficacy Scale – International (FES-I) [3~~1~~3], activities of daily living as measured by the Frenchay Activities Index (FAI) [3~~2~~4], fracture rate, health service utilisation and depression as measured by the short form Geriatric Depression Scale (GDS) [2~~7~~8].

**Nested qualitative study**

A qualitative evaluation will be carried out to examine the acceptability of the intervention as a whole package of care, to both the trial participants and the podiatry practitioners. Trial participants from the pilot phase of the study who are receiving the intervention will be asked about their experience of the intervention. Particular attention will be paid to the acceptability and compliance with the foot orthosis, exercise programme, podiatry service and footwear advice/purchase and how the intervention fits into the individual’s wider experience of balance problems within their everyday lives.

Ten to 15 participants will be purposively selected from the pilot phase of the trial (the first 60 participants randomised) to ensure a representative spread according to falls history, age and gender. This maximum variation sampling approach [3~~3~~5] will ensure a wide range of viewpoints are included in the data collection and analysis. Participants will be invited to attend a face-to-face interview or, if prefered, a telephone interview. Written informed

For peer review only



For peer review only - http://bmjopen.bmj.com/site/about/guidelines.xhtml

consent will be obtained from the participant prior to the interview. The semi-structured interviews will be conducted within approximately two weeks of receiving the six month follow-up questionnnaire. A follow-up telephone interview will be conducted within two weeks of receiving the 12 month follow-up questionnaire to discuss any changes (or not) recorded in the outcomes measured over the longer term follow-up period. All interviews will be conducted using a topic guide to ensure consistency across participants. However, the format will be flexible in order to allow participant-led data regarding what they constitute as important and/or successful in terms of treatment outcome.

We will interview a purposive sample of five to ten practitioners who are providing the podiatry services for the study regarding the way in which they have delivered the intervention, the ease of delivery, their confidence in the intervention, and their experience of being involved in the trial. Face-to-face or telephone interviews will be conducted approximately three months into the trial process. Informed consent will be obtained prior to the interview being conducted.

**Adverse events**

Details of any adverse events reported to the YTU either directly by the participant or by a member of the research team at the recruiting site will be recorded.

This study will report details of any serious adverse events (SAEs) that are required to be reported to the Research Ethics Committee (REC) under the current terms of the Standard Operating Procedures for RECs.

A SAE is defined as any untoward occurrence that:



For peer review only - http://bmjopen.bmj.com/site/about/guidelines.xhtml

For peer review only

(i) Results in death;

(ii) Is life threatening;

(iii) Requires hospitalisation or prolongation of existing hospitalisation;

(iv) Results in persistent or significant disability or incapacity;

(v) Consists of a congenital anomaly or birth defect, or

(vi) Is otherwise considered medically significant by the investigator.

An event is defined as 'related' if the event was due to the administration of any research procedure. An 'unexpected event' is defined as a type of event not listed in the protocol as an expected occurrence. Expected events include: aches and pains in the lower limb lasting for longer than 48 hours; fall; new callus/corn formation, blisters, ulcers; skin irritation/injury including pressure sores and soft tissue injury.

In the context of this study, an occurrence of the type listed in (i) to (vi) will be reported as an SAE only if:

(i)

the event is suspected to be related to an aspect of the research procedures (e.g. wearing the orthotic, undertaking the exercise programme, completion of follow-up questionnaires, participation in feasibility or qualitative sub-studies, telephone contact), and;

(ii)

it is an unexpected occurrence. Hospitalisations, disabling / incapacitating / life-threatening conditions, falls and deaths are expected in the study population due to the age of the cohort, they will therefore only be reported as SAEs if they appear to be related to an aspect of taking part in the study.



For peer review only - http://bmjopen.bmj.com/site/about/guidelines.xhtml

**Other data collected**

Treatment details will be recorded by the podiatrist including: the number of podiatry visits; an eligibility checklist with details on relevant health conditions and test results; characteristics of current indoor and outdoor shoes; details relating to shoes ordered; details on the type and prescription of any current insole use; the type of insole issued/retained with any modifications made, and any amendments or advice given on the intervention due to safety reasons.

Information on adherence to the exercise, footwear advice and orthotic components of the intervention will be collected from participant self-reported questionnaire data at three, six and 12 months. Participants will be asked whether during the past month, they were wearing their orthotic 'all of the time', 'most of the time', 'some of the time', 'a little of the time' or 'none of the time'. Participants will also be asked, for the past month, typically how many times a week they did the exercises: none, one, two, three, or more than three times. Participants will be asked at 12 months if they were given footwear advice, and whether or not they followed any given advice.

**Statistical analysis**

There will be a single analysis at the end of the trial, conducted using STATA (StataCorp, 4905 Lakeway Drive, College Station, Texas 77845 USA). All analyses will be conducted on an intention to treat basis, i.e. including all randomised patients in the groups to which they were assigned. All tests will be two-sided at the 5% significance level. If more than 890 participants are recruited and unequal allocation is utilised, then analyses will be adjusted to take this into account.



For peer review only - http://bmjopen.bmj.com/site/about/guidelines.xhtml

(i) The REFORM cohort

Descriptive statistics will be presented for participants in the REFORM cohort.

(ii) REFORM trial

Participant baseline data will be summarised descriptively by randomised arm. No formal statistical comparisons will be undertaken. Continuous measures will be reported as means and standard deviations whilst the categorical data will be reported as counts and percentages.

(iii) Statistical analysis of the REFORM trial primary outcome

The number of falls per person will be analysed using a Poisson regression model adjusting for gender, age, centre and history of falling to estimate the difference in falls rate between the groups. If there is over dispersion, a negative binomial regression model adjusting for the same factors will be used [346]. Point estimates and their associated 95% confidence intervals will be provided.

(iv) Statistical analysis of the REFORM trial secondary outcomes

The proportion of fallers versus non-fallers in each group will be compared by logistic regression adjusting for gender, age, centre and history of falling. Odds ratios and their associated 95% confidence intervals will be provided. The proportion of multiple fallers versus single or non-fallers in each group will be compared over the 12 month trial period using logistic regression adjusting for gender, age, centre and history of falling.

The time to the first fall will be derived as the number of days from randomisation until the patient reports having a fall as detailed from the participant's falls calendar, falls telephone

For peer review only



For peer review only - http://bmjopen.bmj.com/site/about/guidelines.xhtml

data collection sheet or self-reported questionnaire. Participants who have not had a fall will be treated as censored at their date of trial exit, or date of last available assessment or 365 days/trial cessation, as appropriate. The proportion of patients yet to experience a fall will be summarised by a Kaplan Meier survival curve for each group. The time to first fall will be analysed by Cox Proportional Hazard regression adjusting for gender, age, centre and history of falling. Hazard ratios and their associated 95% confidence intervals will be provided. The proportional hazard assumption will be evaluated using Schoenfeld residuals. The median time to the first fall and its associated 95% confidence intervals will be estimated from this adjusted model.

Patients with a score of six or more on the short form Geriatric Depression Scale will be categorised as having depression. The proportion of people with depression in each group will be compared over the 12 month trial period using logistic regression adjusting for gender, age, centre and history of falling.

The following secondary outcomes: Short Falls Efficacy Scale - International, fear of falling in the past four weeks, and the Frenchay Activities Index will be treated as continuous data and will be measured at baseline, month six and month 12. A covariance pattern model incorporating all post randomisation time points will be used to compare the two groups on these outcomes adjusting for baseline score, gender, age, centre and history of falling. The correlation of observations within patients over time will be modelled and the model effects and their associated 95% confidence intervals will be provided.

The proportion of patients obtaining at least one fracture over the 12 month follow-up period will be compared by logistic regression adjusting for gender, age, centre and history of



For peer review only - http://bmjopen.bmj.com/site/about/guidelines.xhtml

For peer review only

falling. Odds ratios and their associated 95% confidence intervals will be provided. If there are patients that obtain multiple fractures in this time (from different events), then the proportion of patients obtaining multiple fractures versus single or no fractures in each group will be compared over the 12 month trial period using logistic regression adjusting for gender, age, centre and history of falling.

(v) Intervention adherence

Descriptive statistics will be used to summarise the adherence to the exercise and orthotic at three, six, and 12 months and adherence to following footwear advice. A Complier Average Causal Effect (CACE) analysis to assess the impact of compliance on treatment estimates will be considered.

(vi) Missing data

The amount of missing data will be reported for each randomised arm.

(vii) Qualitative analysis

All interviews will be audio recorded digitally and transcribed verbatim. The computer package ATLAS-ti will be used to manage the data. Data will be analysed according to the constant comparison method through thematic coding of the data [3~~5~~7]. Coding will take place using a combination of *a priori* themes (according to the aims of the qualitative study and the outcome measures of interest to the trial) and emergent themes. However, in addition, as participants in the qualitative sample would also have responses to the quantitative data collected for the trial, this will allow for the possibility of taking a mixed-methods approach to data integration, in which the two forms of data can be used in a complementary way [3~~6~~8].

For peer review only



For peer review only - http://bmjopen.bmj.com/site/about/guidelines.xhtml

(viii) Adverse event data

Adverse event data will be summarised descriptively by randomised arm.

**Economic evaluation**

The economic analysis will be performed using individual patient level data from the REFORM trial. The analytical approach will take the form of cost-effectiveness and cost-utility analyses. The cost-effectiveness approach will assess value for money in terms of cost per fall averted, and the cost-utility analysis will assess cost per quality adjusted life year (QALY) gained. The perspective for both analyses will be that of the UK NHS and Personal Social Services [379] as well as that of society. Discounting for future cost and health benefit will not be included considering the time frame for the trial is 12 months after randomisation. The year of pricing will be set as the mid-year of the trial.

Health benefits associated with the treatments will be measured in terms of both estimates of the mean number of falls, corresponding to the main outcome of the trial, and mean QALYs, which is defined as a year lived with full health. The EQ-5D [38 40] will be used to elicit patient utility values at different points in time and used to calculate QALYs for each patient using the area under the curve [3941]. These utility values are used as 'quality adjustment' for each patient's survival time.

Mean within-trial estimates of cost and health benefits will be estimated using the regression approach to allow for the correlation between costs and effects as well as adjusting for covariates. This analysis will also account for skewness and censoring associated with time to event and cost data [40-4242-44]. The result will be presented as incremental cost-



For peer review only - http://bmjopen.bmj.com/site/about/guidelines.xhtml

effectiveness ratio (ICER) where the difference of mean cost estimates between two arms are divided by the difference of mean health benefit between two arms. The Net Monetary Benefit (NMB) will be also presented [453]. The NMB provides an estimation of the gain (or loss) in resources of investing in a particular intervention when those resources might be used elsewhere [446].

The uncertainty surrounding the decision to accept a treatment as the most cost-effective will be explored in cost-effectiveness acceptability curves (CEAC) [457]. These curves depict the probability of accepting a treatment as being cost-effective for a large range of willingness to pay values for an extra unit of health benefit. Sensitivity analysis will be conducted to explore the impact of underlying assumptions of the model and the range of unit costs on the cost-effectiveness results.

Despite the careful design of any trial the presence of missing data is unavoidable. The reasons for the presence of incomplete data are multiple. Similarly there are different methods to handle its analysis [468]. The pattern of missing data for the economic analysis will be examined and described. The Multiple Imputation (MI) process has been recommended as the appropriate method to address the uncertainty in the results of economic evaluation due to missing data. Therefore the base case analysis will be based on the MI, whilst the complete case dataset will be used for the sensitivity analysis.

The main outcome of the REFORM trial, fall reduction, could be regarded as an intermediate outcome to achieve the final target - the reduction in fracture. However, due to the restriction in the length of follow-up, the long-term effect of decreasing the number of fractures, might not be observed within the timeframe of the current trial. Therefore a further analysis is



For peer review only - http://bmjopen.bmj.com/site/about/guidelines.xhtml

planned to model the possible long-term impact of the trial assuming that a falls reduction should also lead to a fracture reduction.

A decision analytic model approach will be adopted to perform such a task. The perspective will be the UK NHS and Personal Social Services and the time horizon for this analysis will be a life-time horizon. Life-time horizon refers to following up every single participant in a hypothetical cohort until the last participant dies. The hypothetical cohort will be constructed, based on the characteristics of the trial population, to estimate the QALY yield and cost saving of the long-term effect of the intervention. The model parameters which are not collected in the trial will be extracted from the existing literature. The model outputs will be the estimated expected mean costs, effectiveness, and QALYs associated with each alternative treatment. Estimated total costs and outcomes will be discounted properly according to the current guidance of health technology appraisal [379].

Uncertainty regarding cost-effectiveness will be evaluated using probabilistic sensitivity analysis, where inputs into the analysis are defined as probability distributions which reflect uncertainty. The uncertainty surrounding the decision to adopt a given treatment option as a cost-effective treatment at different levels of willingness to pay will be represented in acceptability curves. The impact of assumptions undertaken in the analysis regarding the evidence over parameters or relating to the decision model (such as extrapolation) will be evaluated in a sensitivity analysis, if possible.



For peer review only - http://bmjopen.bmj.com/site/about/guidelines.xhtml

## ETHICS AND DISSEMINATION

### Ethics

The study (including the original application and subsequent amendments) has been approved by the NRES East of England - Cambridge East Research Ethics Committee and Galway Research Ethics Committee, Republic of Ireland. It has also been approved by the National Health Service (NHS) research and development (R&D) committee responsible for each participating trust as well as the University of York, Department of Health Sciences Research Governance Committee. All participants will give written informed consent prior to entry to the study.

### Dissemination

Results of the study will be disseminated through, national and international research conferences and in articles published in international peer-reviewed journals.

## DISCUSSION

This study uses the cohort randomised controlled trial (cRCT) design. Existing trial designs have reported issues around recruitment, ethics, patient preferences and treatment comparisons [4~~7~~9]. We chose this approach to find out whether the cRCT design can overcome these issues and to test its feasibility. We anticipate that using this design will offer the following advantages. First, we expect that recruitment rates will be enhanced. Some participants enrolled in the cohort will be immediately eligible for the main trial and can be randomised straight away. Others, however, will become eligible over time due to the fact that they have subsequently had a fall. If an alternative design had been chosen then these patients could not have been included in the study and would have been lost. Second, in this study participants are receiving routine podiatry care outside of the trial and apart from



For peer review only - http://bmjopen.bmj.com/site/about/guidelines.xhtml

altruistic reasons the only incentive to take part in the study is the possibility of receiving the extra package of podiatry care. If a pragmatic design had been used, then the control patients would have been notified of their group allocation, and this may have resulted in a higher attrition rate. If this had occurred then there is the possibility that selection bias could be introduced. In addition to this, participants may also either knowingly or unknowingly bias the trial by reporting the number of falls they have had less conscientiously than those allocated to the intervention arm. In the cRCT design, participants are only notified of their allocation to the intervention group. Control participants are only aware of their participation in the study as a whole, thereby minimising the possibility of introducing attrition and reporting bias. Third, we expect that using the cRCT design will help minimise post-randomisation attrition rates. Unfortunately, many trials lose participants, and unless this occurs in a random manner, there is the possibility of selection bias being introduced. Many participants are lost in the early stages of a study e.g. participants change their mind. Using the cRCT design, which effectively has a run-in period, allows the majority of patients to withdraw from the study prior to randomisation, thereby minimising post-randomisation attrition rates. Finally, one key feature of the cRCT design is the capacity to undertake multiple randomised controlled trials over time. Once this cohort is set up, we will apply for funding to undertake further trials using the participants in this study. This is not only a quick and cost-effective strategy to identifying trial participants but has the added advantage of reducing heterogeneity of trial populations enabling synthesis of trial data and indirect comparisons between trial treatments.

Falls in older people are a major health problem. Podiatry may have a role in preventing falls as there is some evidence to suggest that foot problems and inappropriate footwear may increase the risk of falling. A recent trial by Spink *et al* [10, 2~~5~~6] has demonstrated that a

For peer review only



For peer review only - http://bmjopen.bmj.com/site/about/guidelines.xhtml

podiatric intervention consisting of: footwear assessment and provision; foot orthotic device and a home-based foot and ankle exercise programme can reduce falls in patients aged over 65 years of age with disabling foot pain. As far as we are aware, such a multifaceted podiatry intervention has not been evaluated in a UK and Republic of Ireland setting. The REFORM trial aims to evaluate the clinical and cost-effectiveness of such a package of care in patients over the age of 65 years who have an increased risk of falling.

## TRIAL STATUS

Recruitment and follow up are in progress. Recruitment to the study began in October 2012 and will continue until approximately spring 2015. Patients will continue to be followed-up until approximately spring 2016.

## COMPETING INTERESTS

The authors declare that they have no competing interests.

## AUTHORS' CONTRIBUTIONS

DT and SC wrote the original protocol. SC, JA, CH, RH, AMK, SL, CM, HBM, ACR, WV JW and DT were applicants on the NIHR HTA funding application they and BCM, CF, KH, LL, SR have helped to further refine the protocol and have made substantial contributions to the design of the project. All authors read and approved the final manuscript. We would like to thank the Trial Steering/Data Monitoring and Ethics Committee for their input and advice with the study.

## ACKNOWLEDGEMENTS

This study is funded by the National Institute for Health Research (NIHR)



For peer review only - http://bmjopen.bmj.com/site/about/guidelines.xhtml

For peer review only

Health Technology Assessment (HTA) Programme (Programme grant number 09/77/01). The views and opinions expressed herein are those of the authors and do not necessarily reflect those of the Department of Health. The University of York is the study sponsor and is legal responsibility for the initiation and management of the study: Sponsor representative Mrs Sue Final, Research Innovation Office York Science Park Heslington, York UK YO10 5DG. Lamb was supported by the National Institute of Health Research (NIHR) CLAHRC, Oxford Health NHS Trust and the NIHR BRU, Nuffield Orthopaedic Centre, University of Oxford. HBM is currently a National Health and Medical Research Council of Australia Senior Research Fellow (ID: 1020925). The research team would like to thank the independent members of the Trial Steering/Data Monitoring and Ethics Committee Professor Roger Francis, Dr Sara Brookes, Dr Margaret May, Professor Chris Nester and Dr Ian Mathieson for their advice, ~~and for~~ overseeing the study and reviewing adverse event data.

**Data sharing:**

No additional data available.

Figure 1 Formthotic orthotic device (colour for on-line version/monochrome for other format)

Figure 2 X-line orthotic device (colour for on-line version/monochrome for other format)

## REFERENCES

1. Iglesias CP, Manca A, Torgerson DJ. The health-related quality of life and cost implications of falls in elderly women. Osteoporos Int 2009;20(6):869-78 doi: 10.1007/s00198-008-0753-5 [published Online First: 10 October 2008].



For peer review only - http://bmjopen.bmj.com/site/about/guidelines.xhtml

2. Department of Health (DH) 2001. National Service Framework for Older People: London Department of Health. Available at: https://www.gov.uk/government/publications/quality-standards-for-care-services-for-older-people (accessed 20/10/2014)

3. Lord S, Sherrington C, Menz H, et al. Falls in older people: Risk factors and strategies for prevention. Second edition, Cambridge, UK:Cambridge University Press 2007.

4. Dunn JE, Link CL, Felson DT, et al. Prevalence of Foot and Ankle Conditions in a Multiethnic Community Sample of Older Adults. Am J Epidemiol 2004;159(5):491-498

5. Benvenuti F, Ferrucci L, Guralnik J, et al. Foot pain and disability in older persons: an epidemiologic survey. J Am Geriatr Soc. 1995;43(5):479-84.

6. Menz HB, Morris ME, Lord SR. Foot and Ankle Characteristics Associated With Impaired Balance and Functional Ability in Older People. J Gerontol A Biol Sci Med Sci. 2005 Dec;60(12):1546-1552

7. Barr EL, Browning C, Lord SR, et al. Foot and leg problems are important determinants of functional status in community dwelling older people. Disabil Rehabil 2005;27(16):917-23

8. Gillespie LD, Robertson MC, Gillespie WJ, et al. Interventions for preventing falls in older people living in the community. Cochrane Database Syst Rev 2009;2(CD007146)

9. Cameron ID, Murray GR, Gillespie LD, et al. Interventions for preventing falls in older people in nursing care facilities and hospitals. Cochrane Database Syst Rev 2010;1(1)

10. Spink MJ, Menz HB, Fotoohabadi MR, et al. Effectiveness of a multifaceted podiatry intervention to prevent falls in community dwelling older people with disabling foot pain: randomised controlled trial. BMJ 2011;342

11. Menz H, Morris M, Lord S. Foot and ankle risk factors for falls in older people: A prospective study. J Gerontol A Biol Sci Med Sci 2006;61(8):866 - 70

12. Mickle KJ, Munro BJ, Lord SR, et al. Foot pain, plantar pressures, and falls in older people: a prospective study. J Am Geriatr Soc 2010;58(10):1936-40



For peer review only - http://bmjopen.bmj.com/site/about/guidelines.xhtml

13. Mickle KJ, Munro BJ, Lord SR, et al. ISB Clinical Biomechanics Award 2009: toe weakness and deformity increase the risk of falls in older people. Clin Biomech (Bristol, Avon) 2009;24(10):787-91

14. Menant JC, Steele JR, Menz HB, et al. Optimizing footwear for older people at risk of falls. J Rehabil Res Dev 2008;45(8):1167 - 81

15. Hatton AL, Rome K, Dixon J, et al. Footwear interventions: a review of their sensorimotor and mechanical effects on balance performance and gait in older adults. J Am Podiatr Med Assoc 2013;103(6):516-33

16. Koepsell T, Wolf M, Buchner D, et al. Footwear style and risk of falls in older adults. J Am Geriatr Soc 2004;52(9):1495 - 501

17. Kelsey JL, Procter-Gray E, Nguyen U-SD, et al;. Footwear and falls in the home among older individuals in the MOBILIZE Boston study. Footwear science 2010;2(3):123 - 29

18. Menz HB, Morris ME, Lord SR. Footwear characteristics and risk of indoor and outdoor falls in older people. Gerontology 2006;52(3):174 - 80

19. Deandrea S, Lucenteforte E, Bravi F, et al. Risk Factors for Falls in Community-dwelling Older People. A Systematic Review and Meta-analysis. Epidemiology 2010;21(5):658-668

20.Society AG. Guideline for the prevention of falls in older persons. J Am Geriatr Soc 2001;49(5):664 - 72

21~~0~~. Australian Commission on Safety and Quality in Health Care. Preventing falls and harm from falls in older people: best practice guidelines for Australian community care. Australian Commission on Safety and Quality in Health Care, 2009. Available at http://www.safetyandquality.gov.au/publications/preventing-falls-and-harm-from-falls-in-older-people-best-practice-guidelines-for-australian-hospitals-2009/ (accessed 20/10/2014)



For peer review only - http://bmjopen.bmj.com/site/about/guidelines.xhtml

2~~1~~2. Balanowski K, Flynn L. Effect of painful keratoses debridement on foot pain, balance and function in older adults. Gait Posture 2005;22(4):302 - 07

2~~2~~3. Hijmans JM, Geertzen JHB, Dijkstra PU, et al. A systematic review of the effects of shoes and other ankle or foot appliances on balance in older people and people with peripheral nervous system disorders. Gait Posture 2007;25(2):316-23 doi: http://dx.doi.org/10.1016/j.gaitpost.2006.03.010 [published Online First: 9 May 2006]

2~~3~~4. Kobayashi R, Hosoda M, Minematsu A, et al. Effects of toe grasp training for the aged on spontaneous postural sway. J Phys Ther Sci 1999;11(1):31 - 34

2~~4~~5. Schwenk M, Jordan ED, Honarvararaghi B, et al. Effectiveness of foot and ankle exercise programs on reducing the risk of falling in older adults: A systematic review and meta-analysis of randomized controlled trials. J Am Podiatr Med Assoc 2013;103(6):534-47

26~~5~~. Spink MJ, Menz HB, Lord SR. Efficacy of a multifaceted podiatry intervention to improve balance and prevent falls in older people: study protocol for a randomised trial. BMC Geriatr 2008;8(1):30

27~~6~~. Sheikh JI, Yesavage JA. Geriatric Depression Scale (GDS): Recent evidence and development of a shorter version. In Clinical Gerontology: A Guide to Assessment and Intervention. Edited by Brink TL. NY, NY Haworth Press 1986:165-173.

28~~7~~. Brink TL, Yesavage JA, Lum O, et al. Screening tests for geriatric depression. Clin Gerontol 1982;1(1):37 - 43

29~~8~~. Pighills AC, Torgerson DJ, Sheldon TA, et al. Environmental assessment and modification to prevent falls in older people. J Am Geriatr Soc 2011;59(1):26 - 33

30~~29~~. Vernon W. The Healthy Footwear Guide. Podiatry Now 2009;5:35

31. Lamb S, Jorstad-Stein EC, Hauer K, et al. Development of a common outcome data set for fall injury prevention trials: the Prevention of Falls Network Europe consensus. J Am Geriatr Soc 2005;53(9):1618-22

For peer review only



For peer review only - http://bmjopen.bmj.com/site/about/guidelines.xhtml

32. Kind P. The EuroQoL instrument: an index of health-related quality of life. Quality of life and pharmacoeconomics in clinical trials 1996;2:191 - 201

33~~1~~. Kempen GI, Yardley L, Van Haastregt JC, et al. The Short FES-I: a shortened version of the falls efficacy scale-international to assess fear of falling. Age Ageing 2008;37(1):45 - 50

34~~2~~. J Schuling, R de Haan, M Limburg et al. The Frenchay Activities Index. Assessment of functional status in stroke patients. Stroke 1993;24:1173 – 77.

3~~3~~5. Patton MQ. Qualitative Evaluation and Research Methods. 2nd edition: Newbury Park, CA: Sage 1990

36~~4~~. Robertson MC, Campbell AJ, Herbison P. Statistical Analysis of Efficacy in Falls Prevention Trials. J Gerontol A Biol Sci Med Sci 2005;60(4):530-534

37. Silverman D. Interpreting Qualitative Data: Methods for Analysing Talk, Text and Interaction: Methods for Analyzing Talk, Text and Interaction. London, UK: Sage 1993.

3~~6~~8. Adamson J. Combined qualitative and quantitative designs. In: Bowling A, Ebrahim S eds. Handbook of health research methods: Investigation, measurement and analysis. Maidenhead UK: Open University Press 2005:230-245.

39~~7~~. National Institute for Health and Care Excellence. Guide to the methods of technology apraisal: London: NICE, 2013. http://www.nice.org.uk/article/pmg9/chapter/Foreword http://www.nice.org.uk/article/PMG9/chapter/Foreword (accessed October 2014).

40~~38~~. Brooks R. EuroQol: the current state of play. Health policy 1996;37(1):53 - 72

41~~39~~. Matthews J, Altman DG, Campbell M, et al. Analysis of serial measurements in medical research. BMJ 1990;300(6719):230

42~~0~~. Nixon RM, Thompson SG. Methods for incorporating covariate adjustment, subgroup analysis and between-centre differences into cost-effectiveness evaluations. Health Econ 2005;14(12):1217 - 29

43~~1~~. O'Hagan A, Stevens JW, Montmartin J. Bayesian cost-effectiveness analysis from clinical trial data. Stat Med 2001;20(5):733-53

For peer review only



For peer review only - http://bmjopen.bmj.com/site/about/guidelines.xhtml

44~~2~~. Willan AR, Lin D, Manca A. Regression methods for cost-effectiveness analysis with censored data. Stat Med 2005;24(1):131 - 45

45~~3~~. Stinnett AA, Mullahy J. Net health benefits: a new framework for the analysis of uncertainty in cost-effectiveness analysis. Med Decis Making 1998;18(Suppl 2):S68-80

46. Claxton K. Exploring uncertainty in cost-effectiveness analysis. Pharmacoeconomics 2008;26(9):781 - 98

4~~5~~7. Van Hout BA, Al MJ, Gordon GS, et al. Costs, effects and C/E-ratios alongside a clinical trial. Health econ 1994;3(5):309 - 19

4~~6~~8. Manca A, Palmer S. Handling missing data in patient-level cost-effectiveness analysis alongside randomised clinical trials. Appl Health Econ Health Policy 2005;4(2):65 - 75

4~~7~~9. Relton C, Torgerson D, O'Cathain A, et al. Rethinking pragmatic randomised controlled trials: introducing the "cohort multiple randomised controlled trial" design. BMJ 2010;340

For peer review only



For peer review only - http://bmjopen.bmj.com/site/about/guidelines.xhtml

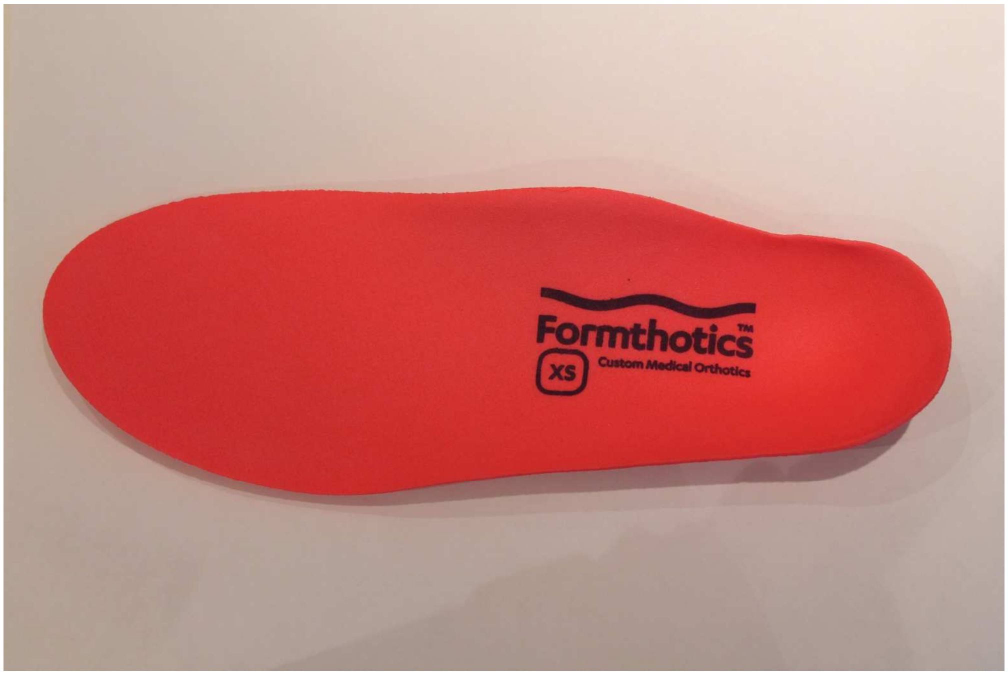


1507x1004mm (72 x 72 DPI)

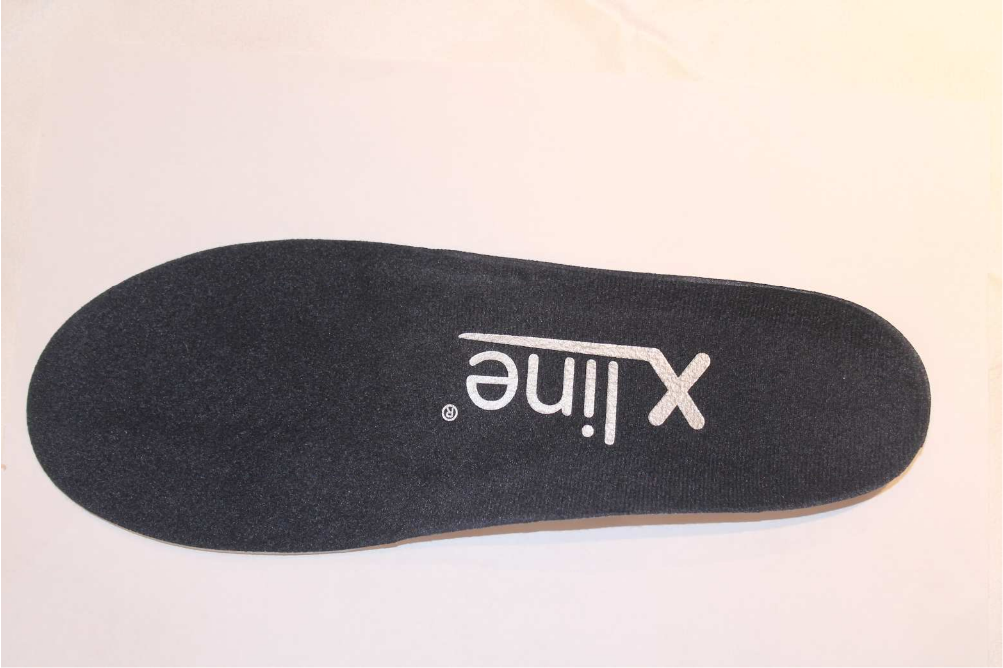


1507x1004mm (72 x 72 DPI)

eview only

SPIRIT

STANDARD PROTOCOL ITEMS: RECOMMENDATIONS FOR INTERVENTIONAL TRIALS

SPIRIT 2013 Checklist: Recommended items to address in a clinical trial protocol and related documents*

| Section/item | Item No | Description | Addressed on page number |
|---|---|---|---|
| **Administrative information** | | | |
| Title | 1 | Descriptive title identifying the study design, population, interventions, and, if applicable, trial acronym | 1 |
| Trial registration | 2a | Trial identifier and registry name. If not yet registered, name of intended registry | 4 |
| | 2b | All items from the World Health Organization Trial Registration Data Set | 1, 4, 7, 8, 10, 13, 18, 19, 31, 32 |
| Protocol version | 3 | Date and version identifier | - |
| Funding | 4 | Sources and types of financial, material, and other support | 31 |
| Roles and responsibilities | 5a | Names, affiliations, and roles of protocol contributors | 1, 31, 32 |
| | 5b | Name and contact information for the trial sponsor | 32 |
| | 5c | Role of study sponsor and funders, if any, in study design; collection, management, analysis, and interpretation of data; writing of the report; and the decision to submit the report for publication, including whether they will have ultimate authority over any of these activities | 32 |
| | 5d | Composition, roles, and responsibilities of the coordinating centre, steering committee, endpoint adjudication committee, data management team, and other individuals or groups overseeing the trial, if applicable (see Item 21a for data monitoring committee) | 32 |



For peer review only - http://bmjopen.bmj.com/site/about/guidelines.xhtml

| | | | |
|---|---|---|---|
| **Introduction** | | | |
| Background and rationale | 6a | Description of research question and justification for undertaking the trial, including summary of relevant studies (published and unpublished) examining benefits and harms for each intervention | 5-7 |
| | 6b | Explanation for choice of comparators | 5-7 |
| Objectives | 7 | Specific objectives or hypotheses | 8 |
| Trial design | 8 | Description of trial design including type of trial (eg, parallel group, crossover, factorial, single group), allocation ratio, and framework (eg, superiority, equivalence, noninferiority, exploratory) | 7 |
| **Methods: Participants, interventions, and outcomes** | | | |
| Study setting | 9 | Description of study settings (eg, community clinic, academic hospital) and list of countries where data will be collected. Reference to where list of study sites can be obtained | 8 |
| Eligibility criteria | 10 | Inclusion and exclusion criteria for participants. If applicable, eligibility criteria for study centres and individuals who will perform the interventions (eg, surgeons, psychotherapists) | 10 |
| Interventions | 11a | Interventions for each group with sufficient detail to allow replication, including how and when they will be administered | 13-17 |
| | 11b | Criteria for discontinuing or modifying allocated interventions for a given trial participant (eg, drug dose change in response to harms, participant request, or improving/worsening disease) | N/A |
| | 11c | Strategies to improve adherence to intervention protocols, and any procedures for monitoring adherence (eg, drug tablet return, laboratory tests) | 17 |
| | 11d | Relevant concomitant care and interventions that are permitted or prohibited during the trial | N/A |
| Outcomes | 12 | Primary, secondary, and other outcomes, including the specific measurement variable (eg, systolic blood pressure), analysis metric (eg, change from baseline, final value, time to event), method of aggregation (eg, median, proportion), and time point for each outcome. Explanation of the clinical relevance of chosen efficacy and harm outcomes is strongly recommended | 18-19 |
| Participant timeline | 13 | Time schedule of enrolment, interventions (including any run-ins and washouts), assessments, and visits for participants. A schematic diagram is highly recommended (see Figure) | 13, 31 |



For peer review only - http://bmjopen.bmj.com/site/about/guidelines.xhtml

| | | | |
|---|---|---|---|
| Sample size | 14 | Estimated number of participants needed to achieve study objectives and how it was determined, including clinical and statistical assumptions supporting any sample size calculations | 11-12, 19 |
| Recruitment | 15 | Strategies for achieving adequate participant enrolment to reach target sample size | 8-9 |
| **Methods: Assignment of interventions (for controlled trials)** | | | |
| Allocation: | | | |
| Sequence generation | 16a | Method of generating the allocation sequence (eg, computer-generated random numbers), and list of any factors for stratification. To reduce predictability of a random sequence, details of any planned restriction (eg, blocking) should be provided in a separate document that is unavailable to those who enrol participants or assign interventions | 12 |
| Allocation concealment mechanism | 16b | Mechanism of implementing the allocation sequence (eg, central telephone; sequentially numbered, opaque, sealed envelopes), describing any steps to conceal the sequence until interventions are assigned | 12 |
| Implementation | 16c | Who will generate the allocation sequence, who will enrol participants, and who will assign participants to interventions | 12 |
| Blinding (masking) | 17a | Who will be blinded after assignment to interventions (eg, trial participants, care providers, outcome assessors, data analysts), and how | 13 |
| | 17b | If blinded, circumstances under which unblinding is permissible, and procedure for revealing a participant's allocated intervention during the trial | N/A |
| **Methods: Data collection, management, and analysis** | | | |
| Data collection methods | 18a | Plans for assessment and collection of outcome, baseline, and other trial data, including any related processes to promote data quality (eg, duplicate measurements, training of assessors) and a description of study instruments (eg, questionnaires, laboratory tests) along with their reliability and validity, if known. Reference to where data collection forms can be found, if not in the protocol | 18,19 |
| | 18b | Plans to promote participant retention and complete follow-up, including list of any outcome data to be collected for participants who discontinue or deviate from intervention protocols | 17 |



For peer review only - http://bmjopen.bmj.com/site/about/guidelines.xhtml

| | | | |
|---|---|---|---|
| Data management | 19 | Plans for data entry, coding, security, and storage, including any related processes to promote data quality (eg, double data entry; range checks for data values). Reference to where details of data management procedures can be found, if not in the protocol | 13 |
| Statistical methods | 20a | Statistical methods for analysing primary and secondary outcomes. Reference to where other details of the statistical analysis plan can be found, if not in the protocol | 22-25 |
| | 20b | Methods for any additional analyses (eg, subgroup and adjusted analyses) | 23-24 |
| | 20c | Definition of analysis population relating to protocol non-adherence (eg, as randomised analysis), and any statistical methods to handle missing data (eg, multiple imputation) | 22, 25 |
| **Methods: Monitoring** | | | |
| Data monitoring | 21a | Composition of data monitoring committee (DMC); summary of its role and reporting structure; statement of whether it is independent from the sponsor and competing interests; and reference to where further details about its charter can be found, if not in the protocol. Alternatively, an explanation of why a DMC is not needed | 31 |
| | 21b | Description of any interim analyses and stopping guidelines, including who will have access to these interim results and make the final decision to terminate the trial | 22 |
| Harms | 22 | Plans for collecting, assessing, reporting, and managing solicited and spontaneously reported adverse events and other unintended effects of trial interventions or trial conduct | 20 |
| Auditing | 23 | Frequency and procedures for auditing trial conduct, if any, and whether the process will be independent from investigators and the sponsor | N/A |
| **Ethics and dissemination** | | | |
| Research ethics approval | 24 | Plans for seeking research ethics committee/institutional review board (REC/IRB) approval | 4 |
| Protocol amendments | 25 | Plans for communicating important protocol modifications (eg, changes to eligibility criteria, outcomes, analyses) to relevant parties (eg, investigators, REC/IRBs, trial participants, trial registries, journals, regulators) | 29 |



For peer review only - http://bmjopen.bmj.com/site/about/guidelines.xhtml

| | | | |
|---|---|---|---|
| Consent or assent | 26a | Who will obtain informed consent or assent from potential trial participants or authorised surrogates, and how (see Item 32) | 9-10 |
| | 26b | Additional consent provisions for collection and use of participant data and biological specimens in ancillary studies, if applicable | N/A |
| Confidentiality | 27 | How personal information about potential and enrolled participants will be collected, shared, and maintained in order to protect confidentiality before, during, and after the trial | 10 |
| Declaration of interests | 28 | Financial and other competing interests for principal investigators for the overall trial and each study site | 31 |
| Access to data | 29 | Statement of who will have access to the final trial dataset, and disclosure of contractual agreements that limit such access for investigators | 32 |
| Ancillary and post-trial care | 30 | Provisions, if any, for ancillary and post-trial care, and for compensation to those who suffer harm from trial participation | - |
| Dissemination policy | 31a | Plans for investigators and sponsor to communicate trial results to participants, healthcare professionals, the public, and other relevant groups (eg, via publication, reporting in results databases, or other data sharing arrangements), including any publication restrictions | 29 |
| | 31b | Authorship eligibility guidelines and any intended use of professional writers | 31 |
| | 31c | Plans, if any, for granting public access to the full protocol, participant-level dataset, and statistical code | 32 |
| **Appendices** | | | |
| Informed consent materials | 32 | Model consent form and other related documentation given to participants and authorised surrogates | - |
| Biological specimens | 33 | Plans for collection, laboratory evaluation, and storage of biological specimens for genetic or molecular analysis in the current trial and for future use in ancillary studies, if applicable | N/A |

*It is strongly recommended that this checklist be read in conjunction with the SPIRIT 2013 Explanation & Elaboration for important clarification on the items. Amendments to the protocol should be tracked and dated. The SPIRIT checklist is copyrighted by the SPIRIT Group under the Creative Commons "Attribution-NonCommercial-NoDerivs 3.0 Unported" license.



For peer review only - http://bmjopen.bmj.com/site/about/guidelines.xhtml
